# Supplementary material for: Structure-guided bifunctional molecules hit a DEUBAD-lacking hRpn13 species upregulated in multiple myeloma
Source: Nat Commun. 2021 Dec 16;12:7318. doi: 10.1038/s41467-021-27570-4 (PMC8677766; doi:10.1038/s41467-021-27570-4)

**Supplementary Information**

# Structure-guided bifunctional molecules hit a DEUBAD-lacking hRpn13 species upregulated in multiple myeloma

Xiuxiu Lu[1], Venkata R. Sabbasani[2], Vasty Osei-Amponsa[1], Christine N. Evans[3], Julianna C. King[3], Sergey G. Tarasov[4], Marzena Dyba[4], Sudipto Das[5], King C. Chan[5], Charles D. Schwieters[6], Sulbha Choudhari[7], Caroline Fromont[8], Yongmei Zhao[7], Bao Tran[8], Xiang Chen[1], Hiroshi Matsuo[9], Thorkell Andresson[5], Raj Chari[3], Rolf E. Swenson[2], Nadya I. Tarasova[10], Kylie J. Walters[1,*]

**Supplementary Table 1. Twenty-two lead compounds identified from an in silico screen as putative hRpn13 binders.**

| Nomenclature | ID | Validation Method | $\lambda_{350}$ (normalized) |
|---|---|---|---|
| DMSO | D1435 or DLM-34-10X0.75 | NMR, DSF, ITC | 100 ± 3.0 |
| **XL1** | CAS:860-22-0 | DSF | 81.5 ± 2.9 |
| **XL2** | Z119018758 | DSF | 86.1 ± 0.3 |
| **XL3** | Z231949652 | DSF | 67.9 ± 3.0 |
| **XL4** | Z17870320 | DSF | 64.8 ± 1.1 |
| **XL5** | Z44395247 | NMR, DSF, ITC | 47.2 ± 0.5 |
| **XL6** | Z45668530 | NMR, DSF | 78.4 ± 1.1 |
| **XL7** | Z2301703555 | NMR, DSF | 80.2 ± 1.5 |
| **XL8** | Z57354452 | DSF | 76.2 ± 0.6 |
| **XL9** | Z199467950 | DSF | 83.1 ± 1.8 |
| **XL10** | Z56774971 | DSF | 82.1 ± 1.6 |
| **XL11** | Z211851080 | DSF | 85.9 ± 0.4 |
| **XL12** | Z87597408 | DSF | 84.6 ± 0.8 |
| **XL13** | Z146687966 | DSF | 87.7 ± 1.7 |
| **XL14** | Z96452288 | DSF | 84.4 ± 5.9 |
| **XL15** | Z217127446 | DSF | 75.0 ± 0.8 |
| **XL16** | Z2154430633 | NMR, DSF | 81.1 ± 1.6 |
| **XL17** | Z1082901996 | NMR, DSF | 76.9 ± 1.2 |
| **XL18** | Z1917789752 | NMR, DSF | 78.5 ± 0.8 |
| **XL19** | Z2154430997 | NMR, DSF | 65.1 ± 5.1 |
| **XL20** | Z2910888840 | NMR, DSF | 86.2 ± 5.0 |
| **XL21** | Z1262429908 | NMR | N/A |
| **XL22** | Z3039488982 | NMR | N/A |
| **RA190** | M60163-2s | DSF, ITC | 76.7 ± 2.1 |

Nomenclature, Enamine ID (**XL2**-**XL22**), CAS number (**XL1**), Xcessbio ID (**RA190**, positive control), Sigma-Aldrich or Cambridge Isotope Laboratories, Inc ID (DMSO, negative control), biophysical method used to screen compounds, and normalized intrinsic tryptophan fluorescence emission is listed in column 1, 2, 3, and 4 respectively. Emission of intrinsic tryptophan fluorescence was measured at 350 nm in triplicate for 1 μM hRpn13 Pru or with addition of 20-fold molar excess of listed compound. The average

fluorescence intensity at 350 nm for each sample was normalized to hRpn13 Pru with DMSO addition and is presented with the standard deviation from the mean in column 4. N/A, not available.

**Supplementary Table 2. Chemical shift assignments for hRpn13-bound XL5.**

| **XL5** | chemical shift (ppm) |
|---|---|
| $CH_3$ | 2.376 |
| H4, H7 | 7.360 |
| H5, H6 | 7.688 |
| H8 | 10.274 |
| H12 | 7.258 |
| H13 | 3.648 |
| H15 | 7.934 |
| H16 | 6.802 |
| H17 | 7.054 |
| H18 | 7.866 |
| H19 | 4.672 |

**Supplementary Table 3. NOE interactions detected between hRpn13 and XL5.**

| hRpn13 | **XL5** |
|---|---|
| M31 $CH_3$ | H15, H19 |
| L33 H$\beta$ | H17, H18 |
| L33 H$\gamma$ | H17, H18 |
| L33 $CH_3$ | H16, H17, H18 |
| V38 $CH_3$ | H17, H18 |
| T39 $CH_3$ | H4/H7, H5/H6, $CH_3$ |
| V85 $CH_3$ | H13, H19 |
| V93 $CH_3$ | H13, H15, H19 |

**Supplementary Table 4. Contacts between hRpn13 and XL5 of the XL5-ligated hRpn13 structure measured to be within 6 Å.**

| XL5 | hRpn13 |
|---|---|
| $CH_3$ | T37 ($CH_3$); T39 (Hα, Hγ1, $CH_3$); P40 (Hγ#, Hδ#) |
| H4 | T37 ($CH_3$); V38 (Hβ, $CH_3$); T39 (HN, Hα, Hβ, Hγ1, $CH_3$); P40 (Hβ#, Hγ#, Hδ#) |
| H5 | T37 ($CH_3$); V38 (HN, Hα, Hβ, $CH_3$); T39 (HN, Hα, Hγ1, $CH_3$); P40 (Hα, Hβ#, Hγ#, Hδ#) |
| H6 | T37 ($CH_3$); V38 (HN, Hβ) |
| H7 | T37 ($CH_3$) |
| H8 | M31 (Hγ#); V38 (HN, Hα, Hβ, $CH_3$); T39 (HN, Hα); P40 (Hβ#, Hγ#, Hδ#) |
| H9 | V38 (HN, Hβ, $CH_3$); P89 (Hδ#) |
| H10 | V38 (Hβ, $CH_3$); Q87 (Hβ#); C88 (Hα, Hβ#); P89 (Hγ#, Hδ#) |
| H11 | V38 ($CH_3$); V85 ($CH_3$); Q87 (Hα, Hβ#); C88 (HN, Hα, Hβ#); P89 (Hγ#, Hδ#); S90 (HN); V93($CH_3$) |
| H12 | M31 (Hβ#, Hγ#); V38 (Hβ, $CH_3$); T39 (Hα); P40 (Hβ#, Hγ#, Hδ#); C88 (Hα, Hβ#); P89 (Hδ#); S90 (Hγ); V93($CH_3$) |
| H13 | V38 ($CH_3$); V85 (Hβ, $CH_3$); Q87 (Hβ#); C88 (HN, Hα, Hβ#); P89 (Hδ#); S90 (HN, Hβ#); V93(Hβ, $CH_3$) |
| H14 | M31 (Hγ#); V38 (Hβ, $CH_3$); 85 (Hβ, $CH_3$); Q87 (Hβ#); C88 (Hα, Hβ#); V93(Hβ, $CH_3$), F106 (Hβ#, Hδ#) |
| H15 | M31 (HN, Hα, Hβ#, Hγ#, $CH_3$); S32 (HN); L33 (Hγ); V38 ($CH_3$); V93(Hβ, $CH_3$); F106 (HN, Hα, Hβ#; Hδ#, Hε#) |
| H16 | M31 (HN, Hα, Hβ#, Hγ#); S32 (HN, Hα); L33 (HN, Hα, Hγ, $CH_3$); V38 ($CH_3$); T39 (HN); L105 (Hα); F106 (HN, Hα, Hβ#, Hδ#, Hε#, Hζ) |
| H17 | M31 (Hβ#); S32 (Hα); L33 (HN, Hα, Hβ#, Hγ, $CH_3$); V38 (Hα, $CH_3$); F106 (Hβ#, Hδ#, Hε#, Hζ) |
| H18 | L33 (Hα, Hβ#, Hγ, $CH_3$); V38 (Hα, Hβ, $CH_3$); F106 (Hδ#, Hε#, Hζ) |
| H19 | M31 (Hγ#, $CH_3$); V38 ($CH_3$); V85 (Hβ, $CH_3$); C88 (HN, Hα, Hβ#); P89 (Hδ#); S90 (HN, Hβ#, Hγ); G91 (HN); V93 (HN, Hα, Hβ, $CH_3$); F106 (Hβ#, Hδ#); W108 (Hβ#) |
| cyanide | M31 (Hγ#, $CH_3$); V38 ($CH_3$); P40 (Hβ#, Hγ#, Hδ#); C88 (Hβ#); P89 (Hδ#); S90 (HN, Hβ#, Hγ); V93 (Hβ, $CH_3$); W108 (Hβ#) |

**Supplementary Table 5. Binding affinity of XL5 derivatives for hRpn13 Pru measured by ITC.**

| 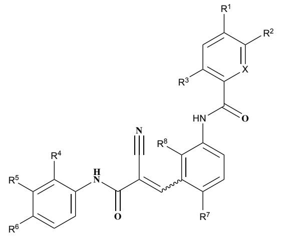 | X | $R^1$ | $R^2$ | $R^3$ | $R^4$ | $R^5$ | $R^6$ | $R^7$ | $R^8$ | NMR | ITC $K_d$ ($\mu$M) |
|---|---|---|---|---|---|---|---|---|---|---|---|
| **XL5** | C | $CH_3$ | H | H | COOH | H | H | H | H | ++++++ | 1.48 ± 0.52 |
| **XL23** | C | H | H | Cl | COOH | H | H | H | H | ++++ | 3.88 ± 0.43 |
| **XL24** | C | $-NHCH_3$ | H | H | COOH | H | H | H | H | +++++ | 1.74 ± 0.35 |
| **XL25** | C | $-NHCH_2COOH$ | H | H | COOH | H | H | H | H | +++++ | 4.12 ± 1.47 |
| **XL26** | C | $CF_3$ | H | H | COOH | H | H | H | H | +++++ | 6.67 ± 1.97 |
| **XL27** | N | $CH_3$ | OH | H | COOH | H | H | H | H | +++++ | 3.94 ± 1.02 |
| **XL28** | C | $CH_3$ | H | H | COOH | H | $-OCH_3$ | H | H | ++++ | 3.82 ± 0.26 |
| **XL29** | C | $CH_3$ | H | H | COOH | H | $-NHCH_3$ | H | H | ++++ | 7.81 ± 1.28 |
| **XL30** | C | $CH_3$ | H | H | COOH | $CF_3$ | H | H | H | ++ | 12.39 ± 5.91 |
| **XL31** | C | $CH_3$ | H | H | $-SO_2NH_2$ | H | H | H | H | + | NA |
| **XL32** | C | $CH_3$ | H | H | COOH | H | H | $CF_3$ | H | +++ | NA |
| **XL33** | C | $CH_3$ | H | H | COOH | H | H | H | $-NHCH_3$ | + | NA |

The $K_d$ value was generated by fitting ITC data to a "One Set of Sites" binding model with the Origin software. Degree of spectral changes in 2D NMR spectra is indicated by number of '+' symbols, as compared to **XL5**, with reduced effects symbolized by a lesser number.

**Supplementary Table 6. Six candidate sgRNAs designed by using the sgRNA Scorer 2.0 web tool.**

| ID | Target site (PAM sequence underlined) | Used for KO expts |
|---|---|---|
| 2286 | GGGCGCCTCCAACAAGTACT**TGG** | N |
| 2287 | TACTTGGTGGAGTTTCGGGC**GGG** | N |
| 2288 | GTGACTCCGGATAAGCGGAA**AGG** | Y |
| 2289 | TGACTCCGGATAAGCGGAAA**GGG** | N |
| 2290 | TCCGGATAAGCGGAAAGGGC**TGG** | Y |
| 2291 | GCTGGAAGGACAGGACGTCC**GGG** | N |

## Supplementary Table 7. Primers for PCR.

| Primer name | Sequence |
|---|---|
| *ADRM1*-Amp-F | **TCCCTACACGACGCTCTTCCGATCT** CTCTCCGCGCTTTCAGGATG |
| *ADRM1*-Amp-R | **GTTCAGACGTGTGCTCTTCCGATCT** CAGGGACACTCACGTCTTCC |
| *ADRM1*-Topo-F | GCAGCCAAGACGAGAAGGTG |
| *ADRM1*-Topo-R | TCCGTACTCTGGGAGAGGAC |

Red = Illumina-specific primer sequence

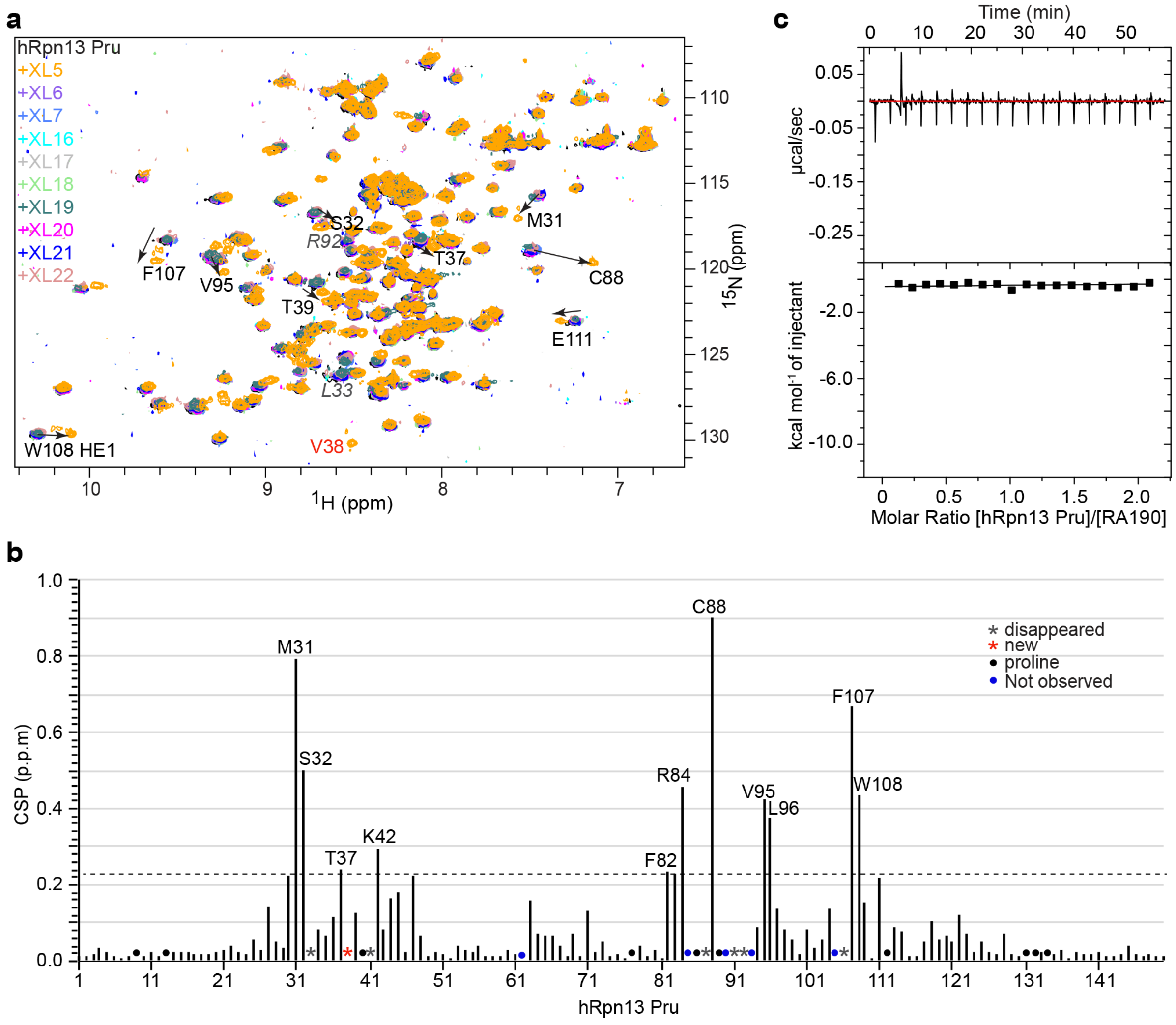


**Supplementary Fig. 1 | NMR screen identifies XL5 as binding to hRpn13. a**, $^1$H, $^{15}$N HSQC spectra of $^{15}$N-labeled hRpn13 Pru with addition of vehicle control DMSO (black) or 10-fold molar excess **XL5** (orange), **XL6** (purple), **XL7** (light blue), **XL16** (cyan), **XL17** (grey), **XL18** (green), **XL19** (dark green), **XL20** (magenta), **XL21** (blue) or **XL22** (pink). Spectra were acquired at 600 MHz and 25°C. hRpn13 signals that shift following **XL5** addition are labeled and a solid arrow indicates trajectory from the free to **XL5**-bound state. Some signals that disappear (italicized grey) or V38 (red), which appears, following **XL5** addition are also labeled. **b**, Chemical shift perturbation (CSP) values derived from

the data of Fig. 1b for each hRpn13 Pru amino acid following **XL5** addition. Residues with signals that disappear or appear are denoted with a grey or red star respectively. Prolines or residues not observed for free and **XL5**-bound hRpn13 are indicated with a black or blue dot respectively. A dashed line indicates one standard deviation above average. Residues shifted by greater than one standard deviation above average are labeled. **c**, ITC analysis of hRpn13 Pru binding to **RA190**. Raw ITC data (top) from titration of 200 μM hRpn13 Pru into 20 μM **RA190** and binding isotherm (bottom) created by integration of the raw data.

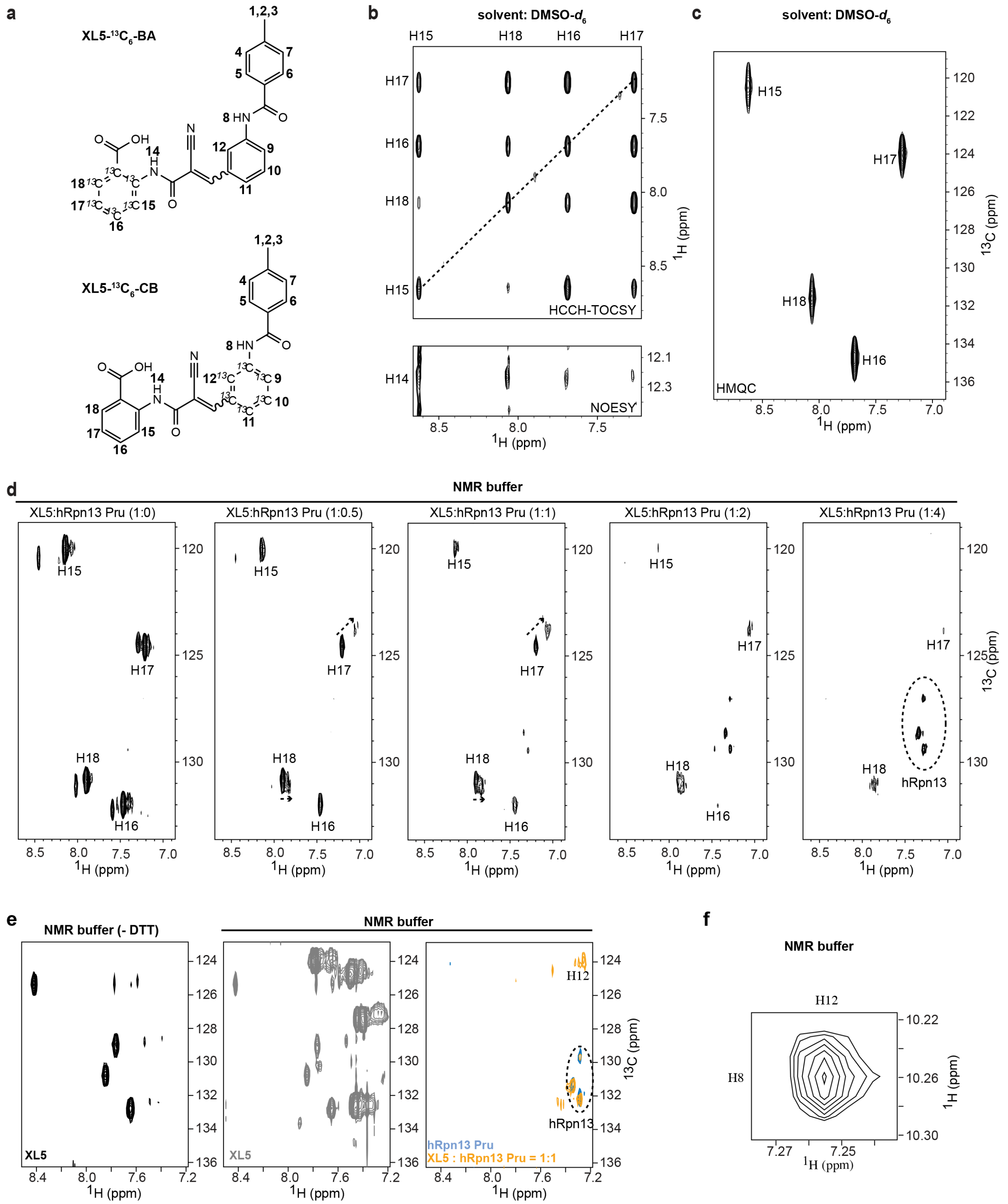


**Supplementary Fig. 2 | Chemical assignments for XL5 and after mixing with hRpn13 Pru. a**, The chemical structure of **XL5** illustrating isotopic labeling of the benzoic acid carbon atoms (**XL5**-$^{13}C_6$-BA, top panel) or central benzene carbon atoms (**XL5**-$^{13}C_6$-CB,

bottom panel); these labeling schemes were used for the spectra shown in **b-d** or **e-f** respectively. Hydrogen atoms are labeled with the numbers used in the text and figures. **b-c**, 2D $^{13}C$-edited HCCH-TOCSY with a 12 ms mixing time (**b**, top panel), NOESY with a 500 ms mixing time (**b**, bottom panel), and $^{1}H$, $^{13}C$ HMQC spectra (**c**) recorded on 10 mM **XL5**-$^{13}C_6$-BA (**a**, top panel) in DMSO-$d_6$ at 25 °C. Diagonal signals in **b** are indicated by a dashed line. **d**, $^{1}H$, $^{13}C$ HMQC spectra recorded on 0.1 mM **XL5**-$^{13}C_6$-BA (**a**, top panel) with increasing molar ratio of unlabeled hRpn13 Pru, including at 1:0 (first panel), 1:0.5 (second panel), 1:1 (third panel), 1:2 (fourth panel), and 1:4 (fifth panel) in NMR buffer. Signal shifting is indicated by a dashed arrow that extends from the free state to the hRpn13 Pru-bound state. Attenuation of the **XL5** signals is observed with hRpn13 Pru addition; this effect is consistent with **XL5** $\pi$-$\pi$ stacking with F106 as illustrated in the later shown structure. **e**, $^{1}H$, $^{13}C$ HMQC spectra recorded on 0.5 mM **XL5**-$^{13}C_6$-CB (**a**, bottom panel) in NMR buffer without (left panel, black) or with (middle panel, grey) DTT, or in NMR buffer and mixed with equimolar unlabeled hRpn13 Pru (right panel, orange), or on 0.5 mM unlabeled hRpn13 Pru with no **XL5** (right panel, blue) in NMR buffer. **f**, Selected regions from a $^{1}H$, $^{13}C$ half-filtered 2D NOESY experiment (100 ms) recorded on 0.5 mM **XL5**-$^{13}C_6$-CB (**a**, bottom panel) with equimolar unlabeled hRpn13 Pru in NMR buffer. Natural abundance carbon signals arising from hRpn13 Pru in **d** and **e** are indicated by a dashed oval.

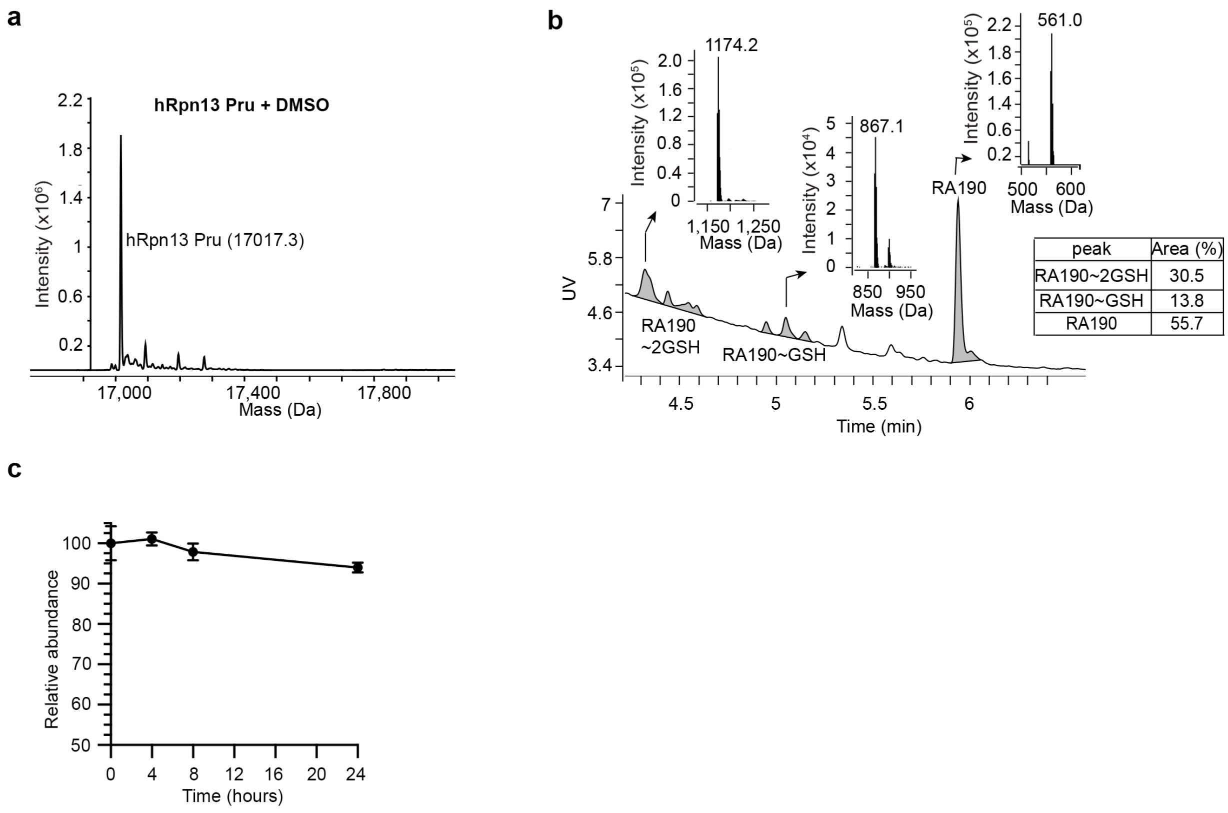


**Supplementary Fig. 3 |. LC-MS spectra examining reactivity of RA190 and XL5. a**, LC-MS analysis of 2 μM purified hRpn13 Pru (MW: 17017.3 g/mol) incubated with DMSO for 2 hours at 4°C. **b**, LC-MS analysis of 40 μM **RA190** incubated with 2 mM reduced L-glutathione (GSH, MW: 307.3 g/mol) for 2 hours at 4°C. Detected GSH adducts are indicated and a table included that lists relative abundance. **c**, LC-MS analyses at indicated time points of 0.2 μM **XL5** incubated at room temperature with commercially available mouse serum. Data represent mean ± SD of n = 6 biological replicates. Relative abundance is plotted as (absolute abundance)$_{\text{time point}}$/(absolute abundance)$_{\text{time=0}}$*100 (%).

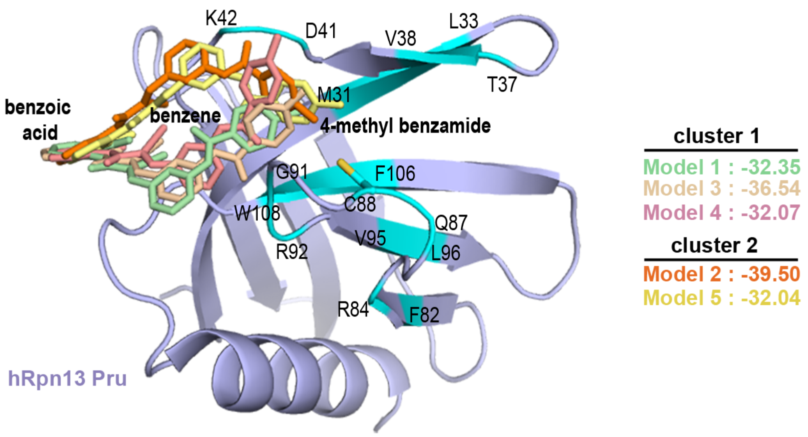


**Supplementary Fig. 4 |. Model structures of XL5 bound to hRpn13 from the *in silico* screen.** Ribbon diagram of the predicted model structures with **XL5** (green in Model 1, orange in Model 2, wheat in Model 3, pink in Model 4, yellow in Model 5) bound to hRpn13 Pru (purple). Model structures are divided into two clusters based on the location of the central benzene ring, with the virtual ligand screening score calculated in ICM (Internal Coordinate Mechanics, Molsoft LLC) listed for each model. hRpn13 amino acids significantly affected by **XL5** addition in Fig. 1b are highlighted in light blue as described in Fig. 1c.

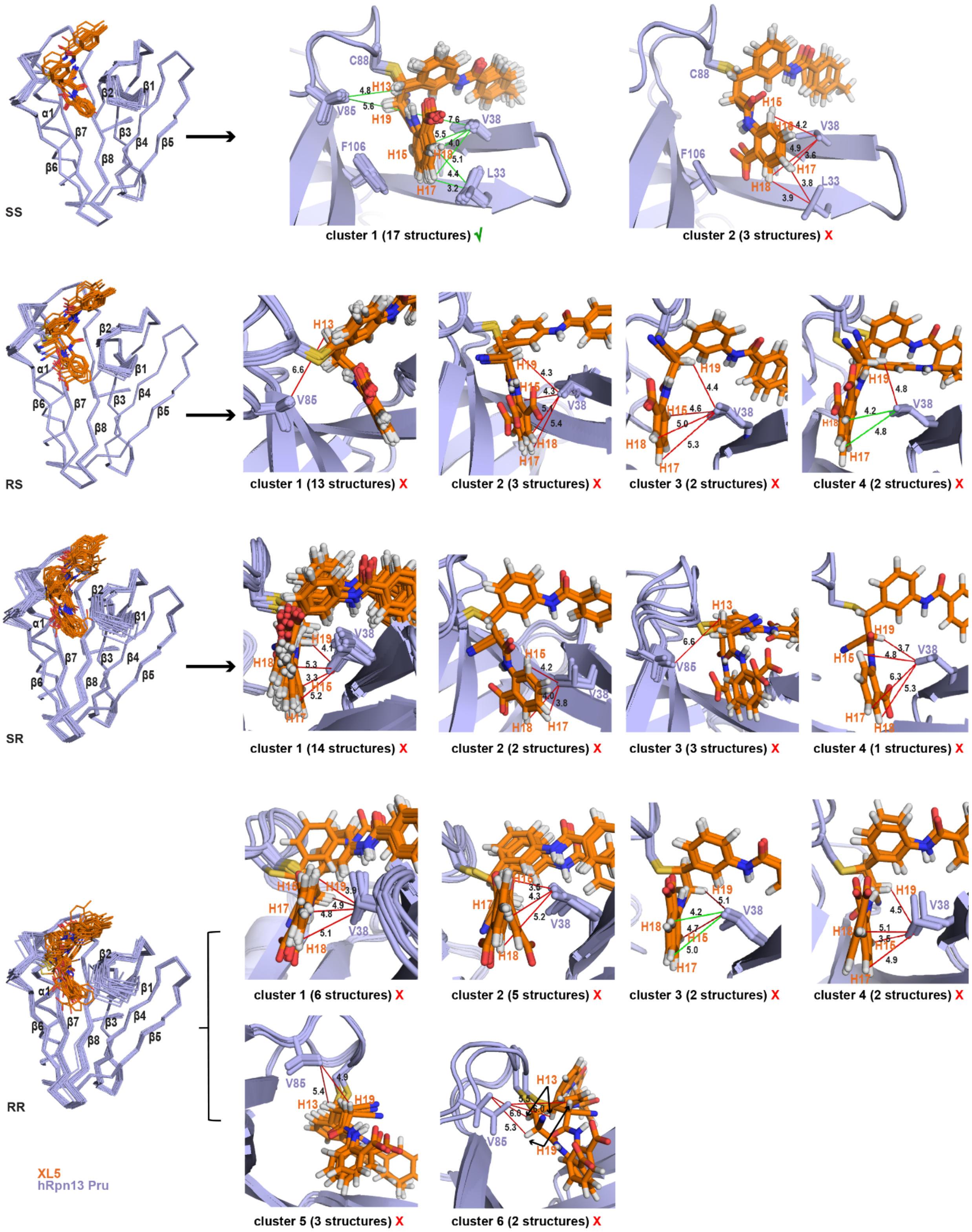


**Supplementary Fig. 5 |. Calculated structures of XL5-ligated hRpn13 Pru in different stereoisomer conformations.** hRpn13 with **XL5** ligated to the C88 sulfur atom and **XL5**

C15 and C16 of respective SS (first panel), RS (second panel), SR (third panel) or RR (fourth panel) stereochemistry, colored as in Fig. 2g. The lowest energy structures without NOE, dihedral or torsion angle violations were clustered based on convergence. Right panels display enlarged views for each cluster centered on **XL5** H13, H15, H17, H18, and H19 along with distances between atoms in Å. The number of structures within each cluster is displayed below the panel along with a green check mark (√) to indicate that the displayed interactions are supported (green lines) by the NMR data or a red x to indicate differential interactions that are not supported (red lines) by the NMR data.

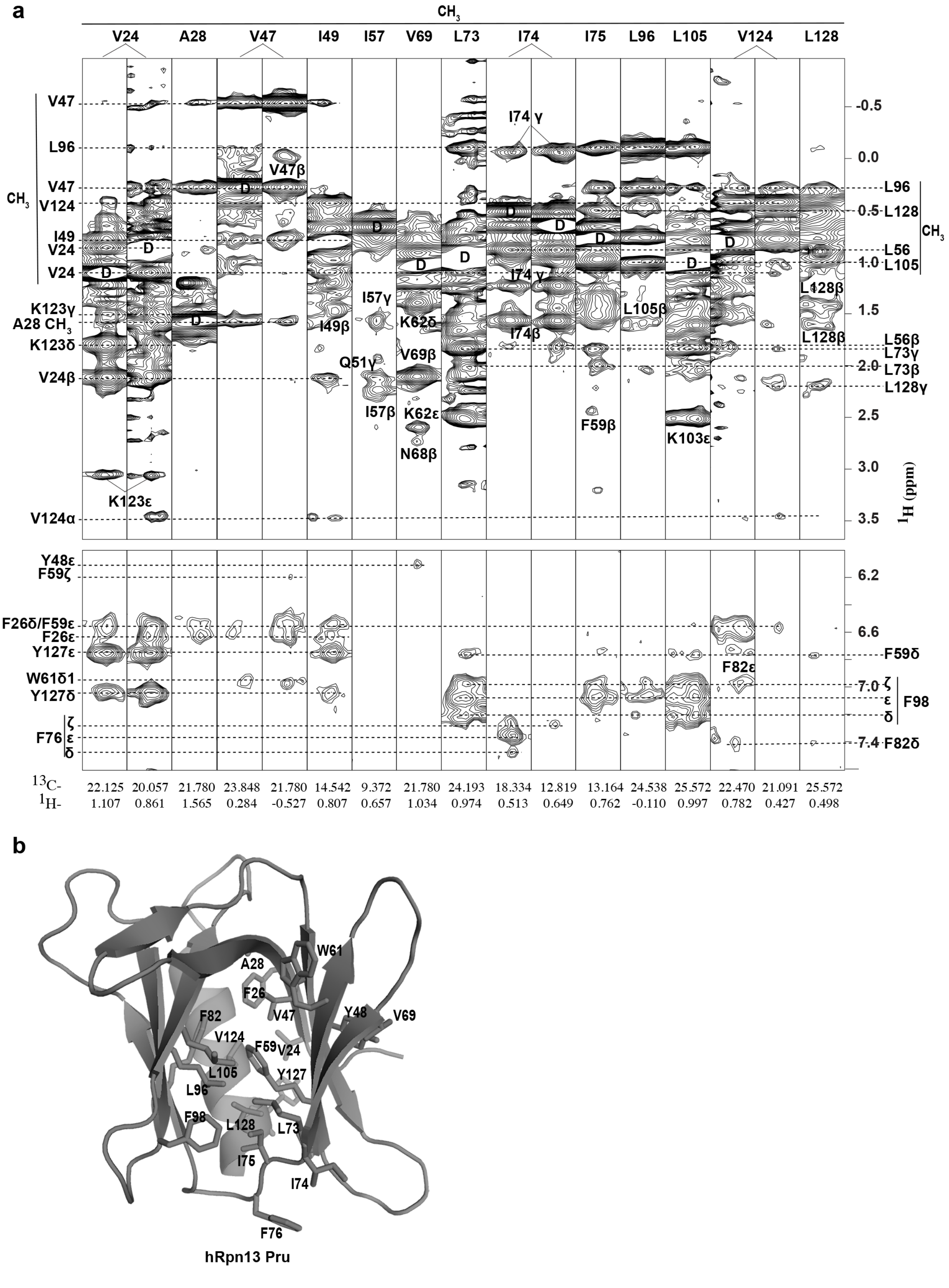
a
CH3
V24
A28
V47
I49
I57
V69
L73
I74
I75
L96
L105
V124
L128
V47
L96
V47
V124
I49
V24
V24
K123γ
A28 CH3
K123δ
V24β
K123ε
V124α
V47β
I74 γ
I57γ
I49β
K62δ
V69β
Q51γ
I57β
K62ε
N68β
I74 γ
I74β
F59β
L105β
K103ε
L128β
L128β
D
L96
L128
L56
L105
CH3
L56β
L73γ
L73β
L128γ
-0.5
0.0
0.5
1.0
1.5
2.0
2.5
3.0
3.5
1H (ppm)
Y48ε
F59ζ
F26δ/F59ε
F26ε
Y127ε
W61δ1
Y127δ
F76
ζ
ε
δ
6.2
6.6
7.0
7.4
F59δ
F82ε
F98
F82δ
13C-
1H-
22.125 20.057 21.780 23.848 21.780 14.542 9.372 21.780 24.193 18.334 12.819 13.164 24.538 25.572 22.470 21.091 25.572
1.107 0.861 1.565 0.284 -0.527 0.807 0.657 1.034 0.974 0.513 0.649 0.762 -0.110 0.997 0.782 0.427 0.498
b
A28
W61
F26
V47
Y48
V69
F82
V124
F59
V24
L105
Y127
L96
F98
L128
L73
I75
I74
F76
hRpn13 Pru

**Supplementary Fig. 6 | The hRpn13 Pru structure is preserved in the presence of XL5. a**, Selected intramolecular NOEs for **XL5**-bound hRpn13 Pru from a $^{1}$H, $^{13}$C edited NOESY experiment (mixing time 100 ms) acquired with 0.4 mM $^{13}$C-labeled hRpn13 Pru and 1.2-fold molar excess unlabeled **XL5**. D, diagonal resonance. **b**, Residues showing NOE interactions in **a** are displayed on a ribbon diagram of free hRpn13 Pru (grey, PDB 5IRS). The amino acids displayed in **a** belong to the structural core and show the expected interactions for structural integrity.

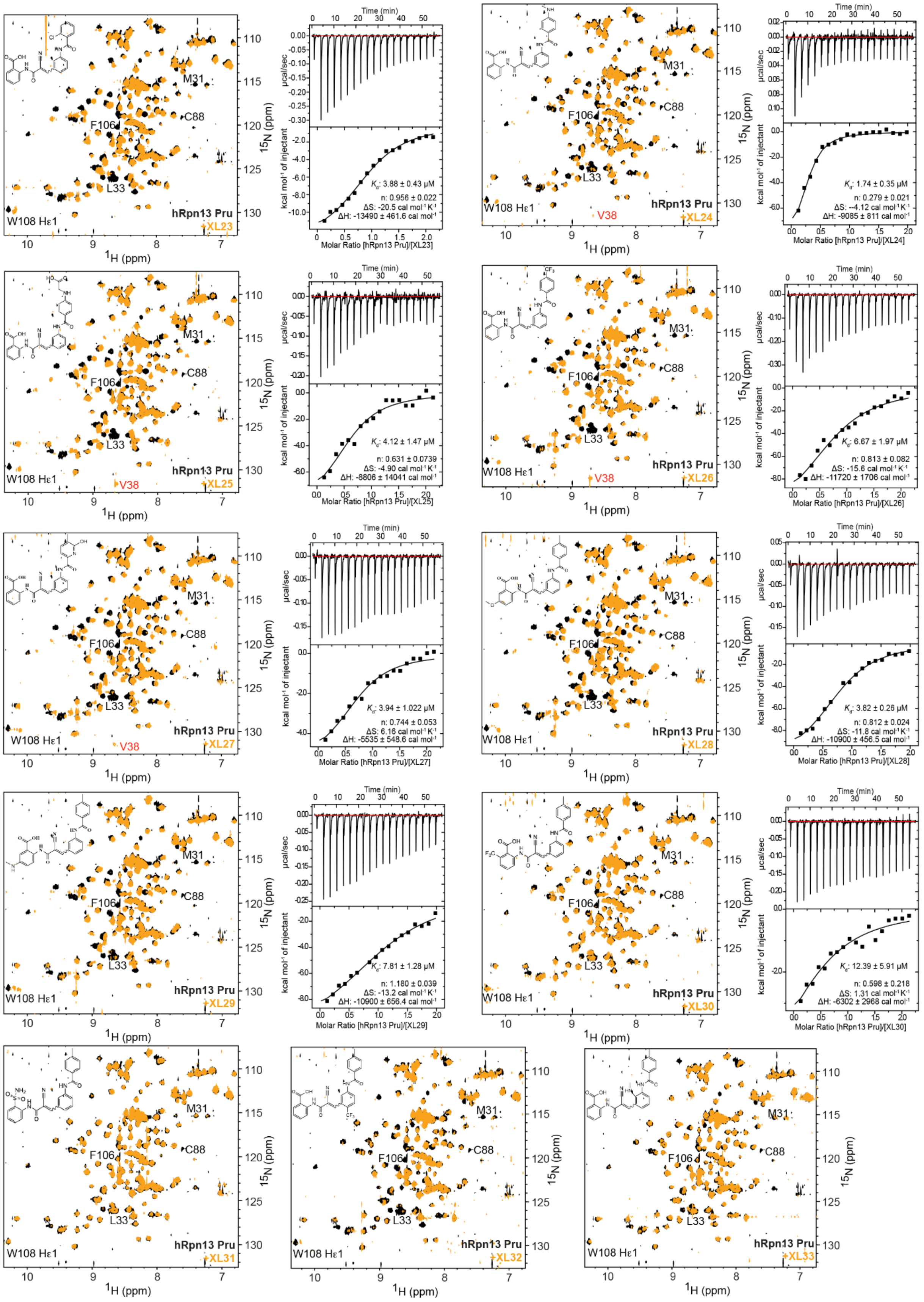

hRpn13 Pru
XL23
W108 Hε1
M31
C88
F106
L33
$^{1}$H (ppm)
$^{15}$N (ppm)
Time (min)
µcal/sec
kcal mol⁻¹ of injectant
Molar Ratio [hRpn13 Pru]/[XL23]
Kd: 3.88 ± 0.43 µM
n: 0.956 ± 0.022
ΔS: -20.5 cal mol⁻¹ K⁻¹
ΔH: -13490 ± 461.6 cal mol⁻¹
XL24
V38
Molar Ratio [hRpn13 Pru]/[XL24]
Kd: 1.74 ± 0.35 µM
n: 0.279 ± 0.021
ΔS: -4.12 cal mol⁻¹ K⁻¹
ΔH: -9085 ± 811 cal mol⁻¹
XL25
Molar Ratio [hRpn13 Pru]/[XL25]
Kd: 4.12 ± 1.47 µM
n: 0.631 ± 0.0739
ΔS: -4.90 cal mol⁻¹ K⁻¹
ΔH: -8806 ± 14041 cal mol⁻¹
XL26
Molar Ratio [hRpn13 Pru]/[XL26]
Kd: 6.67 ± 1.97 µM
n: 0.813 ± 0.082
ΔS: -15.6 cal mol⁻¹ K⁻¹
ΔH: -11720 ± 1706 cal mol⁻¹
XL27
Molar Ratio [hRpn13 Pru]/[XL27]
Kd: 3.94 ± 1.022 µM
n: 0.744 ± 0.053
ΔS: 6.16 cal mol⁻¹ K⁻¹
ΔH: -5535 ± 548.6 cal mol⁻¹
XL28
Molar Ratio [hRpn13 Pru]/[XL28]
Kd: 3.82 ± 0.26 µM
n: 0.812 ± 0.024
ΔS: -11.8 cal mol⁻¹ K⁻¹
ΔH: -10900 ± 456.5 cal mol⁻¹
XL29
Molar Ratio [hRpn13 Pru]/[XL29]
Kd: 7.81 ± 1.28 µM
n: 1.180 ± 0.039
ΔS: -13.2 cal mol⁻¹ K⁻¹
ΔH: -10900 ± 656.4 cal mol⁻¹
XL30
Molar Ratio [hRpn13 Pru]/[XL30]
Kd: 12.39 ± 5.91 µM
n: 0.598 ± 0.218
ΔS: 1.31 cal mol⁻¹ K⁻¹
ΔH: -6302 ± 2968 cal mol⁻¹
XL31
XL32
XL33

**Supplementary Fig. 7 |. NMR and ITC analyses of hRpn13 binding to XL5 derivatives.** $^{1}$H, $^{15}$N HSQC spectra of 20 μM $^{15}$N-labeled hRpn13 Pru with addition of vehicle control DMSO (black) or 10-fold molar excess **XL5** derivative **XL23**-**XL33** (orange), as indicated, and accompanying ITC data for **XL5** derivatives **XL23**-**XL30**. NMR spectra were acquired at 10°C and 600 MHz for **XL23**-**XL32** and 800 MHz for **XL33**. The signal for hRpn13 V38 is labeled in red when it appears. **XL5** binding residues hRpn13 M31, L33, C88, F106, W108 are labeled. ITC analyses are presented to the right of the corresponding NMR data when available with the top panel plotting raw ITC data and the bottom panel displaying the binding isotherm generated by integrating the raw data. ITC titrations were performed with 200 μM hRpn13 Pru injected into 20 μM **XL5** derivative. The data was fit to a "One Set of Sites" binding model with the indicated thermodynamic values by using the Origin software. The chemical structure of each **XL5** derivative is included within the NMR spectra.

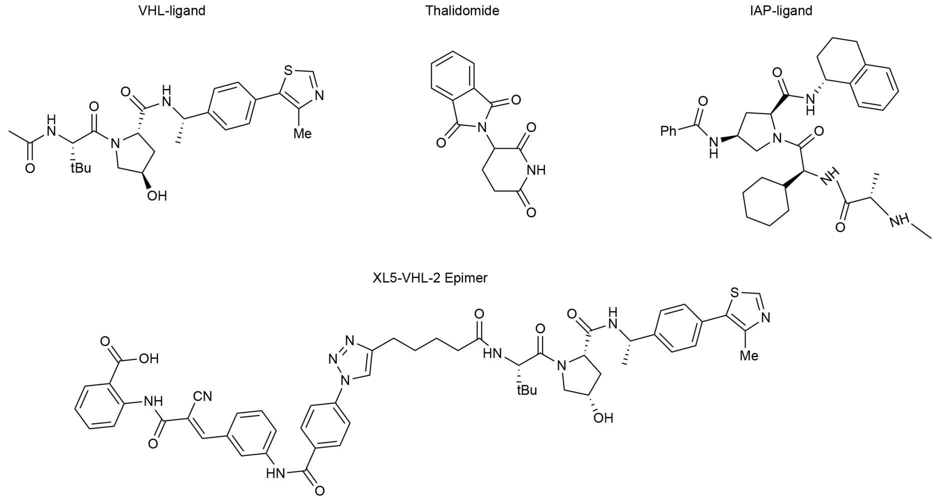


**Supplementary Fig. 8 |. Chemical structures of the VHL-ligand, thalidomide, IAP-ligand, and the XL5-VHL-2 Epimer.**

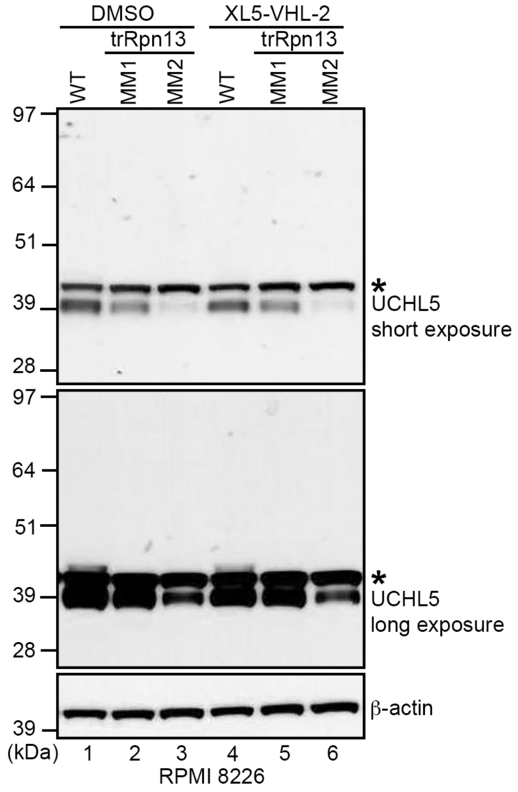


**Supplementary Fig. 9 |. UCHL5 does not appear to be targeted by XL5-VHL-2.** Immunoblots of whole cell lysate from RPMI 8226 WT, trRpn13-MM1, or trRpn13-MM2 cells treated for 24 hours with 40 μM **XL5-VHL-2** with comparison to DMSO (vehicle control) immunoprobing for UCHL5 (short and long exposure), or β-actin (as a loading control, bottom panel). A black asterisk indicates an unknown band that may be non-specific.

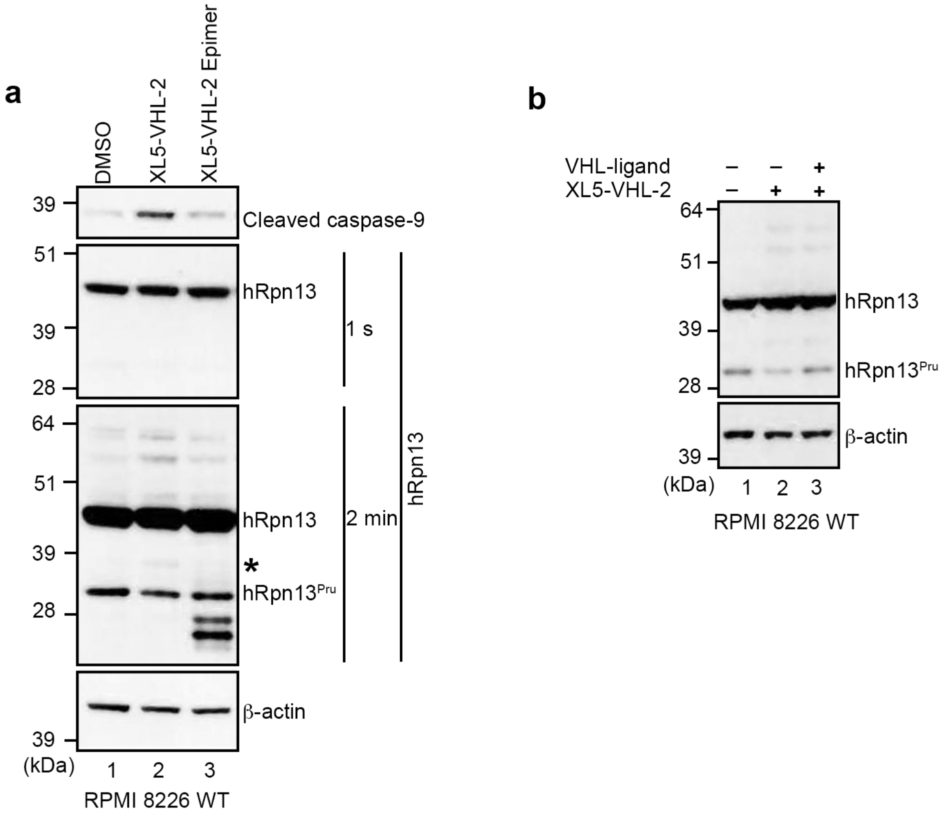


**Supplementary Fig. 10 |. Degradation of hRpn13$^{Pru}$ by XL5-VHL-2 is mediated through VHL. a,** Immunoblots of whole cell extract from RPMI 8226 WT cells treated for 24 hours with 40 μM **XL5-VHL-2** or **XL5-VHL-2** Epimer compared to DMSO (vehicle control) detecting cleaved caspase-9, hRpn13 (1-second and 2-minute exposure) or β-actin. A black asterisk indicates cleaved caspase-9 in the 2-minute immunoblot for hRpn13, as hRpn13 was probed following cleaved caspase-9 and without stripping the membrane. **b,** Immunoblots of whole cell extract from RPMI 8226 WT cells treated for 24 hours with 40 μM **XL5-VHL-2** with or without 40 μM **VHL-ligand** or DMSO (control) detecting hRpn13 or β-actin.

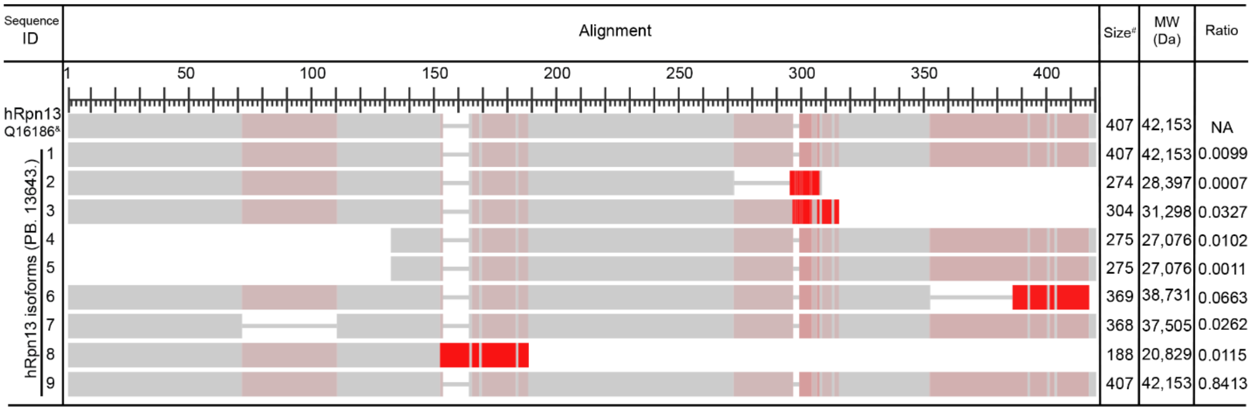


# Size provided as amino acid number
& UniProtKB number of hRpn13

**Supplementary Fig. 11 |. Protein sequence alignment of hRpn13 isoforms in RPMI 8226 WT cells.** The protein sequence of hRpn13 isoforms translated from mRNA transcripts identified by mRNA PacBio were aligned with Protein Blast (https://blast.ncbi.nlm.nih.gov/BlastAlign.cgi) and colored based on frequency-based amino acid variance. Darker shades of red indicate greater difference from residues in other rows of the alignment at that position. Included is sequence ID, size (number of amino acids), molecular weight (MW) and ratio of hRpn13 mRNA expression for each isoform as quantified by Illumina. The mRNA samples were isolated from RPMI 8226 WT cells and analyzed in triplicate.

**Supplementary Note 1**

**General information for chemical synthesis.** Starting materials were used as received unless otherwise noted. All moisture sensitive reactions were performed in an inert atmosphere of argon with oven dried glassware. Reagent grade solvents were used for extractions and flash chromatography. Reaction progress was monitored by LC-MS analysis performed on an Agilent UPLC/MS instrument equipped with a RP-C18 column (Poroshell 120 SB-C18, 4.6 X 50 mm, 2.7 µm or Zorbax 300SB-C18, 4.6 X 50 mm, 3.5 µm), dual atmospheric pressure chemical ionization (APCI)/electrospray (ESI) mass spectrometry detector, and photodiode array detector. Flash chromatography was performed by using a RediSepRf NP-silica (40-63 µm 60 Å) or a Teledyne RediSepRf Gold RP-C18 column (20-40 µm 100 Å) in a Teledyne ISCO CombiFlash Rf 200 purification system unless otherwise specified. $^{1}$H NMR spectra were recorded on an Agilent 400 MHz or Bruker 800 MHz spectrometer and are reported in parts per million (ppm) on the δ scale relative to $CDCl_3$ (δ 7.26) and DMSO-$d_6$ (δ 2.50) as internal standards. Data are reported as follows: chemical shift, multiplicity (s = singlet, d = doublet, t = triplet, q = quartet, b = broad, m = multiplet), coupling constants (Hz), and integration. $^{13}$C-NMR spectra were recorded on an Agilent 100 MHz or Bruker 200 MHz spectrometer and are reported in parts per million (ppm) on the δ scale relative to $CDCl_3$ (δ 77.00) and DMSO-$d_6$ (δ 39.52). Note: The recorded $^{1}$H NMRs of $^{13}C_6$ labeled compounds are very complex and difficult to interpret due to large couplings between proton and $^{13}$C-carbon. To remove the large couplings of H-$^{13}$C, the BilevelDec $^{1}$H NMR

method was used for all $^{13}C_6$ labeled compounds and both the $^1H$ NMR and BilevelDec $^1H$ NMR data are reported.

**Synthesis and characterization data.** **XL5**-$^{13}C_6$-CB, **XL25**, **XL26**, **XL27**, **XL30**, **XL31**, **XL32**, **XL33**, **XL5-VHL**, **XL5-VHL-2**, **XL5-CRBN** and **XL5-IAP** were synthesized according to the procedures described below and characterization data ($^1H$ NMR, $^{13}C$ NMR, $^{19}F$ NMR and high-resolution mass spectrometry (HRMS)) are included.

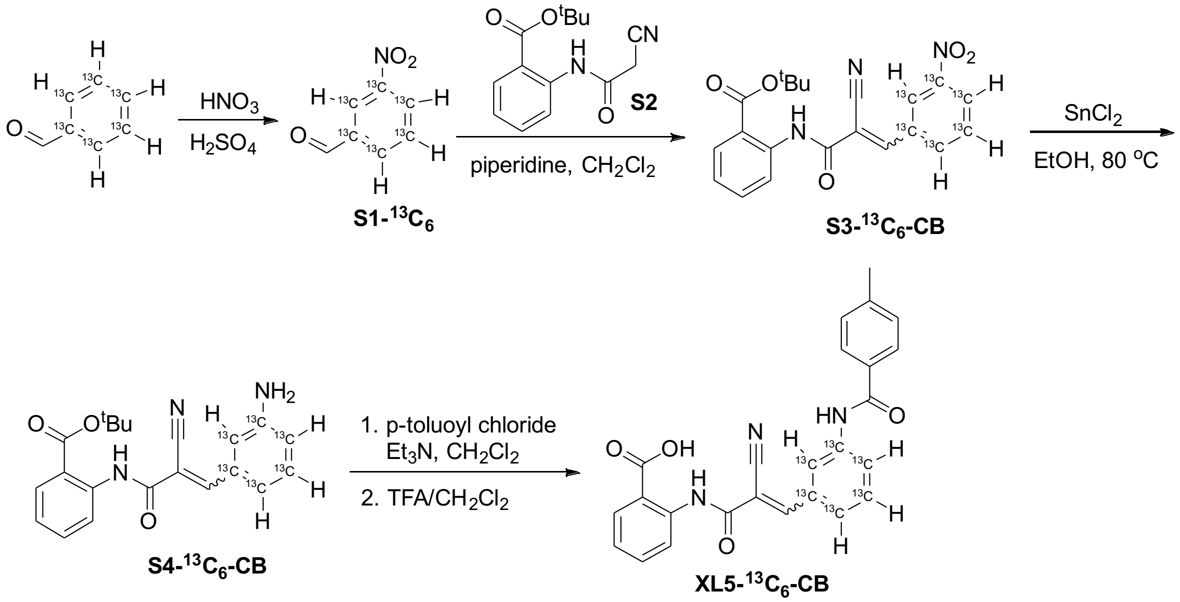


Benz-$^{13}C_6$-aldehyde (500 mg, 4.46 mmol) was placed into a 100 mL round bottom flask and then cold 90% nitric acid (8 mL) was added at –30°C. The resulting yellow solution was warmed to –10°C and stirred for 30 min. Ice water (20 mL) and EtOAc (30 mL) were then added to the reaction mixture. The separated organic layer was washed with water (2 * 15 mL), aqueous $NaHCO_3$ (15 mL), and aqueous NaCl (20 mL). The collected organic layer was dried ($Na_2SO_4$) and concentrated to provide a crude oil. The crude material was purified by an ISCO combi flash silica gel column (hexanes/EtOAc) to provide 3-nitro-

benz-$^{13}C_6$-aldehyde **S1**-$^{13}C_6$ (430 mg, 61%). Characterization data of **S1**-$^{13}C_6$: $^1$H NMR (400 MHz, $CDCl_3$): δ 10.10 (dt, *J* = 25.0, 2.1 Hz, 1H), 8.95 – 8.58 (m, 1H), 8.51 – 8.37 (m, 1H), 8.30 – 7.45 (m, 2H); BilevelDec $^1$H NMR (400 MHz, $CDCl_3$): δ 10.13 (d, *J* = 0.8 Hz, 1H), 8.72 (d, *J* = 2.5 Hz, 1H), 8.54 – 8.41 (m, 1H), 8.23 (dt, *J* = 7.8, 1.3 Hz, 1H), 7.77 (t, *J* = 8.1 Hz, 1H); $^{13}$C NMR (101 MHz, $CDCl_3$): δ 189.91 (m), 148.72 (td), 137.38 (td), 134.70 (m), 130.32 (m), 128.46 (ddd), 124.28 (ddd).

In a thick-walled vial, 3-nitro-benz-$^{13}C_6$-aldehyde **S1**-$^{13}C_6$ (200 mg, 1.27 mmol) was dissolved in $CH_2Cl_2$ (3 mL) and then **S2** (331 mg, 1.27 mmol) and piperidine (10 drops) were added at room temperature. The vial was sealed and stirring was continued for 6 hours at room temperature. The yellow solid product was collected, washed with $CH_2Cl_2$ (2 x 10 mL) and dried under vacuum to afford yellow solid **S3**-$^{13}C_6$-CB (465 mg, 91%, single *E*-isomer). $^1$H NMR (400 MHz, $CDCl_3$): δ 12.40 (s, 1H), 9.02 – 8.52 (m, 3H), 8.44 (t, *J* = 5.1 Hz, 1H), 8.25 – 8.10 (m, 1H), 8.04 (dd, *J* = 8.0, 1.7 Hz, 1H), 7.99 – 7.45 (m, 2H), 7.18 (ddd, *J* = 8.2, 7.3, 1.2 Hz, 1H), 1.64 (s, 9H); BilevelDec $^1$H NMR (400 MHz, $CDCl_3$): δ 12.39 (s, 1H), 8.77 (s, 1H), 8.72 (dd, *J* = 8.5, 1.1 Hz, 1H), 8.44 (s, 1H), 8.43 – 8.34 (m, 2H), 8.04 (dd, *J* = 8.0, 1.7 Hz, 1H), 7.73 (t, *J* = 8.0 Hz, 1H), 7.58 (ddd, *J* = 8.7, 7.3, 1.7 Hz, 1H), 7.18 (ddd, *J* = 8.1, 7.4, 1.2 Hz, 1H), 1.65 (s, 10H); $^{13}$C NMR (101 MHz, $CDCl_3$): δ 167.57, 158.29 (d), 149.84 (m), 148.61 (td), 140.33, 135.26 (dddd), 134.05, 133.38 (td), 131.12, 130.38 (td), 126.59 (m), 125.46 (m), 123.81, 120.94, 117.87, 115.01 (d), 109.51, 83.19, 28.17; HRMS (*m/z*): $[M+Na]^+$ calcd. for $C_{15}{}^{13}C_6H_{19}N_3O_5Na$, 422.1424; found, 422.1421 (APCI).

In a thick-walled vial, nitro compound **S3**-$^{13}C_6$-CB (85 mg, 0.21 mmol) in EtOH (5 mL) was treated with $SnCl_2$ (202 mg, 1.06 mmol) under argon atmosphere at room temperature. The vial was sealed and heated at 80°C for 1 hour, after which LC-MS indicated the complete consumption of starting material **S3**-$^{13}C_6$-CB. The cooled reaction mixture was quenched with aqueous sodium bicarbonate (20 mL) and the product extracted with EtOAc (2 x 15 mL) and dried ($Na_2SO_4$). After concentration the crude product was purified by an ISCO combi flash silica gel column (EtOAc/hexanes) to provide yellow colored aniline derivative **S4**-$^{13}C_6$-CB (62 mg, 80%). $^1$H NMR (400 MHz, $CDCl_3$): δ 12.19 (s, 1H), 8.72 (ddd, *J* = 8.4, 1.2, 0.4 Hz, 1H), 8.28 (t, *J* = 5.7 Hz, 1H), 8.02 (ddd, *J* = 8.0, 1.7, 0.5 Hz, 1H), 7.70 – 6.51 (m, 6H), 3.78 (bs, 2H), 1.64 (s, 9H); BilevelDec $^1$H NMR (400 MHz, $CDCl_3$): δ 12.19 (s, 1H), 8.72 (dd, *J* = 8.5, 1.2 Hz, 1H), 8.28 (s, 1H), 8.02 (ddd, *J* = 7.9, 1.7, 0.4 Hz, 1H), 7.56 (ddd, *J* = 8.7, 7.3, 1.7 Hz, 1H), 7.38 (d, *J* = 2.0 Hz, 1H), 7.31 (dd, *J* = 20.0, 7.6 Hz, 2H), 7.15 (ddd, *J* = 7.9, 7.3, 1.2 Hz, 1H), 6.86 (d, *J* = 7.7 Hz, 1H), 3.75 (bs, 2H), 1.64 (s, 9H); $^{13}$C NMR (101 MHz, $CDCl_3$): δ 167.47, 159.67 (d), 153.61 (d), 146.80 (ddd), 140.63, 133.90, 132.84 (ddd), 131.02, 130.03 (ddd), 123.37, 121.98 (tdd), 120.95, 119.63 (m), 117.84, 116.10, 115.80 (td), 105.42, 82.90, 28.19; HRMS (*m/z*): $[M+H]^+$ calcd. for $^{13}C_6C_{15}H_{22}N_3O_3$, 370.1862; found, 370.1860.

To a stirred solution of **S3**-$^{13}C_6$-CB (56 mg, 0.15 mmol) in $CH_2Cl_2$, p-toluoyl chloride (24 μL, 0.18 mmol) and $Et_3N$ (44 μL, 0.30 mmol) were added at room temperature. Stirring was continued for 12 hours, quenched with water (5 mL), and the product extracted with $CH_2Cl_2$ (2 x 6 mL) and dried ($Na_2SO_4$). The filtrate was concentrated under reduced pressure and purified by an ISCO combi flash silica gel column (EtOAc/hexanes) to afford

tert-butyl 2-(2-cyano-3-(3-(4-methylbenzamido)phenyl-$^{13}C_6$)acrylamido)benzoate. The tert-butyl-benzoate product was subjected to 1 mL $CH_2Cl_2$/TFA (1:1) and stirred for 1 hour at room temperature (monitored by LC-MS). The solvent and TFA were removed under reduced pressure to provide yellow solid material. The solid material was washed with dichloromethane (3 * 6 mL) to provide pure **XL5**-$^{13}C_6$-CB (52 mg, 80%). $^1H$ NMR (400 MHz, DMSO-$d_6$): δ 13.95 (s, 1H), 12.25 (s, 1H), 10.43 (d, $J$ = 3.2 Hz, 1H), 8.70 – 7.54 (m, 10H), 7.34 (d, $J$ = 8.0 Hz, 2H), 7.28 – 7.20 (m, 1H), 2.38 (s, 3H); BilevelDec $^1H$ NMR (400 MHz, DMSO-$d_6$): δ 13.94 (s, 1H), 12.24 (s, 1H), 10.42 (s, 1H), 8.62 (dd, $J$ = 8.5, 1.2 Hz, 1H), 8.46 (s, 1H), 8.37 (s, 1H), 8.05 (dd, $J$ = 7.9, 1.7 Hz, 1H), 7.94 (d, $J$ = 8.0 Hz, 1H), 7.89 (d, $J$ = 8.1 Hz, 2H), 7.78 (d, $J$ = 7.7 Hz, 1H), 7.67 (ddd, $J$ = 8.7, 7.3, 1.7 Hz, 1H), 7.56 (t, $J$ = 7.9 Hz, 1H), 7.34 (d, $J$ = 8.0 Hz, 2H), 7.25 (td, $J$ = 7.6, 1.2 Hz, 1H), 2.38 (s, 3H); $^{13}C$ NMR (101 MHz, DMSO-$d_6$): δ 170.23, 166.09, 159.45 (d), 153.29 (d), 142.33, 140.65, 140.48 (ddd), 134.74, 132.50 (ddd), 132.15 (d), 131.67, 130.06 (td), 129.41, 128.24, 125.54 (dtd), 124.25, 122.46 (ddd), 120.83, 117.51, 106.53, 21.49; HRMS ($m/z$): $[M+H]^+$ calcd. for $^{13}C_6C_{19}H_{20}N_3O_4$, 432.1655; found, 432.1660.

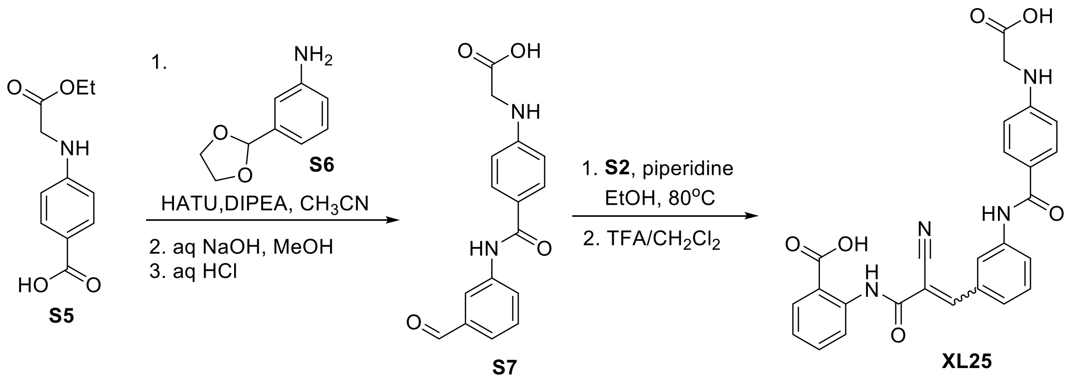


In a 25 mL round bottom flask, 4-((2-Ethoxy-2-oxoethyl)amino)benzoic acid **S7** (300 mg, 1.34 mmol) was dissolved in $CH_3CN$ (6 mL) and cooled to 0°C. HATU (612 mg, 1.61 mmol), **S6** (254 mg, 1.5 mmol), and DIPEA (0.7 mL, 4 mmol) were added to the cooled

solution. The reaction mixture was stirred for 30 min at room temperature at which point LC-MS indicated the complete consumption of **S5**. The solvent was evaporated, water was added to the reaction mixture and product extracted with EtOAC (2 x 10 mL) dried over anhydrous $Na_2SO_4$. After concentration, the crude product was dissolved in MeOH (2 mL) and then aqueous NaOH (2 mL, 1 M) was added at room temperature. The mixture was heated for 3 hours at 50$^o$C (monitored by LCMS) and cooled to room temperature. Ice cold aqueous HCl (4 mL, 1 M) was added at room temperature and stirred for 1 hour. The reaction mixture extracted with EtOAC (4 x 10 mL) and the combined organic layers were washed with aqueous NaCl (20 mL) and dried over anhydrous $Na_2SO_4$. After concentration, the crude product was purified by an ISCO combi flash silica gel column ($CH_2Cl_2$/MeOH) to afford aldehyde **S7** (150 mg, 37%). $^1$H NMR (400 MHz, DMSO-$d_6$): δ 10.09 (s, 1H), 9.99 (s, 1H), 8.36 (t, *J* = 1.9 Hz, 1H), 8.06 (ddd, *J* = 7.9, 2.3, 1.4 Hz, 1H), 7.81 (d, *J* = 8.9 Hz, 2H), 7.68 – 7.43 (m, 2H), 6.65 (d, *J* = 8.8 Hz, 1H), 3.91 (s, 2H); $^{13}$C NMR (101 MHz, DMSO-$d_6$): δ 193.61 (d), 172.61, 165.96, 151.89, 141.05, 137.09, 129.88, 129.81, 129.74, 126.33, 125.10, 121.73, 120.45, 120.43, 111.62, 111.55, 44.66; HRMS (*m/z*): $[M+H]^+$ calcd. for $C_{16}H_{15}N_2O_4$, 299.1032; found, 299.1033.

In a thick-walled vial, aldehyde **S7** (50 mg, 0.17 mmol) was dissolved in ethanol (2 mL) and then **S2** (44 mg, 0.17 mmol) and piperidine (10 drops) were added at room temperature. The vial was sealed and heated at 80$^o$C for 1 hour. The reaction mixture was cooled to room temperature and the solvent was removed by rotary evaporator. The crude material was purified by an ISCO combi flash silica gel column ($CH_2Cl_2$/MeOH) to afford *tert*-butyl benzoate (*E/Z* = 6:1). The benzoate was dissolved in 2 mL $CH_2Cl_2$/TFA

(1:1) and stirred for 1 hour. The product was precipitated and then washed with cold $CH_2Cl_2$ (10 mL) to provide the pure **XL25** (48 mg, 59%, *E/Z* = 6:1). Characterization data of major isomer: $^{1}$H NMR (400 MHz, DMSO-$d_6$): δ 12.23 (s, 1H), 10.10 (s, 1H), 8.63 (dd, *J* = 8.5, 1.2 Hz, 1H), 8.45 (t, *J* = 2.0 Hz, 1H), 8.41 – 8.32 (m, 1H), 8.06 (dd, *J* = 7.9, 1.7 Hz, 1H), 7.92 (ddd, *J* = 8.1, 2.0, 0.9 Hz, 1H), 7.84 – 7.77 (m, 2H), 7.77 – 7.74 (m, 1H), 7.69 (ddd, *J* = 8.7, 7.4, 1.7 Hz, 1H), 7.55 (t, *J* = 8.0 Hz, 1H), 7.27 (td, *J* = 7.6, 1.2 Hz, 1H), 6.70 – 6.59 (m, 2H), 3.90 (s, 2H); $^{13}$C NMR (101 MHz, DMSO-$d_6$): δ 172.61, 170.24, 165.97, 159.53, 153.47, 151.88, 141.01, 140.66, 134.80, 132.43, 131.70, 129.98, 129.79, 125.32, 125.13, 124.28, 122.44, 121.76, 120.87, 117.48, 116.04, 111.58, 106.37, 44.67; HRMS (*m/z*): $[M+H]^+$ calcd. for $C_{26}H_{21}N_4O_6$, 485.1461; found, 485.1463.

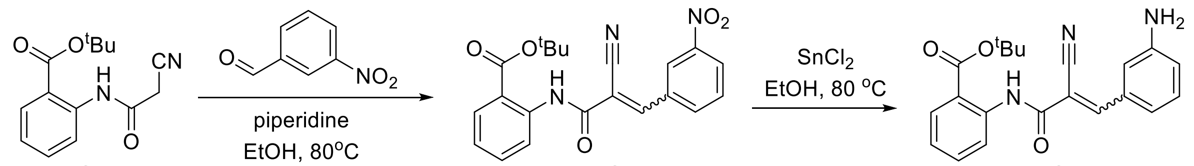

**S2** **S3** **S4**

In a thick-walled vial, 2-cyano-N-arylacetamide **S2** (1 g, 3.84 mmol) was dissolved in ethanol (10 mL) and then 3-nitrobenzaldehyde (577 mg, 3.84 mmol) and piperidine (10 drops) were added at room temperature. The vial was sealed and heated at 80°C for 1 hour. The reaction mixture was cooled to room temperature and the precipitate was collected, washed with ethanol (2 x 10 mL) and dried under vacuum to afford yellow solid **S3** (1.33 g, 89%, single *E*-isomer). $^{1}$H NMR (400 MHz, $CDCl_3$): δ 12.41 (s, 1H), 8.80 – 8.76 (m, 1H), 8.75 – 8.70 (m, 1H), 8.45 (s, 1H), 8.43 – 8.34 (m, 2H), 8.05 (dd, *J* = 8.0, 1.7 Hz, 1H), 7.78 – 7.70 (m, 1H), 7.65 – 7.52 (m, 1H), 7.19 (dddd, *J* = 8.0, 7.3, 1.2, 0.7 Hz, 1H), 1.65 (d, *J* = 0.7 Hz, 9H); $^{13}$C NMR (101 MHz, $CDCl_3$): δ 167.57, 158.30, 150.14, 148.64, 140.33, 135.24, 134.06, 133.46, 131.14, 130.43, 126.60, 125.47, 123.82, 120.95,

117.88, 115.03, 109.51, 83.20, 28.18; HRMS (*m/z*): $[M+Na]^+$ calcd. for $C_{21}H_{19}N_3O_5Na$, 416.1222; found, 416.1222 (APCI).

Nitrobenzene **S3** (800 mg, 2 mmol) in ethanol (15 mL) was placed into a thick-walled vial and $SnCl_2$ (1.89 g, 10 mmol) was added under argon atmosphere at room temperature. The sealed vial was heated at 80°C for 2 hours, after which LCMS indicated the complete consumption of starting material **S3**. The cooled reaction mixture was quenched with aqueous sodium bicarbonate (20 mL) and the product extracted with EtOAc (3 x 10 mL) and dried ($Na_2SO_4$). After concentration, the crude product was purified by an ISCO combi flash silica gel column (EtOAc/hexanes) to provide aniline derivative **S4** (545 mg, 75%). $^1$H NMR (400 MHz, $CDCl_3$): δ 12.22 (s, 1H), 8.74 (d, *J* = 8.4 Hz, 1H), 8.30 (d, *J* = 2.3 Hz, 1H), 8.03 (d, *J* = 8.0 Hz, 1H), 7.58 (t, *J* = 8.0 Hz, 1H), 7.40 (s, 1H), 7.37 – 7.26 (m, 3H), 7.16 (t, *J* = 7.7 Hz, 1H), 6.87 (d, *J* = 7.8 Hz, 1H), 3.88 (s, 2H), 1.65 (d, *J* = 2.2 Hz, 9H); $^{13}$C NMR (101 MHz, $CDCl_3$): δ 167.49, 159.71, 153.69, 147.03, 140.64, 133.94, 132.84, 131.07, 130.07, 123.39, 121.98, 120.96, 119.60, 117.82, 116.14, 115.74, 105.35, 82.93, 28.20; HRMS (*m/z*): $[M+Na]^+$ calcd. for $C_{21}H_{21}N_3O_3Na$, 386.1481; found, 386.1482.

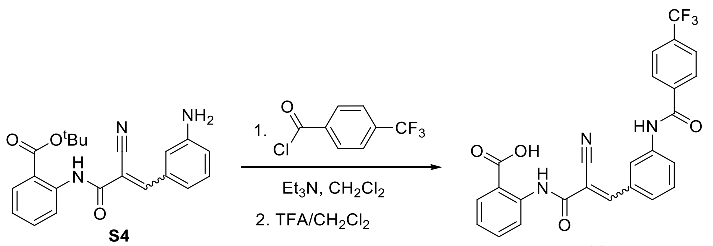


S4 XL26

**XL26:** In a 20 mL round bottom flask, compound **S4** (50 mg, 0.14 mmol) was dissolved in $CH_2Cl_2$ (3 mL). 4-(Trifluoromethyl)benzoyl chloride (32 mg, 0.15 mmol) and $Et_3N$ (30

μL, 0.21 mmol) were added at 0°C. Solvent was evaporated and purified by an ISCO combi flash silica gel column (EtOAc/hexanes). The product was dissolved in 2 mL $CH_2Cl_2$/TFA (1:1) and stirred for 3 hours at room temperature. After the deprotection was completed, the solvent was removed and purified by an ISCO combi flash silica gel column ($CH_2Cl_2$/MeOH) to provide **XL26** (30 mg, 45% over two steps). $^1$H NMR (400 MHz, DMSO-$d_6$): δ 12.31 (s, 1H), 10.75 (s, 1H), 8.62 (dd, *J* = 8.5, 1.1 Hz, 1H), 8.47 (t, *J* = 1.9 Hz, 1H), 8.39 (s, 1H), 8.19 – 8.11 (m, 2H), 8.05 (dd, *J* = 7.9, 1.7 Hz, 1H), 7.94 (ddt, *J* = 10.3, 7.6, 0.9 Hz, 3H), 7.81 (ddd, *J* = 8.3, 1.7, 0.8 Hz, 1H), 7.67 (ddd, *J* = 8.6, 7.4, 1.7 Hz, 1H), 7.60 (t, *J* = 8.0 Hz, 1H), 7.25 (td, *J* = 7.6, 1.2 Hz, 1H); $^{13}$C NMR (101 MHz, DMSO-$d_6$): δ 170.24, 165.18, 159.43, 153.13, 140.63, 140.07, 138.88, 134.69, 132.63, 131.68, 130.20, 129.17, 126.54, 125.94 (q), 125.34, 124.26, 122.43, 120.82, 117.70, 115.96, 106.76; HRMS (m/z): $[M+H]^+$ calcd. for $C_{25}H_{17}N_3O_4F_3$, 480.1171; found, 480.1173.

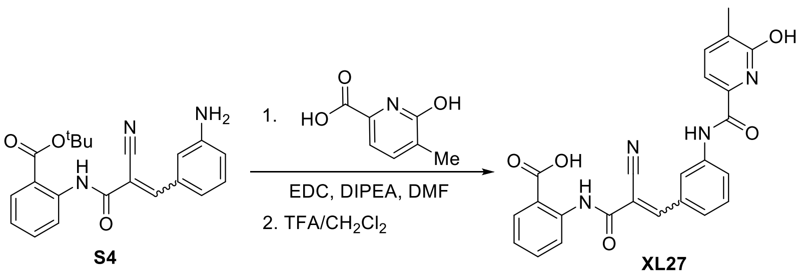


In a 10 mL round bottom flask, 6-hydroxy-5-methylpicolinic acid (52 mg, 0.34 mmol) was dissolved in DMF (2 mL). EDC-HCl salt (107 mg, 0.56 mmol), **S4** (100 mg, 0.28 mmol), and DIPEA (0.21 mL) were added at room temperature. The reaction mixture was stirred overnight and DMF was removed by rotary evaporator. The crude material was purified by an ISCO combi flash silica gel column ($CH_2Cl_2$/MeOH). Fractions were combined and the solvent was removed to provide tert-butyl benzoate derivative. tert-Butyl benzoate was subjected to 2 mL $CH_2Cl_2$/TFA (1:1) to deprotect the tert-butyl group. After 1 hour,

dichloromethane and TFA were removed by rotary evaporator to yield yellow solid. The solid was washed with $CH_2Cl_2$ (5 mL) to afford **XL27** (8 mg, 6% over two steps). $^1$H NMR (800 MHz, DMSO-$d_6$): δ 11.46 (s, 1H), 10.55 (s, 1H), 8.54 (d, *J* = 8.2 Hz, 1H), 8.35 (d, *J* = 2.4 Hz, 1H), 8.27 (s, 1H), 8.08 (dd, *J* = 7.7, 1.8 Hz, 1H), 7.94 (dd, *J* = 8.2, 2.1 Hz, 1H), 7.80 (d, *J* = 7.7 Hz, 1H), 7.60 (q, *J* = 7.9, 6.6 Hz, 2H), 7.44 (t, *J* = 7.8 Hz, 1H), 7.26 (s, 2H), 7.11 (t, *J* = 7.5 Hz, 1H), 2.14 (s, 3H); $^{13}$C NMR (201 MHz, DMSO-$d_6$) δ 169.80, 161.55, 158.90, 150.63, 140.30, 138.91, 132.35, 131.07, 129.67, 128.86, 127.68, 125.26, 123.81, 122.57, 121.60, 119.49, 119.08, 117.99, 116.49, 115.57, 115.00, 108.07, 16.18; HRMS (*m/z*): [M+H]$^+$ calcd. for $C_{24}H_{19}N_4O_5$, 443.1355; found, 443.1350.

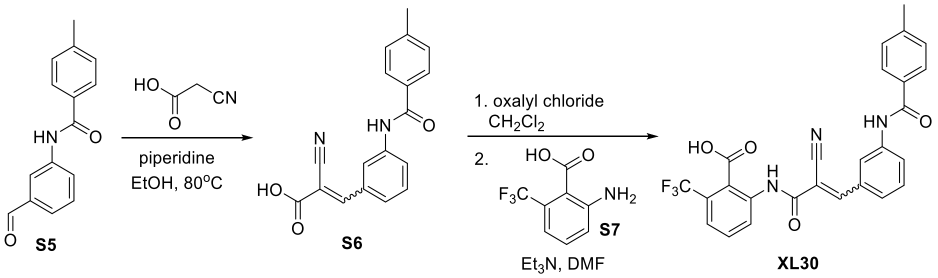


In a thick-walled vial, aldehyde **S5** (1 g, 4.18 mmol) was dissolved in ethanol and then cyanoacetic acid (355 mg, 4.18 mmol), and piperidine (10 drops) were added at room temperature. The vial was sealed and heated at 80°C for 1 hour. The reaction mixture was cooled to room temperature and the precipitate was collected, washed with ethanol (2 x 10 mL) and dried under vacuum to yield product **S6** (1.1 g, 86%, single *E*-isomer). $^1$H NMR (400 MHz, DMSO-$d_6$): δ 10.43 (s, 1H), 8.44 (t, *J* = 1.9 Hz, 1H), 8.28 (s, 1H), 7.94 (ddd, *J* = 8.2, 2.2, 1.0 Hz, 1H), 7.91 – 7.85 (m, 2H), 7.78 – 7.72 (m, 1H), 7.56 (t, *J* = 8.0 Hz, 1H), 7.41 – 7.28 (m, 2H), 2.39 (s, 3H); $^{13}$C NMR (101 MHz, DMSO-$d_6$): δ 166.08, 163.66, 154.75, 142.32, 140.44, 132.36, 132.14, 130.02, 129.42, 128.28, 126.15, 125.47,

122.59, 116.23, 104.73, 21.52; HRMS (*m/z*): $[M+H]^+$ calcd. for $C_{18}H_{15}N_2O_3$, 307.1083; found, 307.1083.

In a 25 mL round bottom flask, the above compound **S6** (100 mg, 0.33 mmol) was dissolved in $CH_2Cl_2$ (6 mL) and cooled to 0°C. Oxalyl chloride (1 mL) and DMF (1 drop) were added to the cooled solution. The reaction mixture was warmed to room temperature and stirred for 2 hours (monitored by LC-MS). The solvent and excess oxalyl chloride were removed by rotary evaporator to provide the corresponding acid chloride of **S6**. To a stirred solution of acid chloride in $CH_2Cl_2$ (5 mL), **S7** (75 mg, 0.36 mmol) and $Et_3N$ (184 µLt, 1.32 mmol) were added at 0°C. The reaction mixture was warmed to room temperature and stirred for 6 hours. The solvent was removed and purified by an RP-C18 ISCO combi flash column (Water and MeCN, water was buffered with 0.05% TFA) to yield **XL30** (60 mg, 37%). $^1H$ NMR (400 MHz, DMSO-$d_6$): δ 10.43 (s, 1H), 10.37 (s, 1H), 8.47 (t, $J$ = 1.9 Hz, 1H), 8.28 (s, 1H), 8.01 – 7.84 (m, 4H), 7.83 – 7.69 (m, 3H), 7.58 (t, $J$ = 8.0 Hz, 1H), 7.44 – 7.26 (m, 2H), 2.38 (s, 3H); $^{13}C$ NMR (101 MHz, DMSO-$d_6$) δ 167.03, 166.12, 161.30, 142.36, 140.53, 135.70, 132.49, 132.15, 131.40, 131.03, 130.11, 129.43, 128.24, 127.42, 127.11, 125.38, 125.20, 125.05, 124.64, 124.59, 122.47, 116.15, 106.74, 21.48; HRMS (*m/z*): $[M+H]^+$ calcd. for $C_{26}H_{19}N_3O_4F_3$, 494.1328; found, 494.1326.

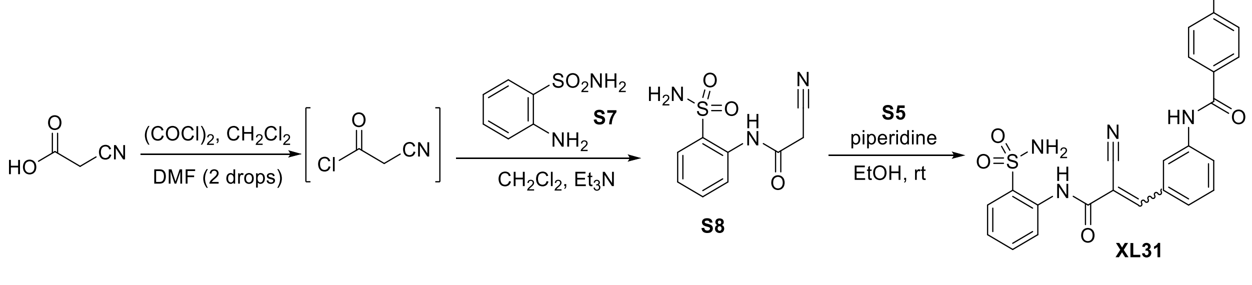

In a 25 mL round bottom flask, cyanoacetic acid (1 g, 11.75 mmol) was dissolved in $CH_2Cl_2$ (10 mL) and cooled to 0°C. Oxalyl chloride (1.3 mL, 15.27 mmol) and DMF (2 drops) were added at 0°C. The reaction mixture was warmed to room temperature and stirred for 1 hour. Solvent and excess oxalyl chloride were removed to provide 2-cyanoacetyl chloride. Cyanoacetyl chloride was dissolved in $CH_2Cl_2$ (10 mL) and cooled to 0°C. 2-Aminobenzenesulfonamide **S7** (2.02 g, 11.75 mmol) and $Et_3N$ (2.2 mL, 15.26 mmol) were added to the cooled solutions. The reaction mixture was warmed to room temperature and stirred overnight. Water (5 mL) was added to the reaction mixture and extracted with $CH_2Cl_2$ (2 x 15 mL). The combined organic layers were dried with anhydrous $Na_2SO_4$ and purified by a silica gel ISCO combi flash column ($CH_2Cl_2$/MeOH) to afford **S8** (2.1 g, 75%). $^1$H NMR (400 MHz, DMSO-$d_6$): δ 9.46 (s, 1H), 7.87 (d, $J$ = 8.0 Hz, 2H), 7.73 – 7.53 (m, 3H), 7.37 (t, $J$ = 7.8 Hz, 1H), 4.06 (s, 2H); $^{13}$C NMR (101 MHz, DMSO-$d_6$) δ 162.44, 135.17, 134.29, 133.27, 128.17, 126.14, 116.23, 27.46; HRMS (*m/z*): $[M+Na]^+$ calcd. for $C_9H_9N_3O_3SNa$, 262.0262; found, 262.0262.

In a thick-walled vial, **S8** (100 mg, 0.42 mmol) was dissolved in $CH_2Cl_2$ (3 mL) and then **S5** (100 mg, 0.42 mmol) and piperidine (10 drops) were added at room temperature. The vial was sealed and stirring was continued for 2 hours at room temperature. The solid product was collected, washed with $CH_2Cl_2$ (2 x 10 mL) and dried under vacuum to provide **XL31** (155 mg, 80%, single *E*-isomer). $^1$H NMR (400 MHz, DMSO-$d_6$): δ 10.46 (s, 1H), 10.15 (s, 1H), 8.49 (t, $J$ = 2.0 Hz, 1H), 8.36 (s, 1H), 8.20 (dd, $J$ = 8.3, 1.2 Hz, 1H), 7.97 (dd, $J$ = 8.3, 2.1 Hz, 1H), 7.94 – 7.86 (m, 3H), 7.82 – 7.79 (m, 1H), 7.76 (s, 2H), 7.68 (ddd, $J$ = 8.6, 7.5, 1.6 Hz, 1H), 7.60 (t, $J$ = 8.0 Hz, 1H), 7.41 (dd, $J$ = 7.7, 1.2 Hz, 1H), 7.37

(d, $J$ = 7.9 Hz, 2H), 2.41 (s, 3H); $^{13}C$ NMR (101 MHz, DMSO-$d_6$) δ 166.12, 159.90, 152.52, 142.36, 140.54, 134.64, 133.60, 133.44, 132.55, 132.16, 130.14, 129.47, 129.42, 128.30, 125.73, 125.51, 125.19, 124.34, 122.50, 116.11, 107.06, 21.48. $^{19}F$ NMR (376 MHz, DMSO-$d_6$) δ -57.94; HRMS ($m/z$): $[M+H]^+$ calcd. for $C_{24}H_{21}N_4O_4S$, 461.1284; found, 461.1282.

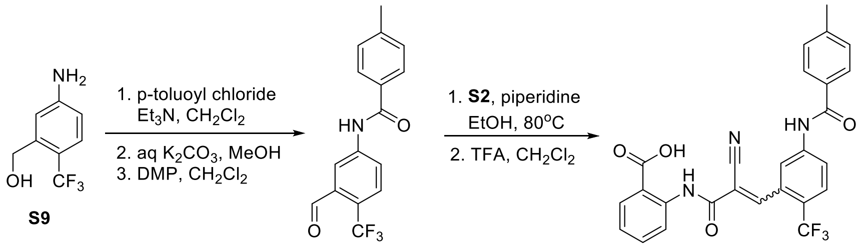

**S9** **S10** **XL32**

In a 25 mL round bottom flask, 5-amino-2-(trifluoromethyl)benzyl alcohol **S9** (250 mg, 1.31 mmol) was dissolved in $CH_2Cl_2$ (5 mL) and cooled to 0°C. p-Toluoyl chloride (506 mg, 3.27 mmol) and $Et_3N$ (0.8 mL, 5.24 mmol) were added to the cooled solution. The reaction mixture was warmed to room temperature and stirring continued overnight. Water (5 mL) was added to the reaction mixture and the product extracted with $CH_2Cl_2$ (10 mL x 2). The combined organic layers were dried over anhydrous $Na_2SO_4$, filtered, and concentrated to give the crude ditoluoylated product. The crude material was dissolved in MeOH (5 mL) and then $K_2CO_3$ (903 mg, 6.55 mmol) was added at room temperature. The reaction mixture was stirred for 30 min and filtered through a pad of celite. The solvent was removed to provide the benzyl alcohol. The crude benzyl alcohol in $CH_2Cl_2$ (10 mL) was cooled to 0°C and DMP (848 mg, 2 mmol) added. The reaction mixture was warmed to room temperature and stirred for 1 hour. The reaction mixture was quenched with aqueous $Na_2S_2O_3$ (5 mL) and product extracted with $CH_2Cl_2$ (2 x 10 mL). The combined organic layers were dried over anhydrous $Na_2SO_4$, filtered,

concentrated and purified by an ISCO Combi flash column (SiO2, hexanes/EtOAc) to yield aldehyde **S10** (215 mg, 53% over 3 steps). $^{1}$H NMR (400 MHz, $CDCl_3$): δ 10.39 (d, *J* = 2.1 Hz, 1H), 8.56 – 8.38 (m, 1H), 8.33 (s, 1H), 8.09 (d, *J* = 2.4 Hz, 1H), 7.81 (d, *J* = 8.0 Hz, 3H), 7.32 (d, *J* = 7.7 Hz, 2H), 2.45 (s, 3H); $^{13}$C NMR (101 MHz, $CDCl_3$): δ 188.74, 165.96, 143.30, 141.93, 134.41, 130.99, 129.66, 127.24, 126.26, 125.06, 124.12, 119.47, 119.41, 21.59; $^{19}$F NMR (376 MHz, $CDCl_3$): δ -54.86; HRMS (*m/z*): $[M+H]^+$ calcd. for $C_{16}H_{13}NO_2F_3$, 308.0898; found, 308.0899.

In a thick-walled vial, **S10** (200 mg, 0.65 mmol) was dissolved in ethanol (5 mL) and then **S2** (170 mg, 0.65 mmol) and piperidine (10 drops) were added at room temperature. The vial was sealed and heated at 80°C for 1 hour. The reaction mixture was cooled to room temperature and the solvent was evaporated. The crude material was purified by an ISCO combi flash silica gel column ($CH_2Cl_2$/MeOH to yield the tert-butyl benzoate. The tert-butyl ester (100 mg, 0.18 mmol) was treated with 2 mL $CH_2Cl_2$/TFA (1:1) to provide the crude product. The product was purified by a silica gel ISCO combi flash column ($CH_2Cl_2$/MeOH) to yield **XL32** (65 mg, 73%, single *E*-isomer). $^{1}$H NMR (400 MHz, DMSO-$d_6$): δ 12.35 (s, 1H), 10.76 (s, 1H), 8.67 – 8.60 (m, 2H), 8.57 (d, *J* = 2.1 Hz, 1H), 8.18 (dd, *J* = 8.7, 2.1 Hz, 1H), 8.07 (dd, *J* = 7.9, 1.7 Hz, 1H), 7.96 – 7.89 (m, 3H), 7.69 (ddd, *J* = 8.7, 7.4, 1.7 Hz, 1H), 7.37 (d, *J* = 7.9 Hz, 2H), 7.28 (td, *J* = 7.7, 1.2 Hz, 1H), 2.40 (s, 3H); $^{13}$C NMR (101 MHz, DMSO-$d_6$): δ 170.28, 166.50, 158.28, 150.00, 143.84, 142.79, 140.40, 134.81, 131.72, 131.69, 131.50, 129.46, 128.45, 128.06, 125.66, 124.56, 122.95, 122.48 – 121.82 (m), 120.88, 120.81, 117.56, 114.63, 112.55, 21.54; $^{19}$F NMR (376 MHz,

DMSO-$d_6$): δ -57.32; HRMS (m/z): $[M+H]^+$ calcd. for $C_{26}H_{19}N_3O_4F_3$, 494.1328; found, 494.1327.

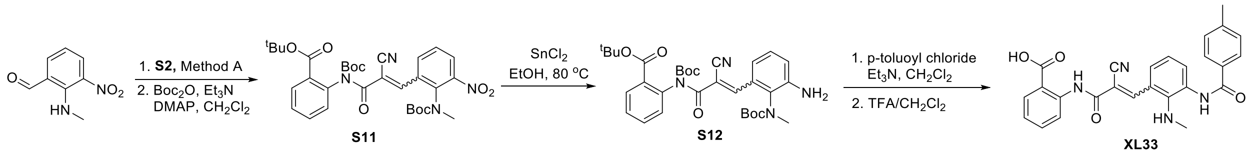


In a thick-walled vial, 2-(methylamino)-3-nitrobenzaldehyde (158 mg, 0.87 mmol) was dissolved in $CH_2Cl_2$ (3 mL) and then **S2** (228 mg, 0.87 mmol) and piperidine (10 drops) were added at room temperature. The vial was sealed and stirring was continued for 6 hours at room temperature. The solid product was collected, washed with $CH_2Cl_2$ (2 x 10 mL) to yield the tert-butyl benzoate. The tert-butyl benzoate (100 mg, 0.24 mmol) was dissolved in $CH_2Cl_2$ (5 mL) and then di-tert-butyl dicarbonate (400 mg, 1.83 mmol), $Et_3N$ (264 µLt, 1.83 mmol) and catalytic amounts of 4-dimethylaminopyridine were added at room temperature. The reaction mixture was stirred for 12 hours. Water (5 mL) was added to the reaction mixture and the product extracted with $CH_2Cl_2$ (2 x 6 mL) and dried ($Na_2SO_4$). After concentration, the crude product was purified by an ISCO combi flash silica gel column (EtOAc/hexanes) to yield **S11** (143 mg, 96%). Nitrobenzene **S11** (100 mg, 0.16 mmol) in ethanol (15 mL) was placed into a thick-walled vial and $SnCl_2$ (152 mg, 0.80 mmol) was added under an argon atmosphere at room temperature. The sealed vial was heated at 80°C for 1 hour, after which LC-MS indicated the complete consumption of starting material **S11**. The cooled reaction mixture was quenched with aqueous sodium bicarbonate (20 mL), product extracted with EtOAc (3 x 15 mL) and dried ($Na_2SO_4$). After concentration, the crude product was purified by an ISCO combi flash silica gel column (EtOAc/hexanes) to provide aniline derivative **S12** (73 mg, 77%, single *E*-isomer). $^1$H

NMR (400 MHz, $CDCl_3$): δ 8.00 (ddd, *J* = 7.8, 1.7, 0.4 Hz, 1H), 7.84 (s, 1H), 7.70 (ddd, *J* = 7.9, 1.3, 0.4 Hz, 1H), 7.57 (ddd, *J* = 7.9, 7.4, 1.6 Hz, 1H), 7.41 (ddd, *J* = 7.8, 7.4, 1.3 Hz, 1H), 7.04 (t, *J* = 7.7 Hz, 1H), 6.99 – 6.92 (m, 1H), 6.85 (dd, *J* = 7.7, 1.5 Hz, 1H), 3.73 (s, 3H), 1.56 (s, 18H), 1.23 (s, 9H); $^{13}$C NMR (101 MHz, $CDCl_3$): δ 167.74, 164.47, 159.29, 156.29, 151.64, 138.60, 137.42, 137.06, 132.57, 132.09, 130.91, 130.64, 130.56, 130.09, 128.07, 124.90, 123.95, 120.18, 119.70, 82.98, 81.34, 78.31, 42.22, 28.55, 28.21, 27.68; HRMS (m/z): $[M+H]^+$ calcd. for $C_{32}H_{41}N_4O_7$, 593.2975; found, 593.2980.

**XL33** was synthesized from **S12** using the same procedure as **XL5**-$^{13}C_6$-CB from **S4-**$^{13}C_6$-CB. Characterization data of **XL33**: $^1$H NMR (400 MHz, DMSO-$d_6$): δ 11.96 (s, 1H), 10.84 (s, 1H), 9.50 (d, *J* = 15.6 Hz, 2H), 9.04 (s, 1H), 8.31 (d, *J* = 8.2 Hz, 1H), 8.05 (dd, *J* = 7.9, 1.6 Hz, 1H), 8.00 (dd, *J* = 7.8, 1.5 Hz, 1H), 7.95 (d, *J* = 8.1 Hz, 2H), 7.88 (dd, *J* = 7.6, 1.5 Hz, 1H), 7.73 – 7.65 (m, 2H), 7.40 (d, *J* = 8.0 Hz, 2H), 7.33 (t, *J* = 7.7 Hz, 1H), 3.73 (s, 3H), 2.41 (s, 3H); $^{13}$C NMR (101 MHz, $CDCl_3$): δ 169.66, 165.87, 163.26, 155.14, 143.29, 143.04, 139.38, 135.92, 134.82, 134.29, 131.59, 130.74, 129.69, 129.34, 128.42, 128.14, 126.83, 125.15, 123.51, 122.51, 120.29, 119.37, 41.12, 21.53; HRMS (m/z): $[M+H]^+$ calcd. for $C_{26}H_{23}N_4O_4$, 455.1719; found, 455.1727.

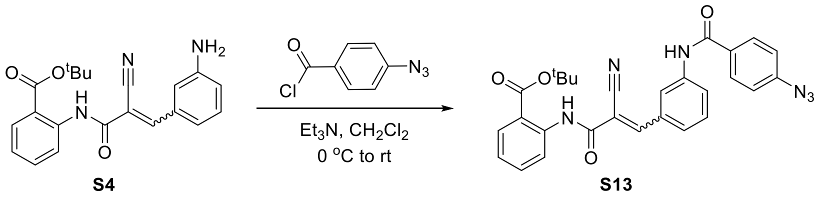


In a 25 mL round bottom flask, **S4** (300 mg, 0.82 mmol) was dissolved in $CH_2Cl_2$ (6 mL) and cooled to 0°C. The freshly prepared 4-azidobenzoyl chloride (167 mg, 0.91 mmol)

and triethylamine (0.24 mL, 1.64 mmol) were added to the cooled solution. The reaction mixture was warmed to room temperature and stirred for 5 hours. Water (5 mL) was added to the reaction mixture and product extracted with $CH_2Cl_2$ (2 x 10 mL). The combined layers were dried over anhydrous $Na_2SO_4$, filtered, and concentrated to provide crude product. The crude material was purified by a silica gel ISCO combi flash column (hexanes/EtOAc) to yield **S13** (390 mg, 93%). $^1$H NMR (400 MHz, $CDCl_3$): δ 12.16 (s, 1H), 8.84 (s, 1H), 8.68 (dd, *J* = 8.4, 1.1 Hz, 1H), 8.27 – 8.16 (m, 2H), 7.98 (dd, *J* = 8.0, 1.6 Hz, 1H), 7.94 (ddd, *J* = 8.2, 2.2, 0.9 Hz, 1H), 7.90 – 7.82 (m, 2H), 7.74 – 7.61 (m, 1H), 7.50 (ddd, *J* = 8.7, 7.3, 1.7 Hz, 1H), 7.40 (t, *J* = 8.0 Hz, 1H), 7.11 (ddd, *J* = 8.2, 7.4, 1.2 Hz, 1H), 6.97 – 6.86 (m, 2H), 1.58 (s, 9H); $^{13}$C NMR (101 MHz, $CDCl_3$): δ 167.48, 165.34, 159.21, 153.05, 143.71, 140.48, 139.15, 133.93, 132.45, 131.08, 130.84, 129.76, 129.29, 126.67, 124.95, 123.49, 122.20, 120.87, 118.92, 117.71, 115.86, 106.06, 82.94, 28.10; HRMS (m/z): $[M+Na]^+$ calcd. for $C_{28}H_{24}N_6O_4Na$, 531.1757; found, 531.1760.

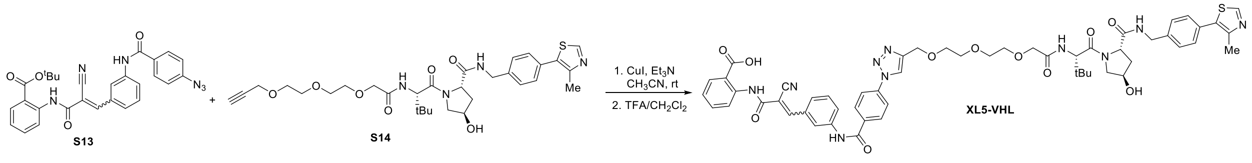


Azide **S13** (8 mg, 0.016 mmol), alkyne **S14** (10 mg, 0.016 mmol) and dry $CH_3CN$ (3 ml) were placed into a round bottom flask equipped with argon, and then CuI (0.3 mg, 0.0016 mmol) and $Et_3N$ (2.3 μL, 0.016 mmol) were added to the reaction mixture. The reaction mixture was stirred overnight at room temperature, then acetonitrile was removed under reduced pressure. The crude material was purified by a silica gel ISCO combi flash column ($CH_2Cl_2$ /MeOH) to yield the click product. The click product was subjected to 1 mL $CH_2Cl_2$/TFA (1:1) and stirred for 1 hour (monitored by LC-MS). The solvent was

removed and purified by a silica gel ISCO combi flash column ($CH_2Cl_2$ /MeOH) to provide **XL5-VHL** (11 mg, 65% over two-steps). $^1$H NMR (400 MHz, DMSO-$d_6$): δ 10.69 (s, 1H), 8.99 (s, 1H), 8.92 (s, 1H), 8.59 (q, $J$ = 7.6, 6.8 Hz, 2H), 8.46 (s, 1H), 8.32 (s, 1H), 8.24 – 8.16 (m, 2H), 8.16 – 8.02 (m, 3H), 7.98 (dd, $J$ = 7.8, 1.9 Hz, 1H), 7.85 – 7.77 (m, 1H), 7.60 (t, $J$ = 7.9 Hz, 1H), 7.51 – 7.31 (m, 6H), 7.12 (s, 1H), 5.17 (s, 1H), 4.63 (s, 2H), 4.60 – 4.52 (m, 1H), 4.50 – 4.30 (m, 3H), 4.30 – 4.15 (m, 1H), 3.97 (s, 2H), 3.72 – 3.51 (m, 10H), 2.42 (s, 3H), 2.14 – 2.01 (m, 1H), 1.90 (ddd, $J$ = 12.9, 8.8, 4.5 Hz, 1H), 0.93 (s, 9H); $^{13}$C NMR (101 MHz, DMSO-$d_6$): δ 172.23, 169.60, 169.10, 167.66, 165.13, 159.47, 151.90, 151.71, 148.18, 145.89, 140.78, 140.19, 139.85, 139.24, 136.67, 134.66, 132.79, 131.67, 130.14, 130.11, 130.05, 129.32, 129.13, 127.91, 125.98, 124.99, 123.31, 122.74, 122.48, 120.06, 120.01, 119.82, 116.12, 108.18, 70.88, 70.20, 70.08, 69.65, 69.31, 63.85, 59.21, 57.01, 56.17, 42.13, 40.39, 38.36, 36.14, 26.61, 16.35; HRMS (m/z): $[M+H]^+$ calcd. for $C_{55}H_{59}N_{10}O_{11}S$, 1067.4085; found, 1067.4063.

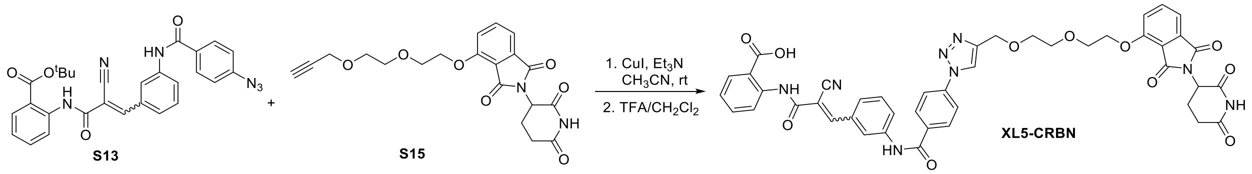


**XL5-CRBN** was synthesized using the same reaction sequence as **XL5-VHL**. Starting materials azide **S13** (11 mg, 0.022 mmol), alkyne **S14** (10 mg, 0.022 mmol), CuI (0.4 mg, 0.0022 mmol), and $Et_3N$ (3 μL, 0.022 mmol) were used. The crude product was purified by a silica gel ISCO combi flash column ($CH_2Cl_2$ /MeOH) to provide **XL5-CRBN** (12 mg, 63%, *E/Z* = 5 : 1). Characterization data of major isomer: $^1$H NMR (400 MHz, DMSO-$d_6$): δ 12.23 (s, 1H), 11.09 (s, 1H), 10.66 (s, 1H), 8.92 (s, 1H), 8.67 – 8.61 (m, 1H), 8.50 (t, $J$ = 2.0 Hz, 1H), 8.41 (s, 1H), 8.19 (d, $J$ = 8.7 Hz, 2H), 8.11 (d, $J$ = 8.7 Hz, 2H), 8.06 (dd, $J$

= 8.0, 1.7 Hz, 1H), 8.02 – 7.95 (m, 2H), 7.85 – 7.76 (m, 2H), 7.72 – 7.66 (m, 1H), 7.61 (t, *J* = 7.9 Hz, 1H), 7.52 (d, *J* = 8.7 Hz, 1H), 7.43 (d, *J* = 7.2 Hz, 1H), 7.32 – 7.20 (m, 1H), 5.07 (dd, *J* = 12.8, 5.3 Hz, 1H), 4.65 (s, 2H), 4.35 (t, *J* = 4.6 Hz, 2H), 3.82 (t, *J* = 4.6 Hz, 2H), 3.75 – 3.65 (m, 4H), 2.99 – 2.77 (m, 1H), 2.68 – 2.40 (m, 2H), 2.05 – 1.92 (m, 1H); $^{13}$C NMR (101 MHz, DMSO-$d_6$): δ 173.23, 173.13, 170.40, 167.25, 165.74, 165.12, 159.46, 156.27, 153.26, 145.94, 140.64, 140.49, 140.26, 139.26, 137.42, 134.83, 134.65, 133.70, 132.61, 131.72, 130.19, 130.04, 125.42, 124.30, 122.75, 122.49, 120.87, 120.47, 120.06, 117.47, 116.78, 115.99, 115.83, 106.65, 70.57, 69.74, 69.35, 63.90, 55.37, 49.25, 31.40, 22.43; HRMS (m/z): $[M+H]^+$ calcd. for $C_{44}H_{37}N_8O_{11}$, 853.2582; found, 853.2576.

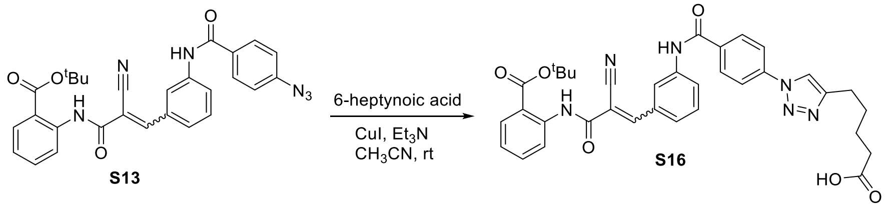


Azide **S13** (50 mg, 0.098 mmol), 6-heptynoic acid (13 mg, 0.098 mmol) and dry $CH_3CN$ (3 ml) were placed into a round bottom flask equipped with argon, and then CuI (2 mg, 0.0098 mmol) and $Et_3N$ (15 µL, 0.098 mmol) were added to the reaction mixture. The reaction mixture was stirred overnight at room temperature, then acetonitrile was removed under reduced pressure. The crude material was purified by a silica gel ISCO combi flash column ($CH_2Cl_2$ /MeOH) to yield **S16** (43 mg, 69%). $^1$H NMR (400 MHz, DMSO-$d_6$): δ 12.01 (s, 1H), 11.58 (s, 1H), 10.64 (s, 1H), 8.71 (d, *J* = 0.7 Hz, 1H), 8.52 (t, *J* = 1.9 Hz, 1H), 8.41 – 8.31 (m, 2H), 8.24 – 8.15 (m, 2H), 8.11 – 8.00 (m, 2H), 8.00 – 7.89 (m, 2H), 7.79 (ddd, *J* = 8.3, 1.7, 0.8 Hz, 1H), 7.65 (ddd, *J* = 8.4, 7.4, 1.7 Hz, 1H), 7.60 (t, *J* = 8.0 Hz, 1H), 7.27 (ddd, *J* = 7.9, 7.3, 1.2 Hz, 1H), 2.72 (t, *J* = 7.4 Hz, 2H), 2.26

(t, $J$ = 7.2 Hz, 2H), 1.76 – 1.64 (m, 2H), 1.63 – 1.57 (m, 2H), 1.56 (s, 4H); $^{13}C$ NMR (101 MHz, DMSO-$d_6$): δ 174.85, 167.14, 165.13, 159.76, 152.99, 148.70, 140.28, 139.46, 139.42, 134.35, 134.27, 132.59, 131.29, 130.17, 130.04, 126.28, 125.28, 124.69, 122.34, 122.06, 120.69, 120.03, 119.74, 116.04, 106.76, 82.94, 33.85, 28.65, 28.17, 25.19, 24.52; HRMS (m/z): $[M+H]^+$ calcd. for $C_{35}H_{35}N_6O_6$, 635.2618; found, 635.2628.

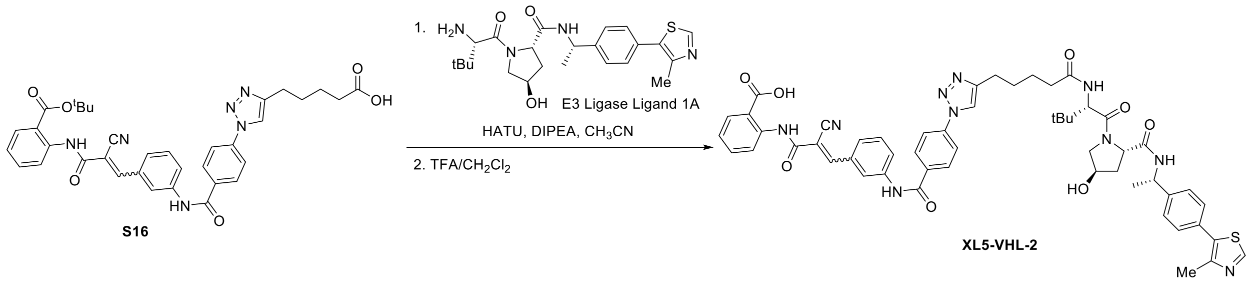


In a 10 mL round bottom flask, **S16** (50 mg, 0.079 mmol) was dissolved in $CH_3CN$ (3 ml), and then HATU (36 mg, 0.094 mmol), DIPEA (44 µL, 0.24 mmol), and HCl salt of E3 ligase ligand 1A (38 mg, 0.079 mmol) were added at room temperature. The reaction mixture was stirred for 45 min (monitored by LC-MS), the solvent was removed under the reduced pressure and purified by a silica gel ISCO combi flash column ($CH_2Cl_2$ /MeOH) to provide tBu-XL5-VHL-2. The product was subjected to 1 mL $CH_2Cl_2$/TFA (1:1) and stirred for 1 hour at room temperature. The solvent and TFA were removed under the reduced pressure to provide the **XL5-VHL-2** crude material. The crude material was purified by a preparatory HPLC with a XBridge BEH C18 OBD Prep Column, 130Å, 5 µm, 30 mm X 150 mm reverse-phase column as the stationary phase. Water (buffered with 0.05% trifluoroacetic acid) and MeCN were used as the mobile phase and HPLC conditions: UV collection 254 nm, flow rate 30 mL/min, 20% MeCN as linear gradient for 5 min and 20% → 70% MeCN for 5 to 25 min. The HPLC fractions were combined and

lyophilized to yield **XL5-VHL-2** (26 mg, 33%, *E/Z* = 3.5:1). $^1$H NMR (400 MHz, DMSO-$d_6$): δ 12.24 (s, 1H), 10.67 (s, 1H), 8.98 (s, 1H), 8.71 (s, 1H), 8.64 (dd, *J* = 8.5, 1.1 Hz, 1H), 8.51 (t, *J* = 1.9 Hz, 1H), 8.41 (s, 1H), 8.37 (d, *J* = 7.8 Hz, 1H), 8.20 (d, *J* = 8.8 Hz, 2H), 8.14 – 8.02 (m, 3H), 8.00 – 7.95 (m, 1H), 7.87 – 7.78 (m, 2H), 7.70 (ddd, *J* = 8.7, 7.3, 1.7 Hz, 1H), 7.62 (t, *J* = 8.0 Hz, 1H), 7.43 (d, *J* = 8.4 Hz, 2H), 7.37 (d, *J* = 8.3 Hz, 2H), 7.27 (td, *J* = 7.6, 1.2 Hz, 1H), 4.91 (t, *J* = 7.2 Hz, 1H), 4.52 (d, *J* = 9.3 Hz, 1H), 4.42 (t, *J* = 8.0 Hz, 1H), 4.34 – 4.22 (m, 1H), 3.63 – 3.60 (m, 2H), 2.74 (t, *J* = 7.2 Hz, 2H), 2.45 (s, 3H), 2.38 – 2.14 (m, 2H), 2.05 – 1.94 (m, 1H), 1.79 (ddd, *J* = 12.7, 8.5, 4.5 Hz, 1H), 1.74 – 1.50 (m, 4H), 1.36 (d, *J* = 6.9 Hz, 3H), 0.93 (s, 9H); $^{13}$C NMR (101 MHz, DMSO-$d_6$): δ 172.40, 171.08, 170.24, 170.08, 165.17, 159.47, 153.27, 151.99, 151.93, 148.84, 148.18, 145.12, 140.64, 140.26, 139.44, 134.84, 134.79, 134.37, 132.61, 131.73, 131.66, 131.59, 130.13, 130.06, 129.28, 126.84, 124.33, 122.51, 120.88, 120.69, 119.77, 117.50, 116.00, 106.65, 69.23, 59.01, 56.90, 48.21, 48.09, 38.18, 35.64, 35.06, 28.82, 26.92, 25.45, 25.20, 22.89, 16.39; HRMS (m/z): $[M+H]^+$ calcd. for $C_{54}H_{57}N_{10}O_8S$, 1005.4082; found, 1005.4093.

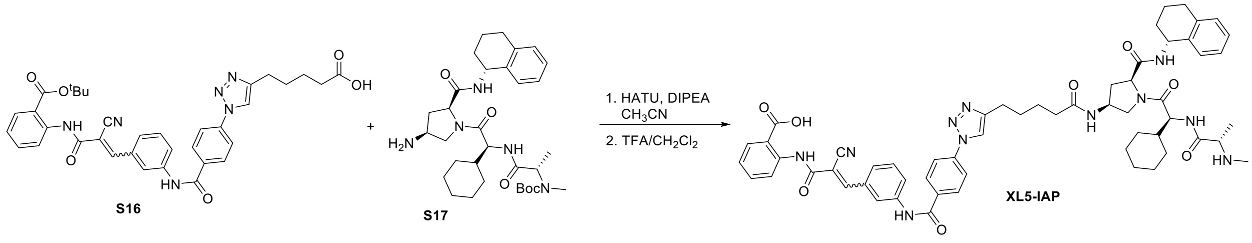


**XL5-IAP** was synthesized using the same reaction sequence as **XL5-VHL-2**. Starting materials **S16** (50 mg, 0.079 mmol), HATU (35 mg, 0.094 mmol), DIPEA (44 µL, 0.24 mmol), and HCl salt of **S17** (50 mg, 0.079 mmol) were used. The crude material was purified by a preparatory HPLC with a XBridge BEH C18 OBD Prep Column, 130Å, 5 µm, 30 mm X 150 mm reversed-phase column as the stationary phase. Water (buffered with

0.05% trifluoroacetic acid) and MeCN were used as the mobile phase and HPLC conditions: UV collection 254 nm, flow rate 30 mL/min, 40% MeCN as linear gradient for 5 min and 40% → 55% MeCN for 5 to 20 min. The HPLC fractions of major isomer were combined and lyophilized to yield **XL5-IAP** (22 mg, 28%). $^1$H NMR (400 MHz, DMSO-$d_6$): δ 13.09 (s, 1H), 10.69 (s, 1H), 8.80 – 8.67 (m, 2H), 8.61 (d, *J* = 8.3 Hz, 1H), 8.48 (d, *J* = 2.0 Hz, 1H), 8.41 (d, *J* = 8.7 Hz, 1H), 8.36 (s, 1H), 8.24 – 8.17 (m, 2H), 8.12 – 8.03 (m, 2H), 7.98 (dd, *J* = 7.9, 2.0 Hz, 1H), 7.81 (d, *J* = 7.9 Hz, 1H), 7.59 (t, *J* = 7.9 Hz, 2H), 7.28 (d, *J* = 7.5 Hz, 1H), 7.21 (t, *J* = 7.6 Hz, 1H), 7.17 – 7.03 (m, 3H), 4.91 (t, *J* = 6.6 Hz, 1H), 4.48 – 4.15 (m, 3H), 4.11 (t, *J* = 8.6 Hz, 1H), 3.86 (t, *J* = 6.8 Hz, 1H), 3.33 (t, *J* = 8.8 Hz, 1H), 2.85 – 2.58 (m, 4H), 2.38 (dt, *J* = 12.6, 7.6 Hz, 1H), 2.14 (t, *J* = 7.0 Hz, 2H), 1.97 – 1.40 (m, 16H), 1.43 – 0.72 (m, 9H); $^{13}$C NMR (101 MHz, DMSO-$d_6$): δ 172.37, 171.23, 170.44, 169.75, 169.11, 165.11, 159.43, 152.69, 148.73, 140.66, 140.26, 139.42, 137.73, 137.43, 134.32, 133.80, 132.64, 131.72, 130.07, 129.01, 128.83, 127.12, 126.09, 125.28, 123.97, 122.57, 120.67, 120.46, 119.68, 116.04, 107.13, 58.89, 56.22, 55.94, 52.85, 48.16, 47.22, 35.61, 34.83, 31.26, 31.17, 30.24, 29.19, 28.92, 28.75, 28.59, 26.18, 25.96, 25.23, 25.09, 20.63, 16.20; HRMS (m/z): $[M+H]^+$ calcd. for $C_{58}H_{66}N_{11}O_8$, 1044.5096; found, 1044.5090.

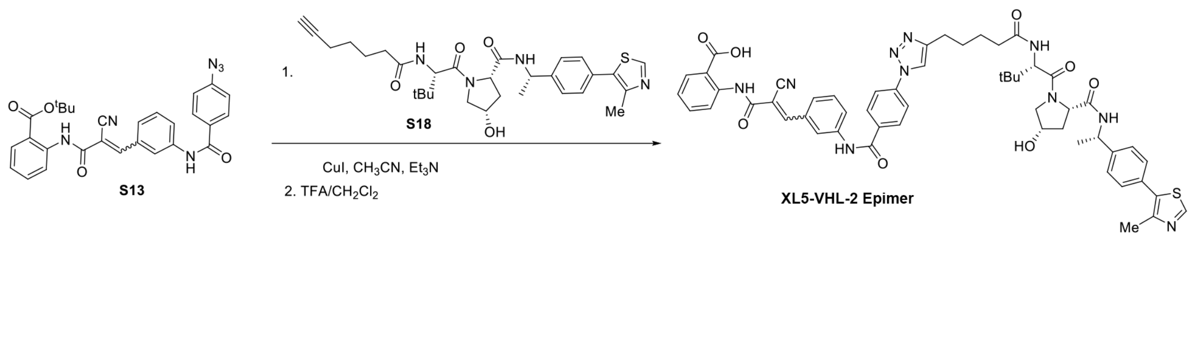

**XL5-VHL-2** Epimer was synthesized using the same reaction sequence as **XL5-VHL**. Starting materials azide **S13** (20 mg, 0.04 mmol), alkyne **S18** (22 mg, 0.04 mmol), CuI (1.5 mg, 0.008 mmol), and $Et_3N$ (6 μL, 0.04 mmol) were used. The final crude **XL5-VHL-2** Epimer was purified by an ISCO combi flash C18 reverse-phase column as the stationary phase. Water (buffered with 0.05% trifluoroacetic acid) and MeCN were used as the mobile phase. The fractions were combined and lyophilized to yield **XL5-VHL-2** Epimer (16 mg, 40%, over two steps). $^1H$ NMR (400 MHz, DMSO-$d_6$): δ 12.23 (s, 1H), 10.65 (s, 1H), 8.97 (s, 1H), 8.70 (d, *J* = 1.4 Hz, 1H), 8.62 (d, *J* = 8.4 Hz, 1H), 8.49 (d, *J* = 2.0 Hz, 1H), 8.39 (s, 1H), 8.29 (d, *J* = 7.7 Hz, 1H), 8.19 (dd, *J* = 8.8, 1.6 Hz, 2H), 8.10 – 8.03 (m, 3H), 7.96 (d, *J* = 8.1 Hz, 1H), 7.82 (t, *J* = 8.9 Hz, 2H), 7.68 (t, *J* = 7.9 Hz, 1H), 7.60 (td, *J* = 7.9, 1.3 Hz, 1H), 7.43 – 7.35 (m, 4H), 7.25 (t, *J* = 7.5 Hz, 1H), 4.91 (t, *J* = 7.2 Hz, 1H), 4.43 (d, *J* = 8.7 Hz, 1H), 4.31 (t, *J* = 7.5 Hz, 1H), 4.18 (t, *J* = 5.6 Hz, 1H), 3.89 (dd, *J* = 10.2, 5.6 Hz, 1H), 3.37 (dd, *J* = 10.1, 5.2 Hz, 1H), 2.72 (t, *J* = 7.3 Hz, 2H), 2.43 (s, 3H), 2.34 – 2.14 (m, 3H), 1.70 – 1.51 (m, 5H), 1.35 (d, *J* = 1.4 Hz, 3H), 0.94 (s, 9H). $^{13}C$ NMR (101 MHz, DMSO-$d_6$): δ 172.71, 171.52, 170.41, 170.22, 165.14, 159.45, 153.25, 151.99, 148.82, 148.18, 144.75, 140.63, 140.25, 139.42, 134.79, 134.35, 132.59, 131.68, 130.20, 130.16, 130.05, 129.28, 126.85, 126.34, 125.37, 124.29, 122.50, 120.86, 120.67, 119.74, 117.48, 115.98, 106.64, 69.43, 58.93, 57.23, 55.94, 48.23, 37.25, 35.06, 34.92, 28.77, 26.89, 25.41, 25.16, 22.68, 16.39. HRMS (m/z): $[M+H]^+$ calcd. for $C_{54}H_{57}N_{10}O_8S$, 1005.4082; found, 1005.4072.

## S1-$^{13}C_6$ $^{1}H$ NMR in $CDCl_3$

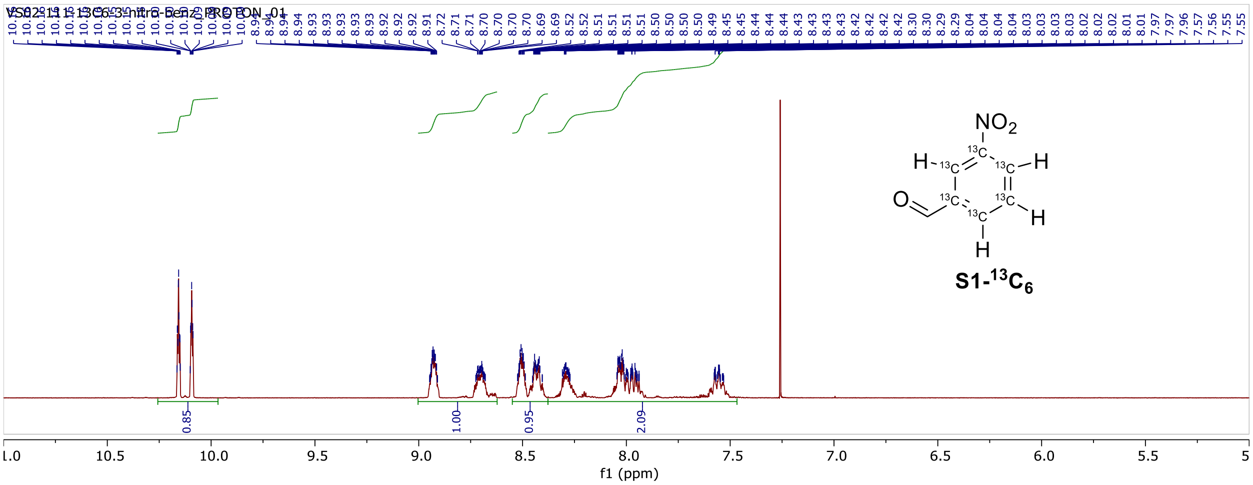


## S1-$^{13}C_6$ BilevelDec $^{1}H$ NMR in $CDCl_3$

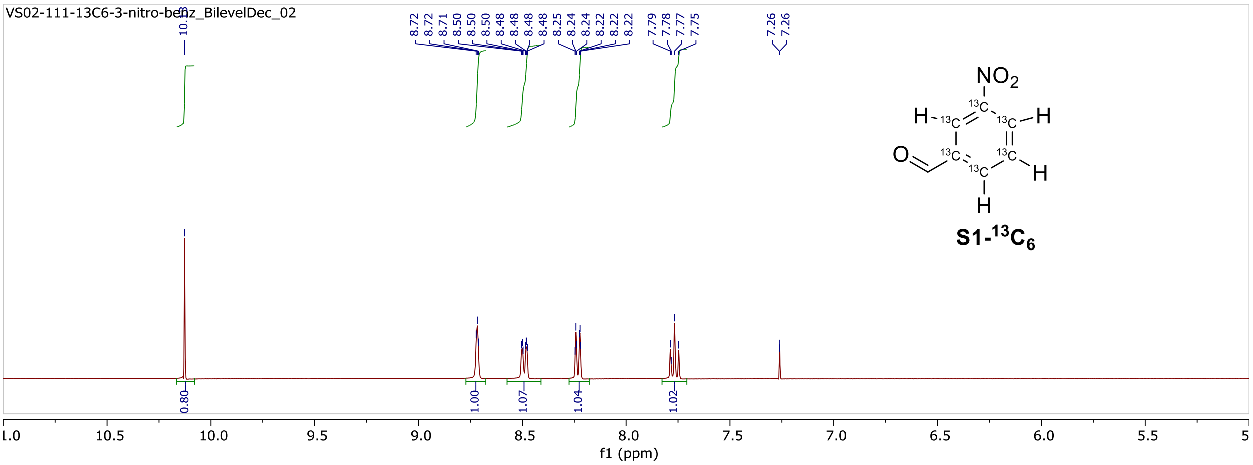


## S1-$^{13}C_6$ $^{13}C$ NMR in $CDCl_3$

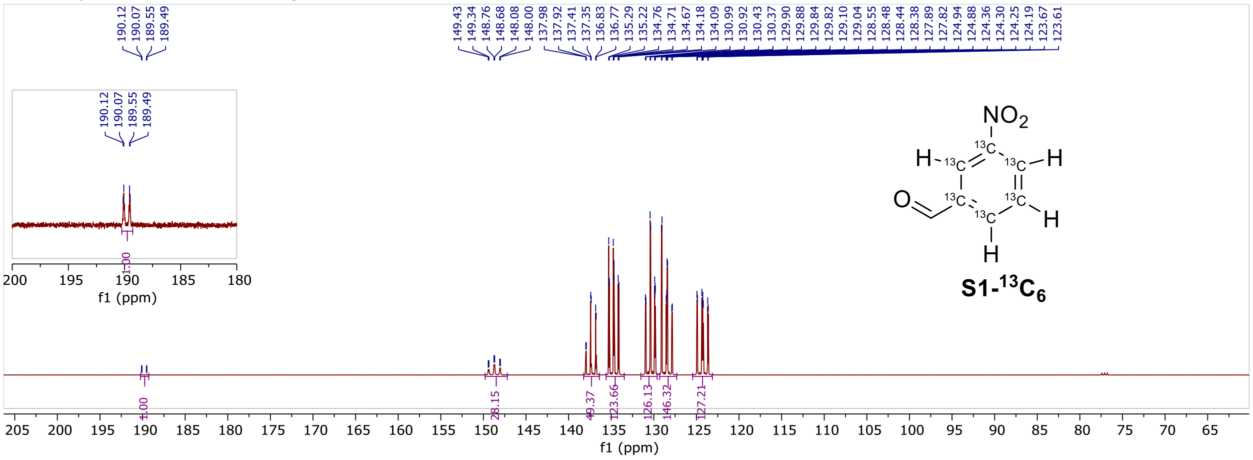

**S3-$^{13}C_6$-CB** $^{1}$H NMR in $CDCl_3$

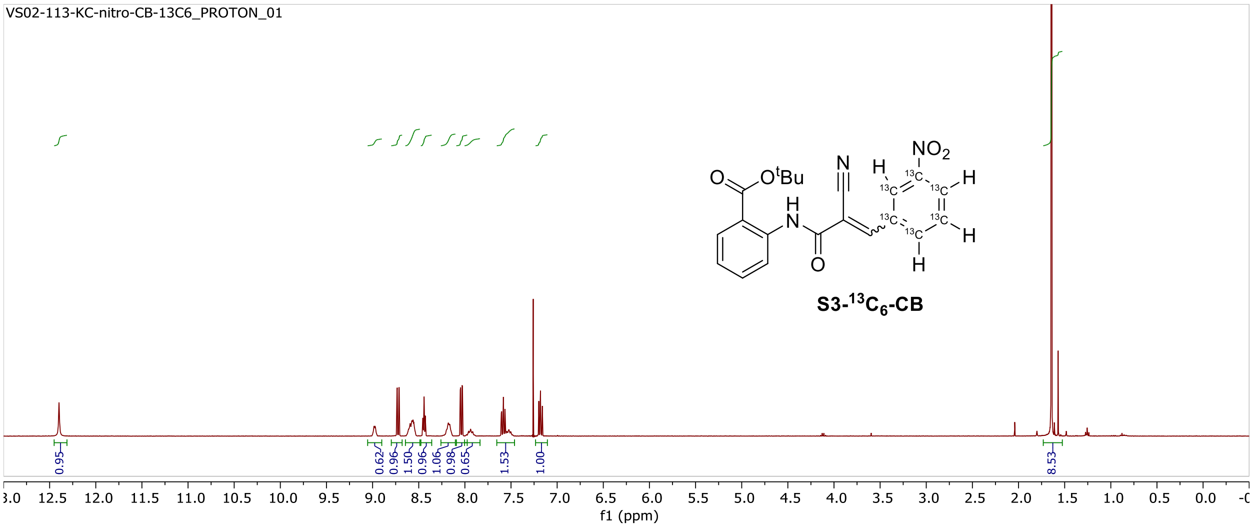


**S3-$^{13}C_6$-CB** BilevelDec $^{1}$H NMR in $CDCl_3$

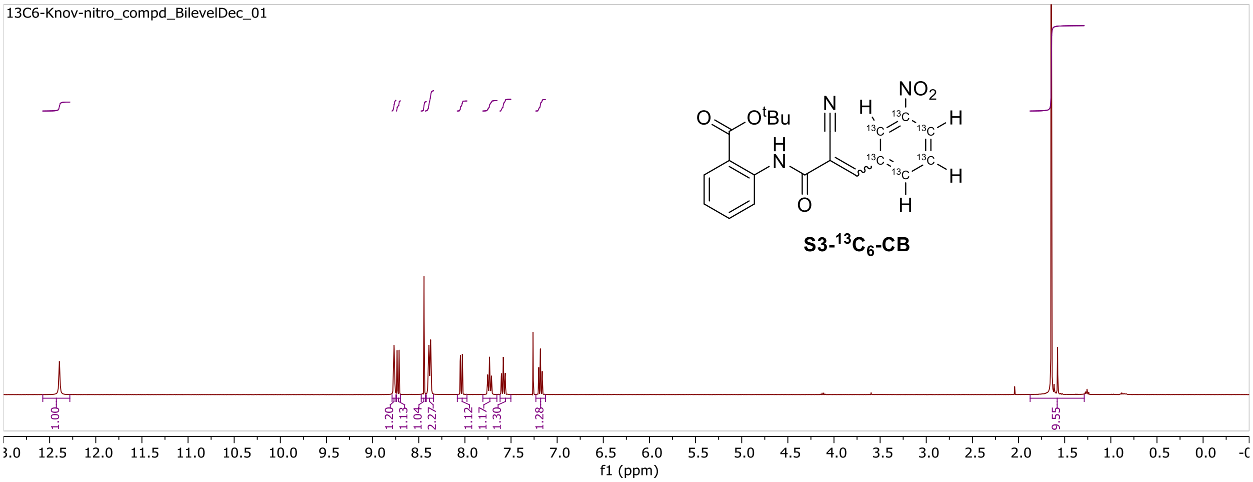


**S3-$^{13}C_6$-CB** $^{13}$C NMR in $CDCl_3$

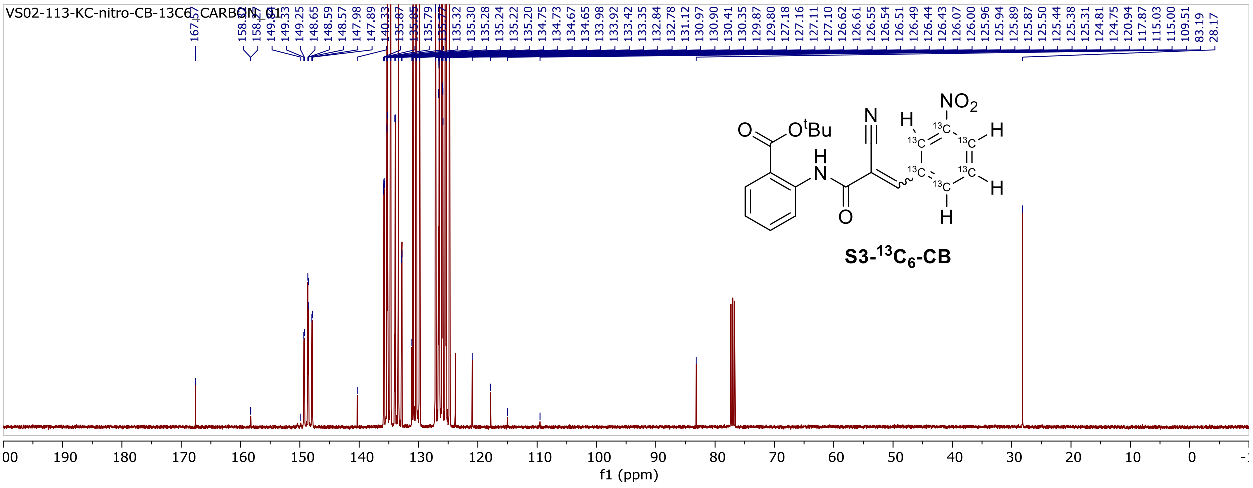

**S4-$^{13}C_6$-CB** $^1$H NMR in $CDCl_3$

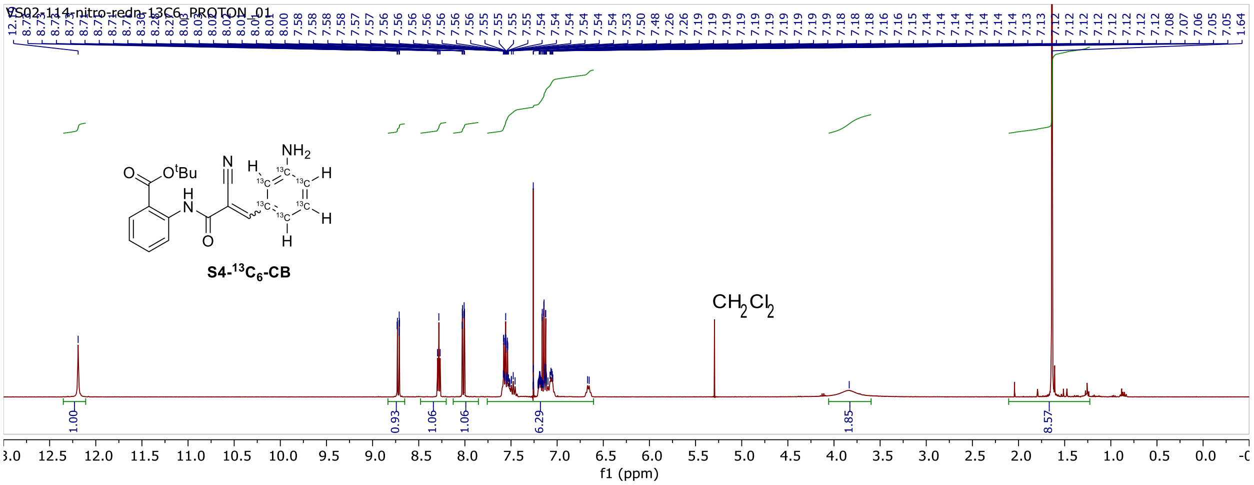


**S4-$^{13}C_6$-CB** BilevelDec $^1$H NMR in $CDCl_3$

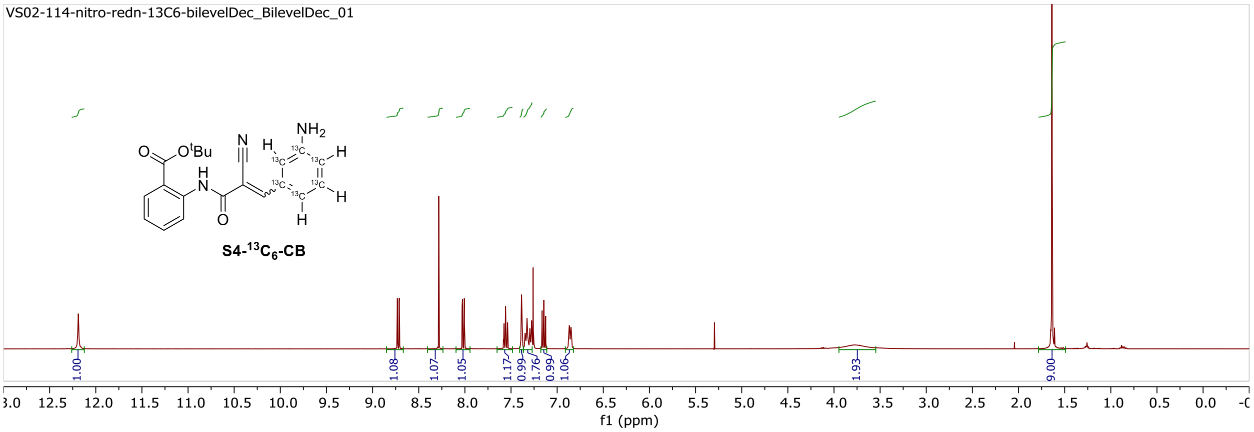


**S4-$^{13}C_6$-CB** $^{13}$C NMR in $CDCl_3$

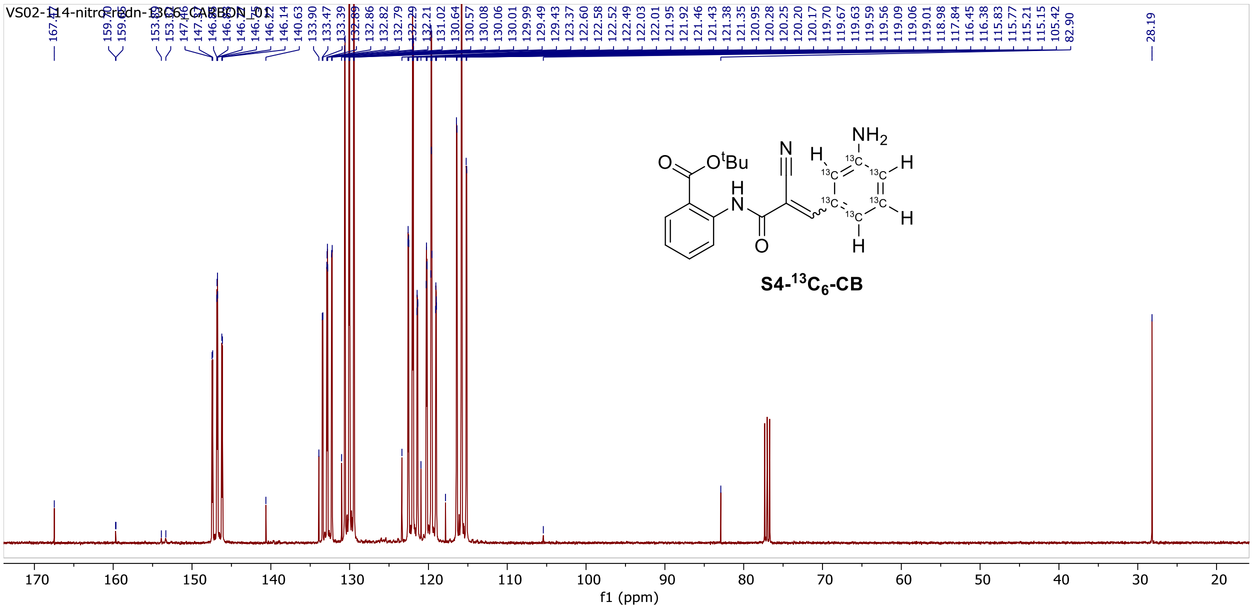

## **XL5-$^{13}$C$_6$-CB** $^1$H NMR in $CDCl_3$

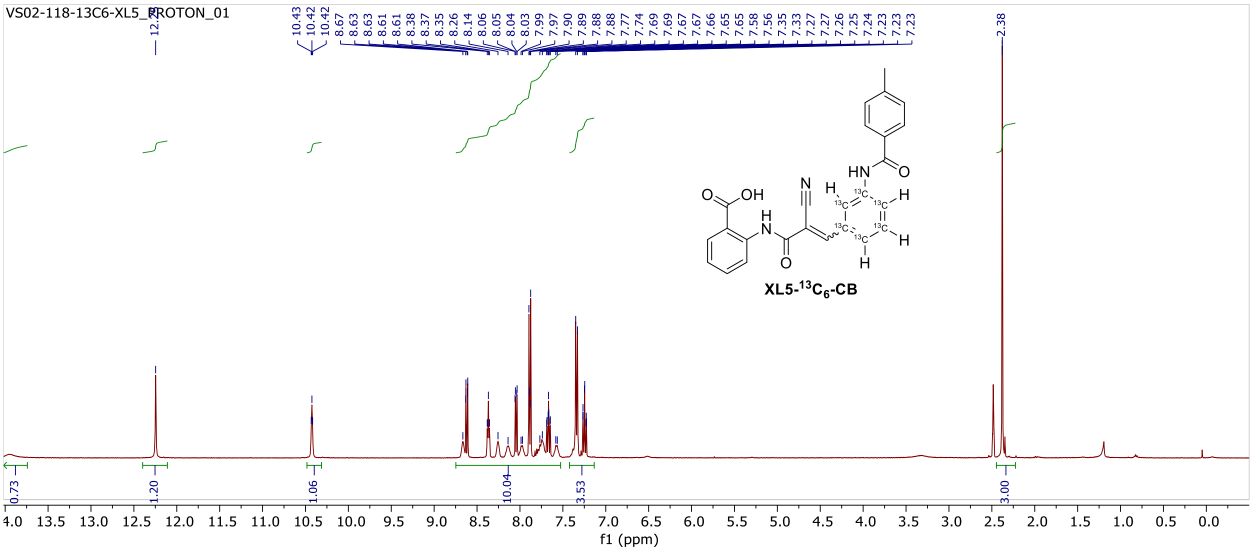


## **XL5-$^{13}$C$_6$-CB** BilevelDec $^1$H NMR in $CDCl_3$

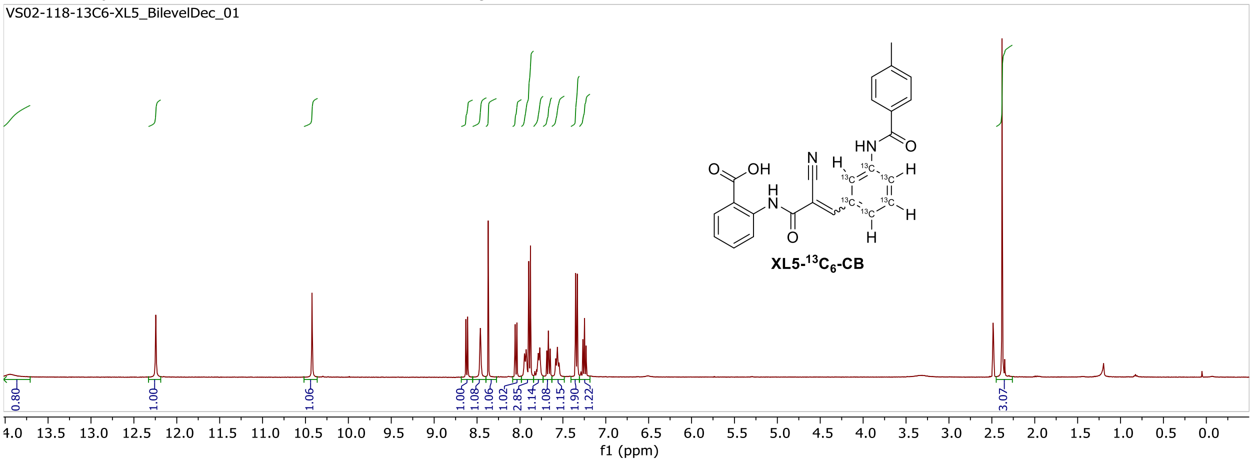


## **XL5-$^{13}$C$_6$-CB** $^{13}$C NMR in $CDCl_3$

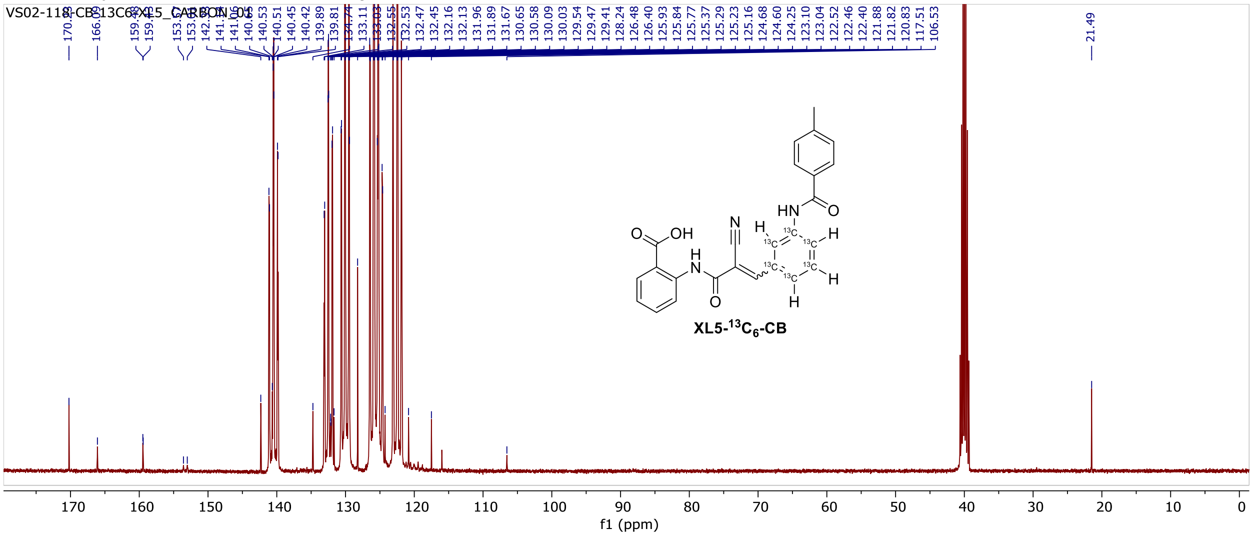

**XL25** $^{1}$H NMR in DMSO-*d*$_6$

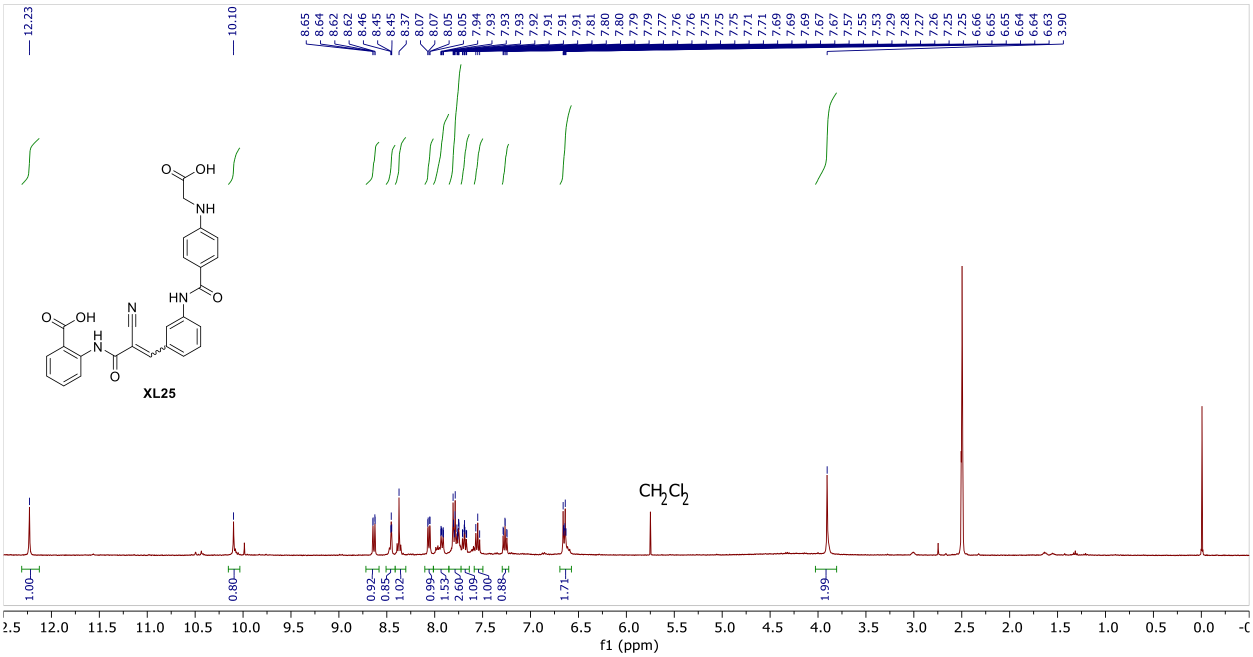

**XL25** $^{13}$C NMR in DMSO-*d*$_6$

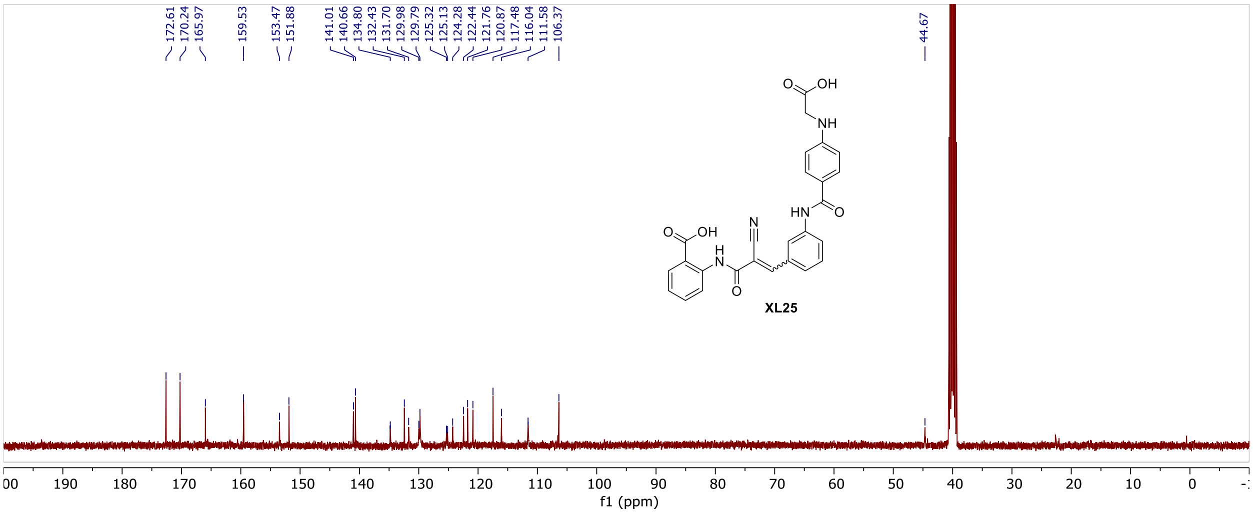

**XL26** $^{1}H$ NMR in DMSO-$d_6$

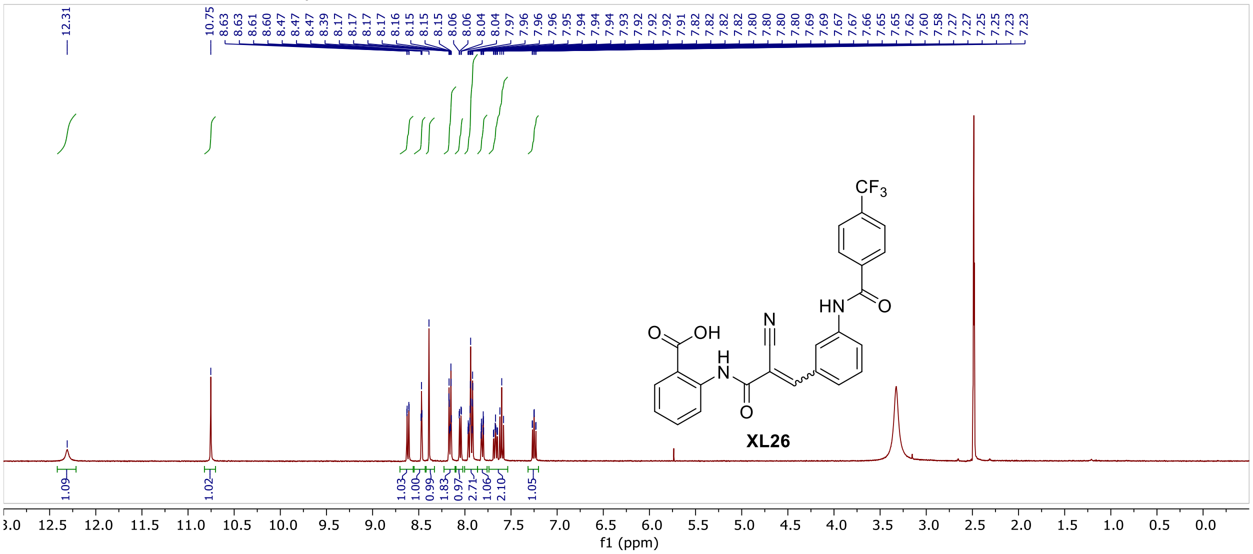

**XL26** $^{13}C$ NMR in DMSO-$d_6$

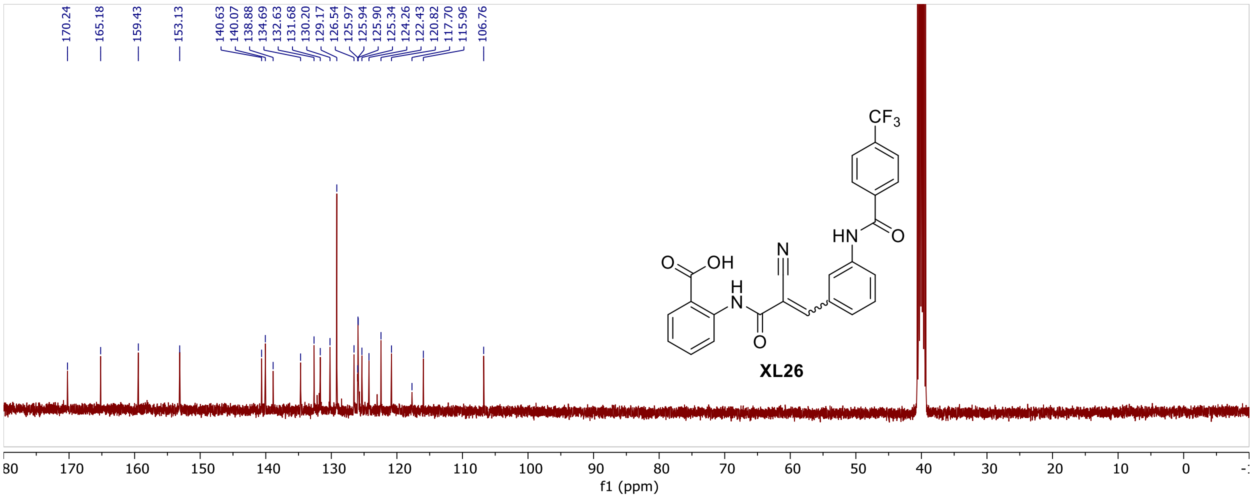

**XL26** $^{19}F$ NMR in DMSO-$d_6$

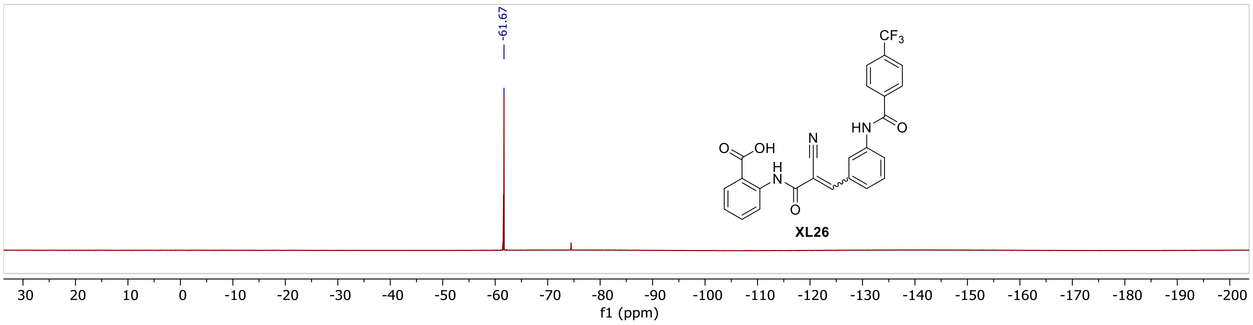

**XL27** $^{1}$H NMR in DMSO-$d_6$

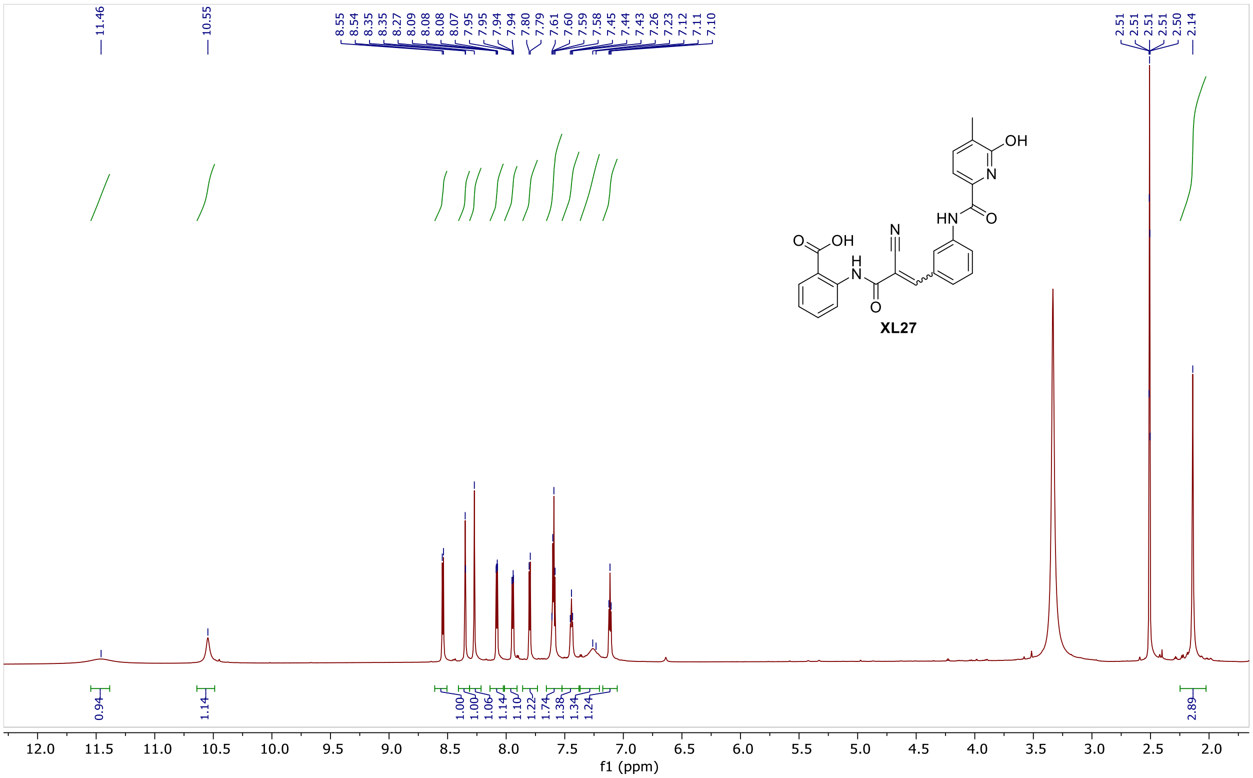

**XL27** $^{13}$C NMR in DMSO-$d_6$

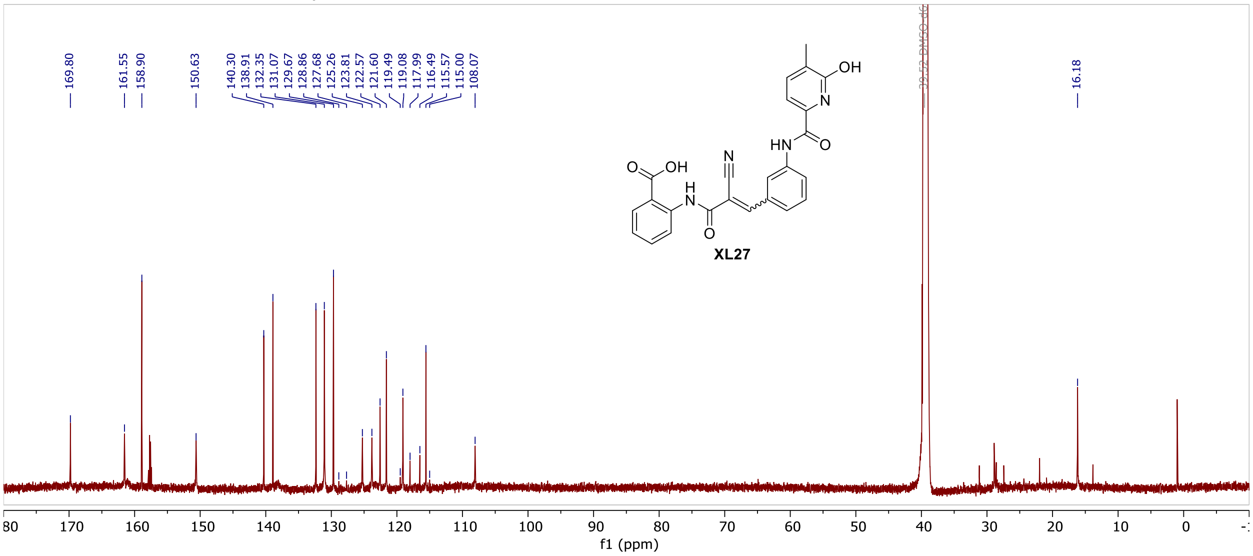

**XL30** ¹H NMR in DMSO-$d_6$

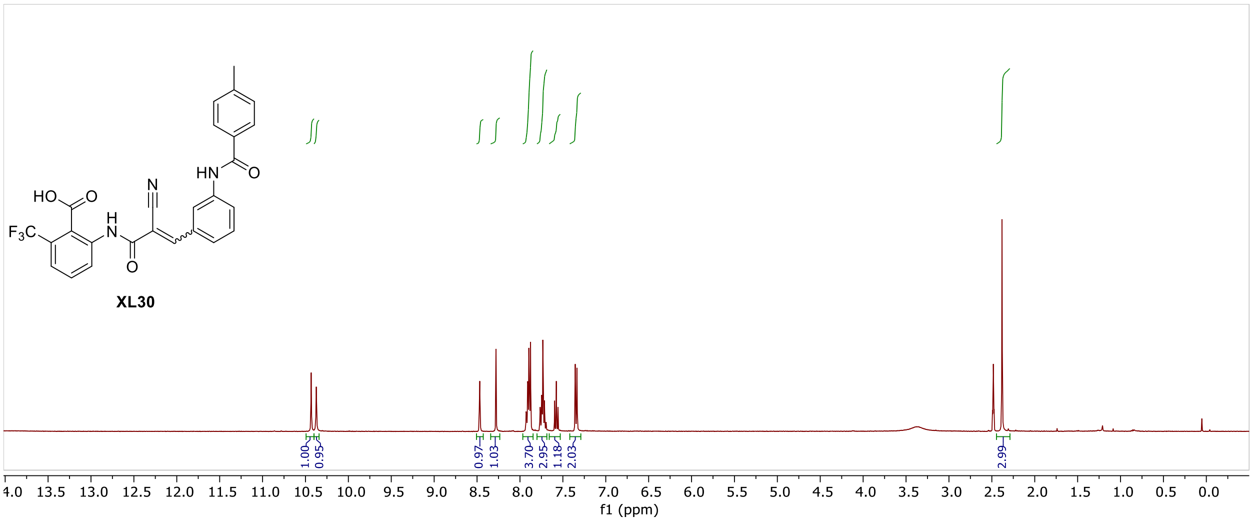


**XL30** ¹³C NMR in DMSO-$d_6$

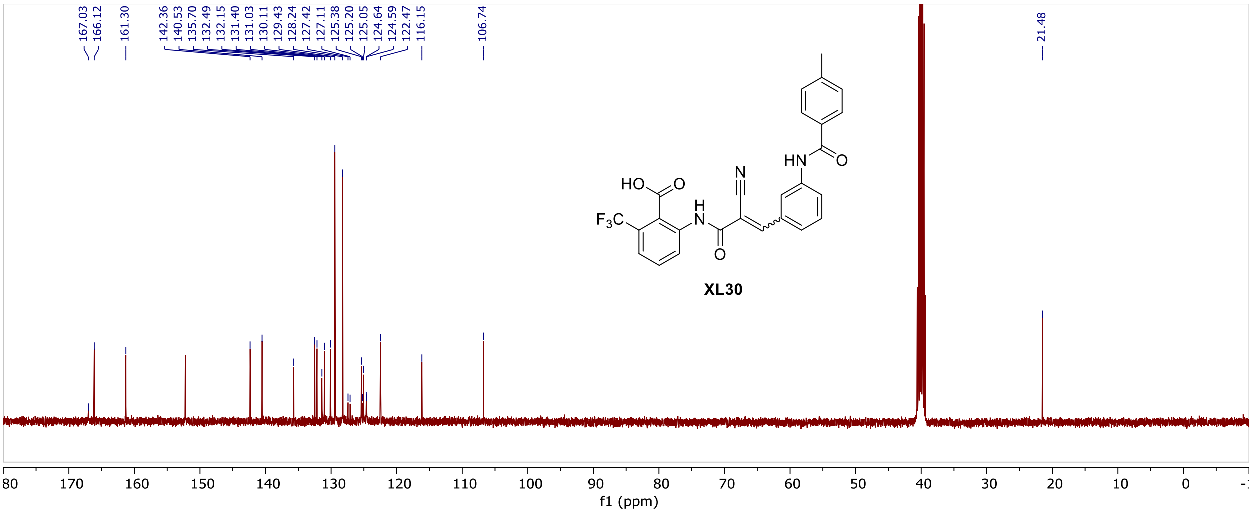


**XL30** ¹⁹F NMR in DMSO-$d_6$

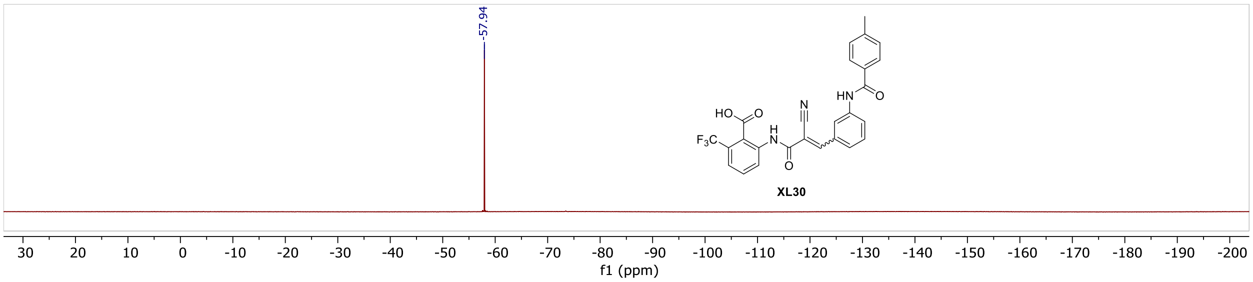

**XL31** $^{1}$H NMR in DMSO-$d_6$

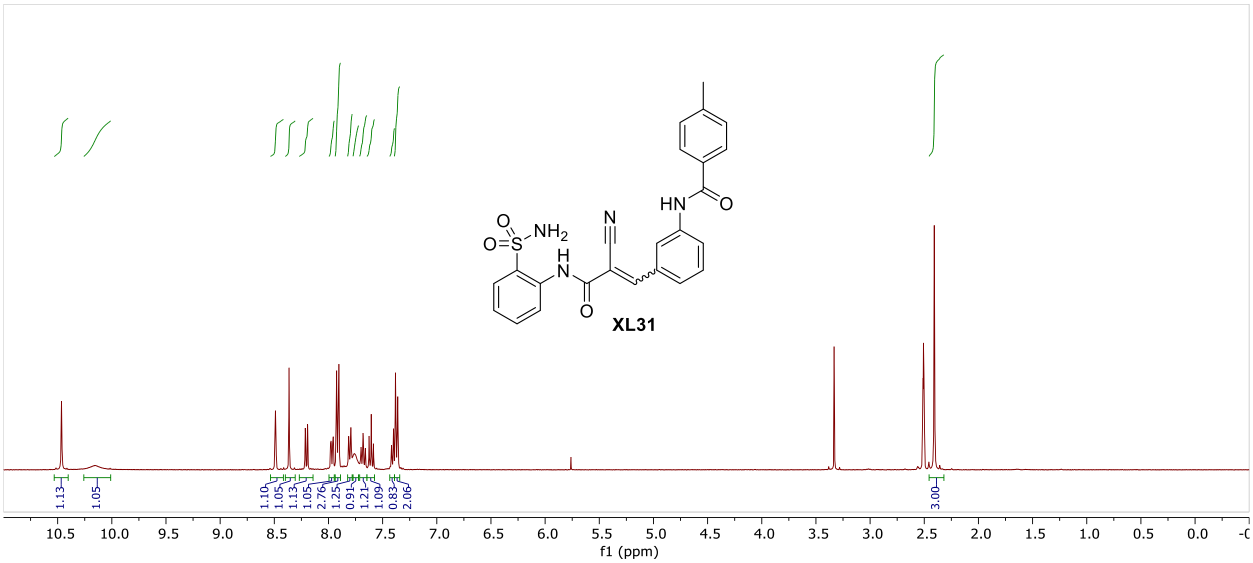


**XL31** $^{13}$C NMR in DMSO-$d_6$

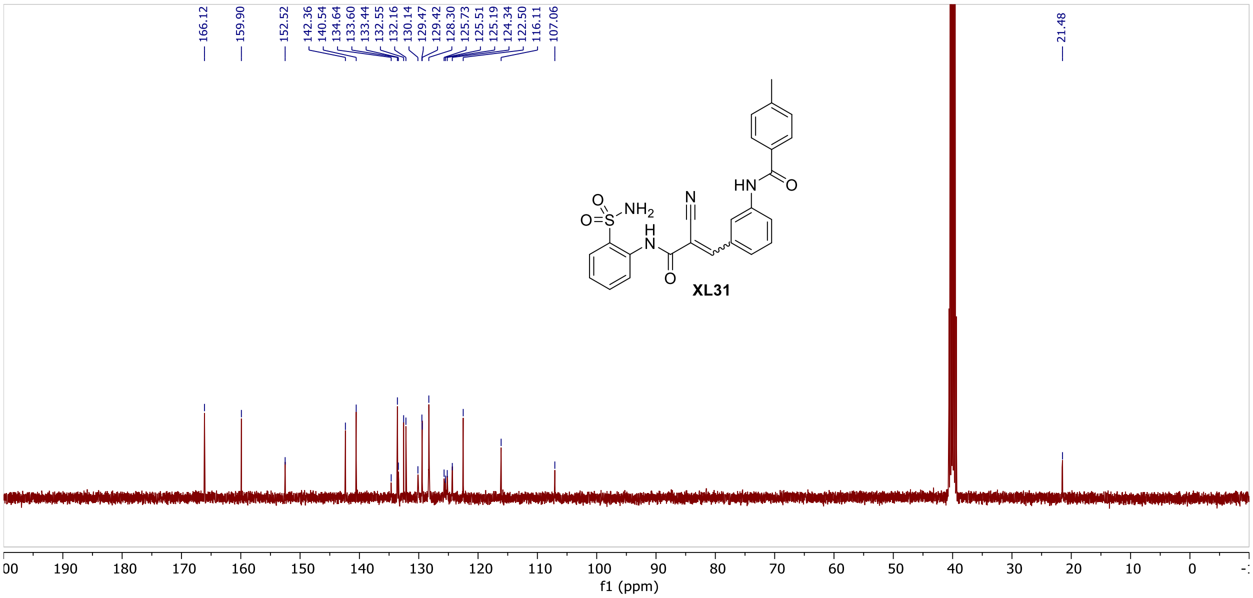

**XL32** $^{13}$C NMR in DMSO-$d_6$

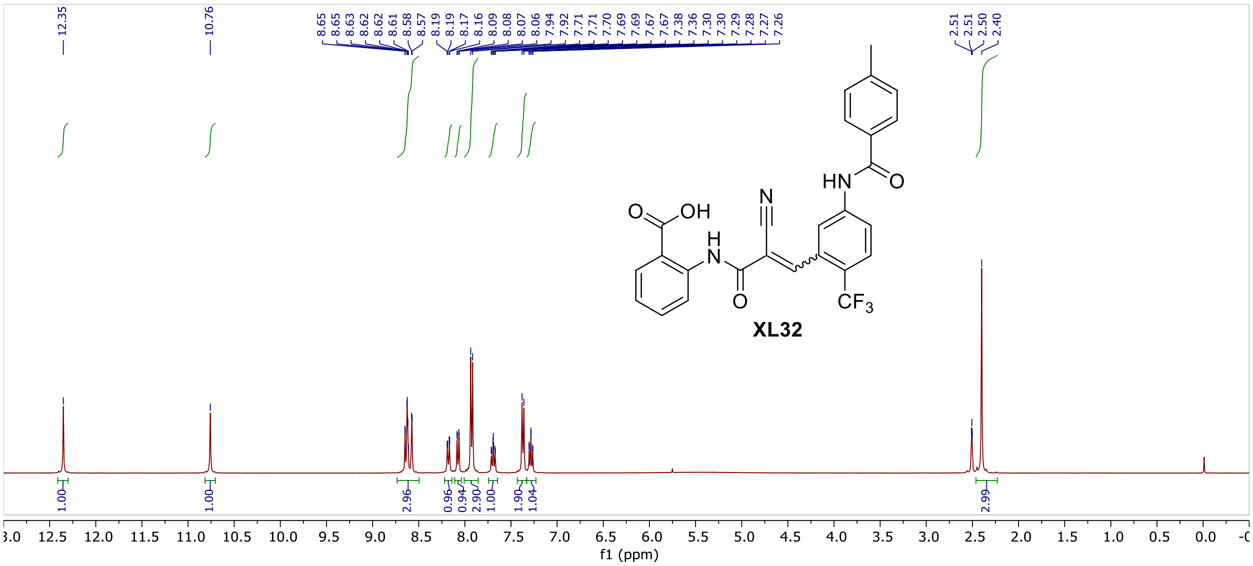


**XL32** $^{13}$C NMR in DMSO-$d_6$

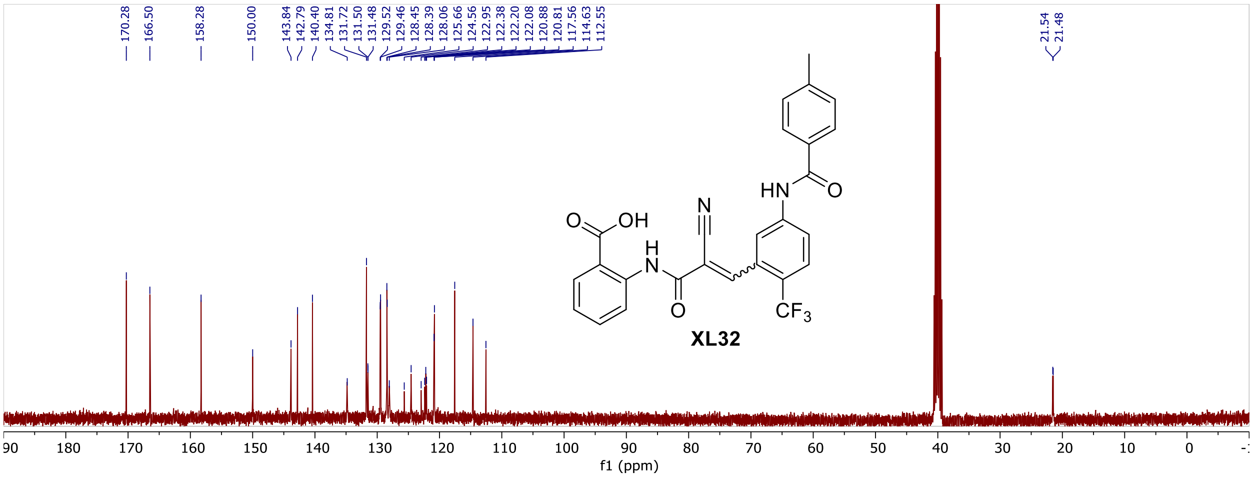


**XL32** $^{19}$F NMR in DMSO-$d_6$

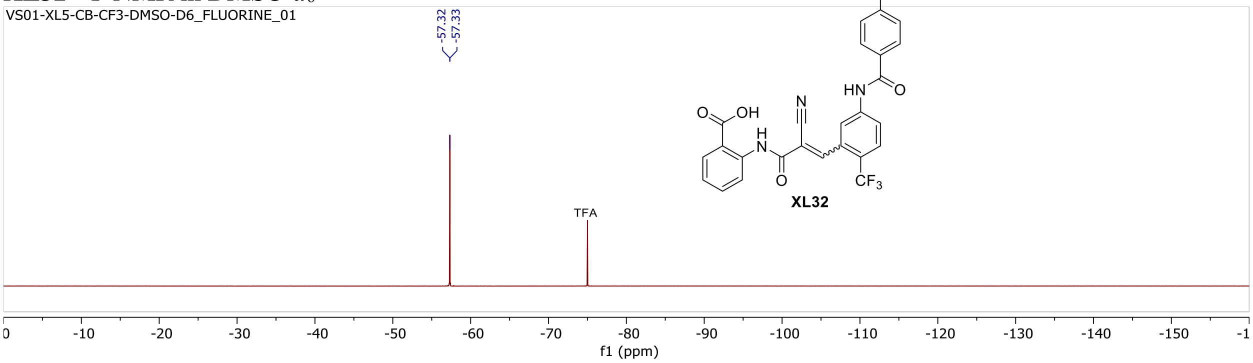

**XL33** $^{1}$H NMR in DMSO-$d_6$

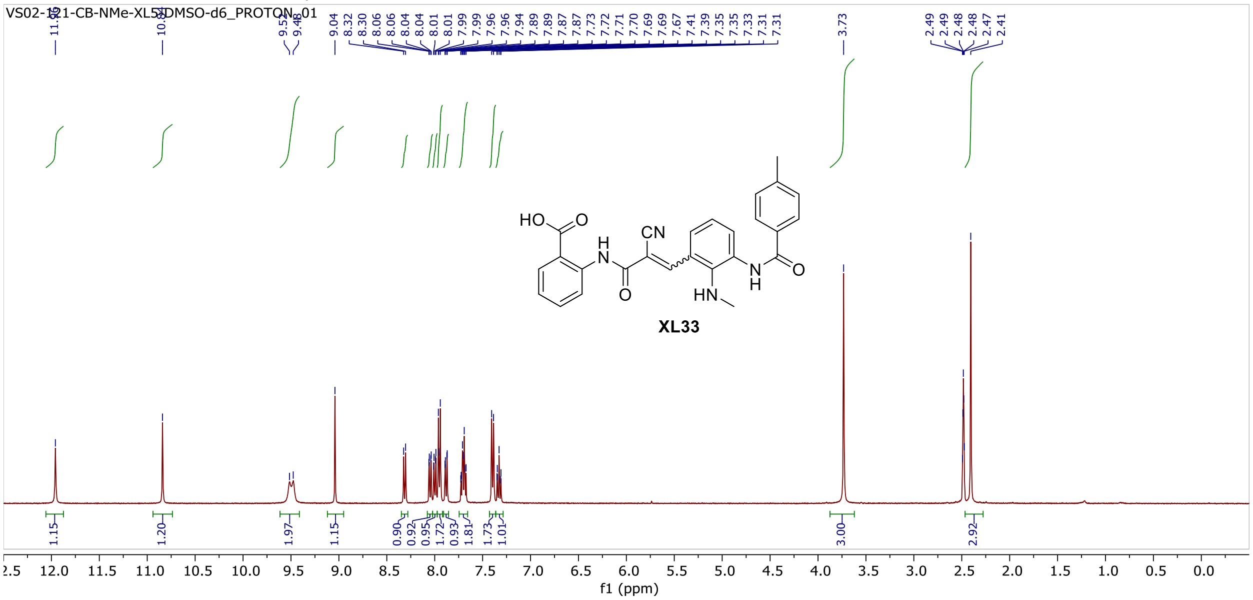


**XL33** $^{13}$C NMR in DMSO-$d_6$

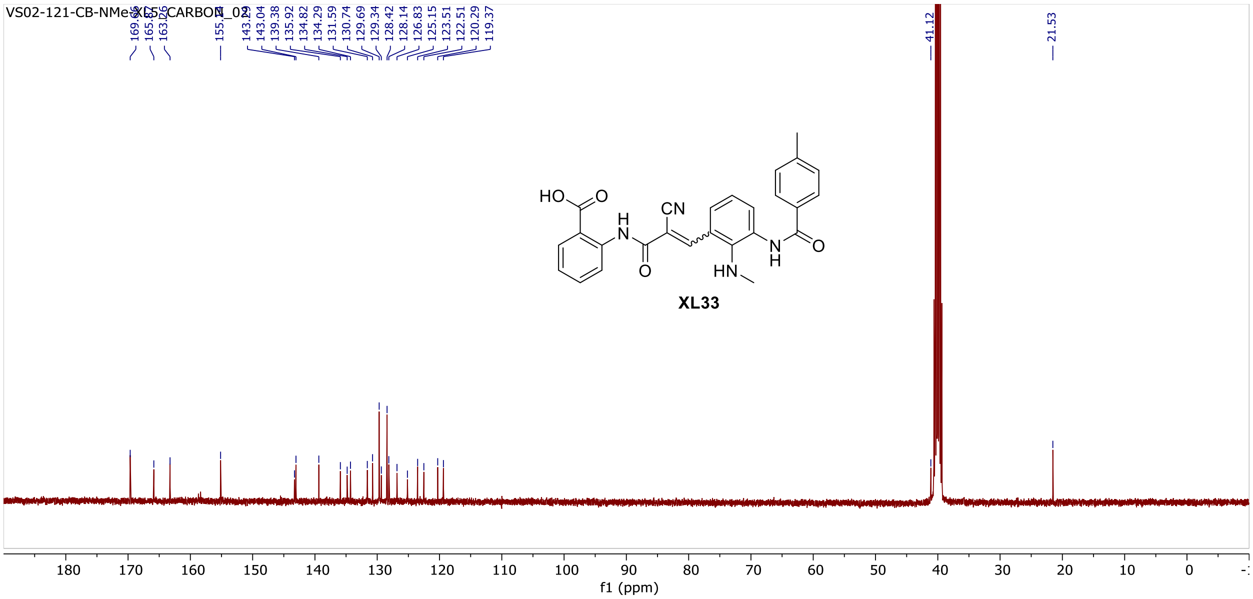

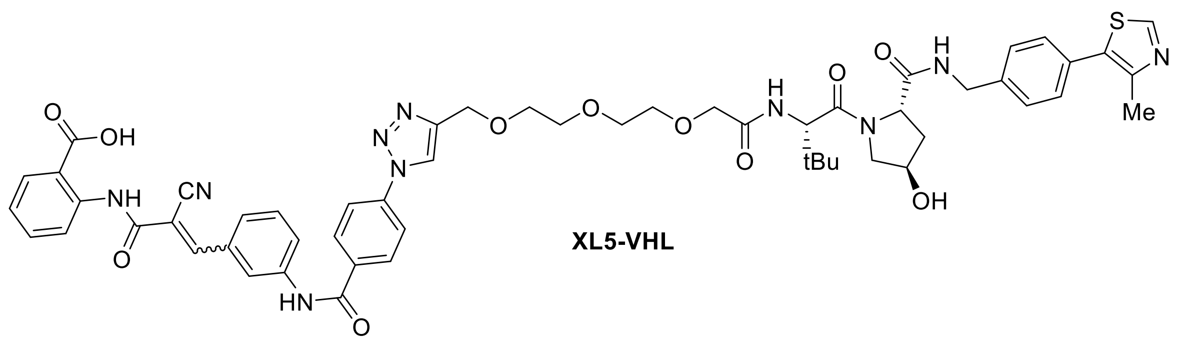


**XL5-VHL**

**XL5-VHL** $^{1}$H NMR in DMSO-$d_6$

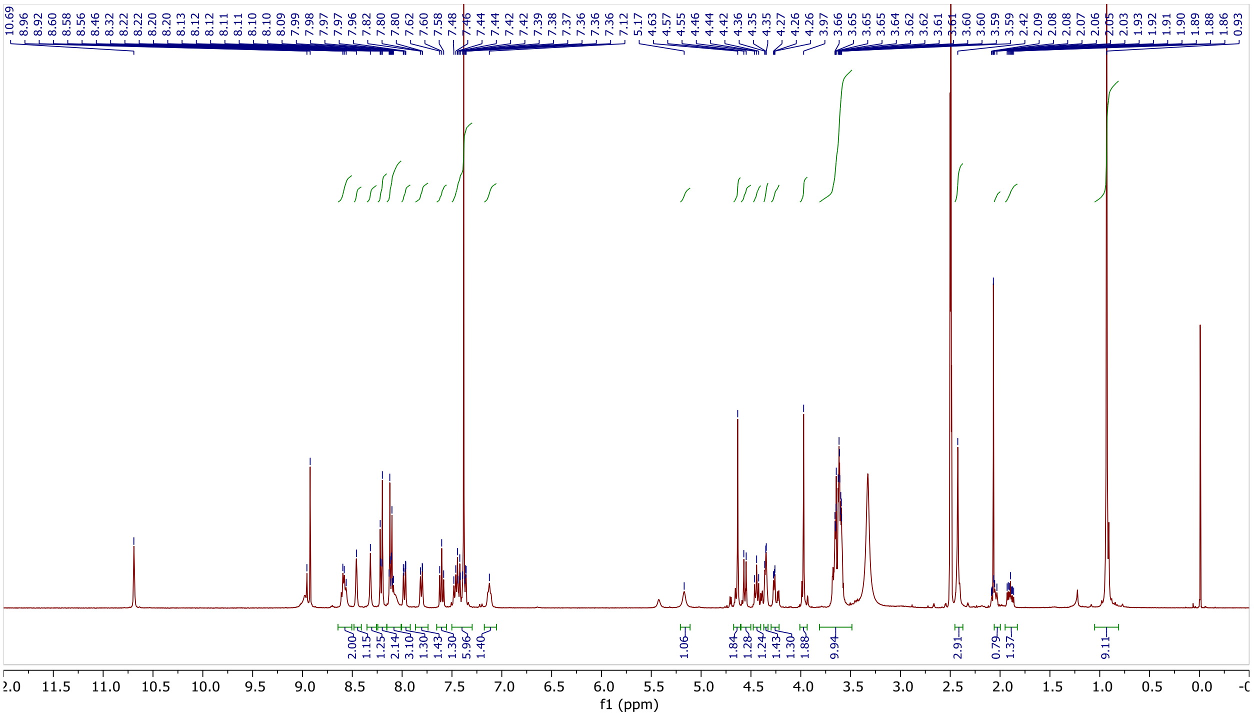


**XL5-VHL** $^{13}$C NMR in DMSO-$d_6$

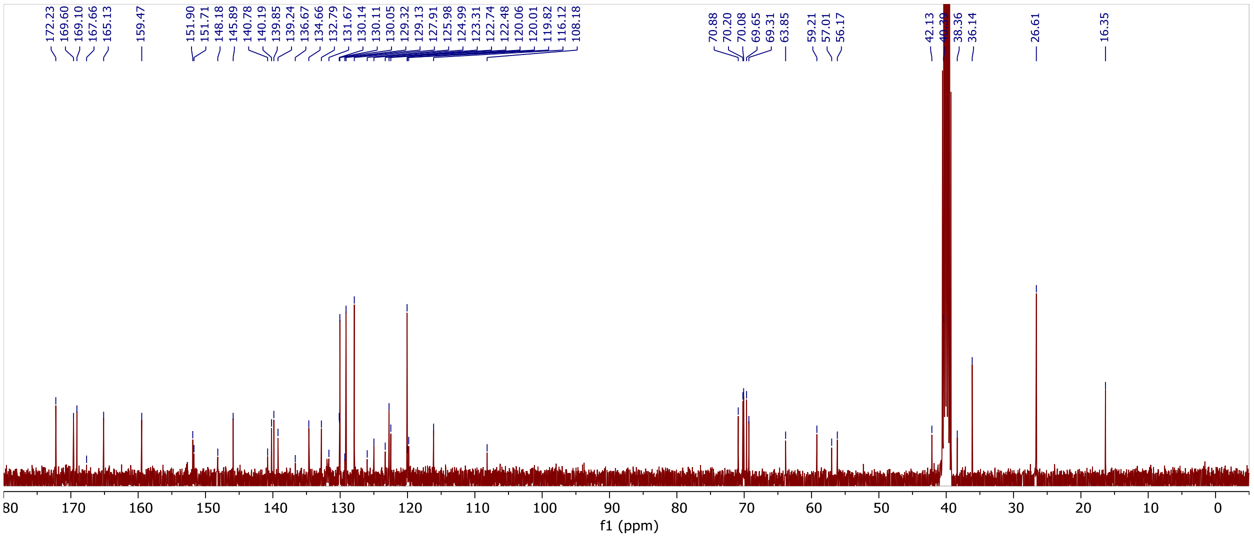

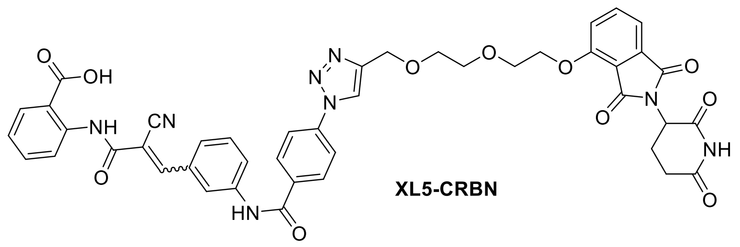


**XL5-CRBN** $^{1}$H NMR in DMSO-$d_6$

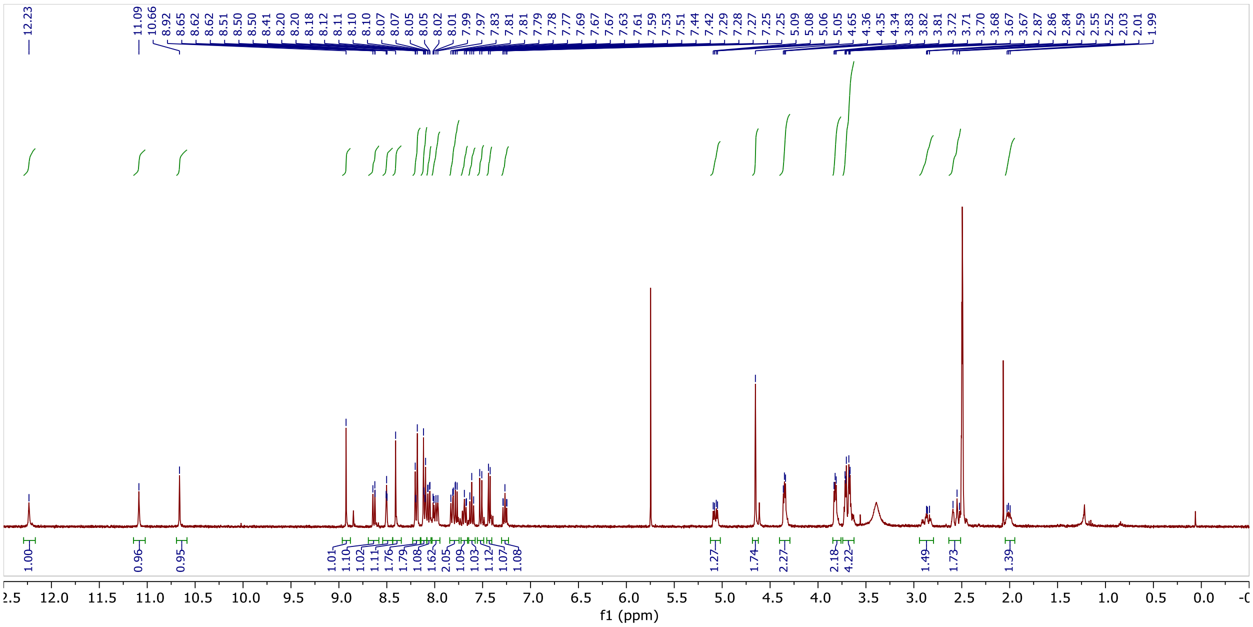


**XL5-CRBN** $^{13}$C NMR in DMSO-$d_6$

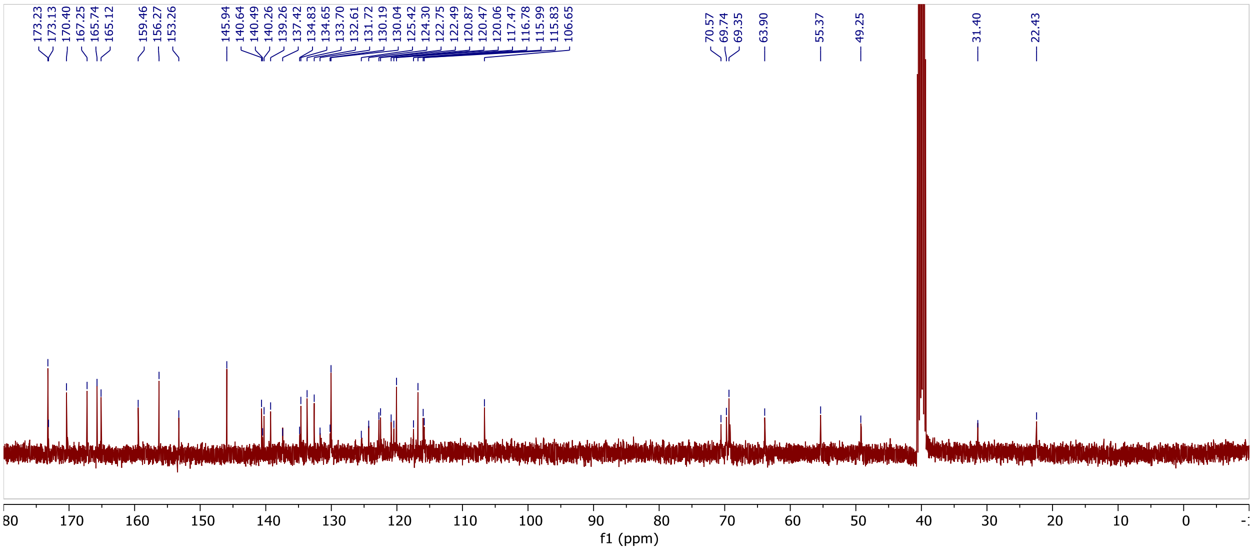

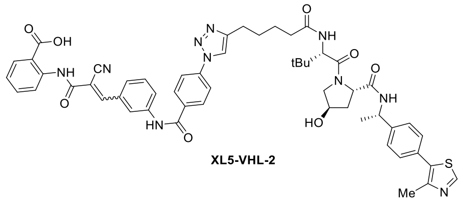

XL5-VHL-2

## XL5-VHL-2 $^{1}$H NMR in DMSO-$d_6$

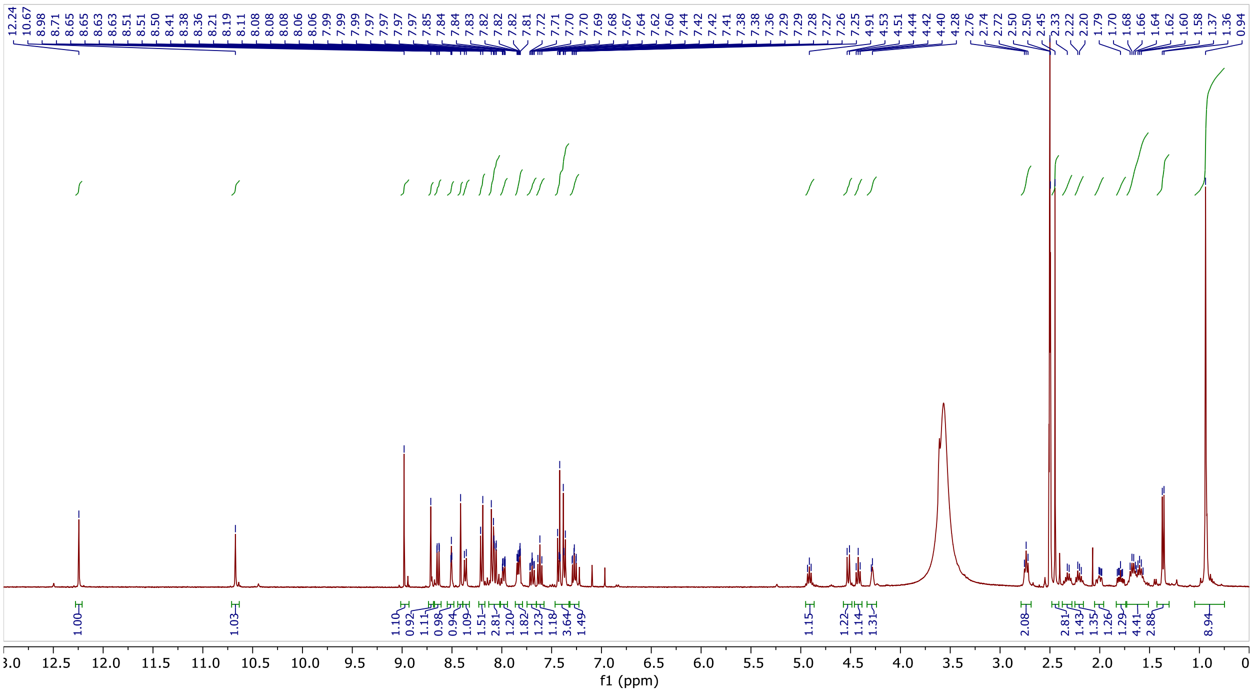

## XL5-VHL-2 $^{13}$C NMR in DMSO-$d_6$

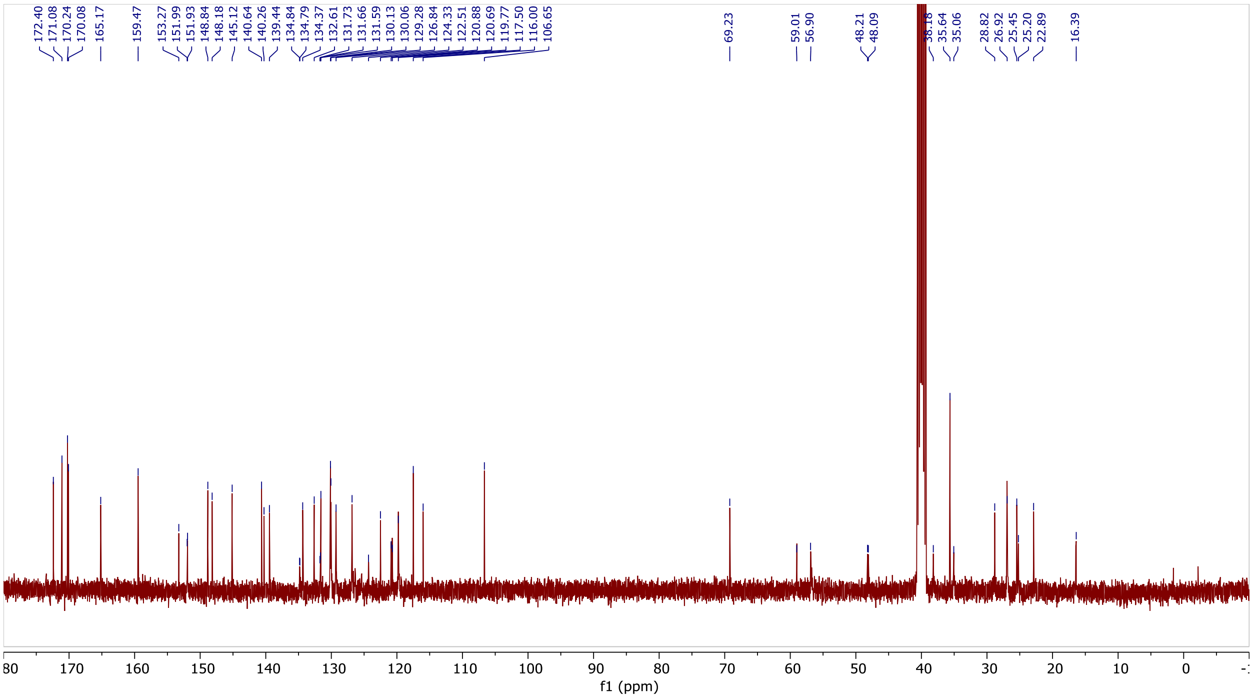

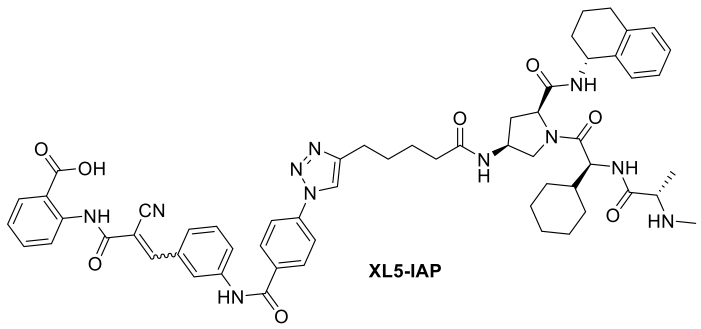

**XL5-IAP**

**XL5-IAP** $^{1}H$ NMR in DMSO-$d_6$

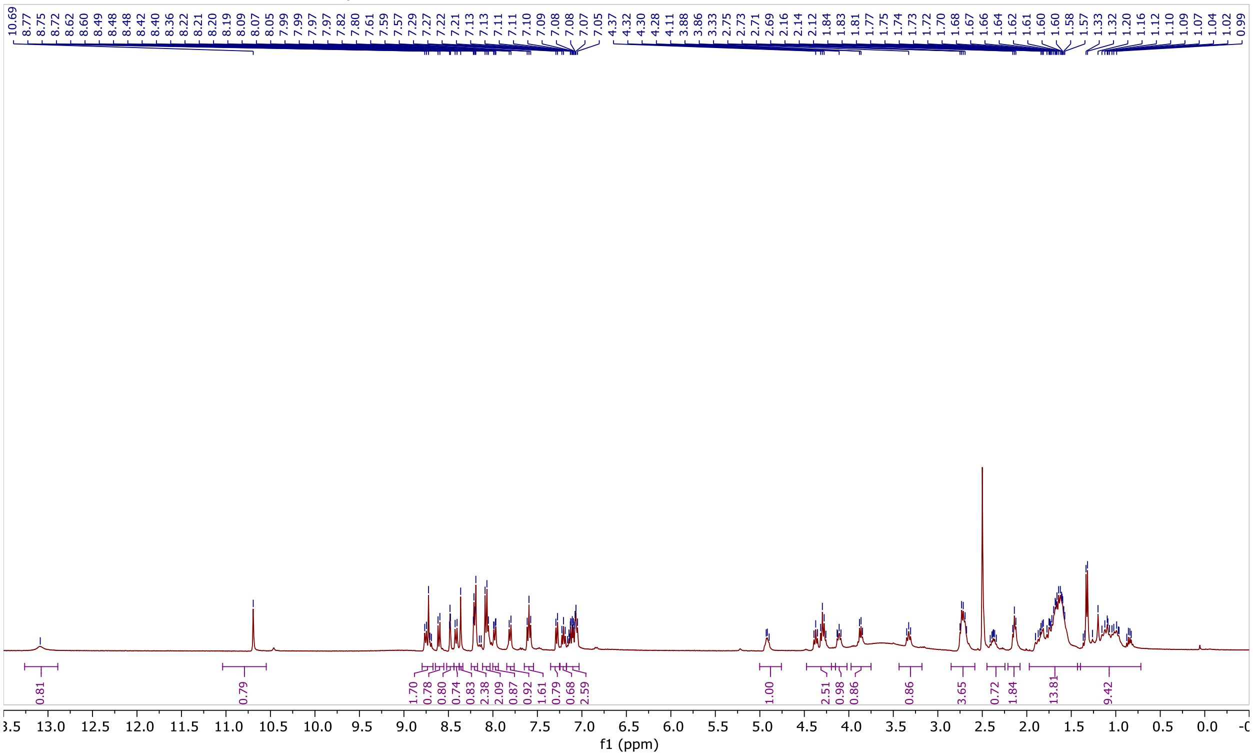


**XL5-IAP** $^{13}C$ NMR in DMSO-$d_6$

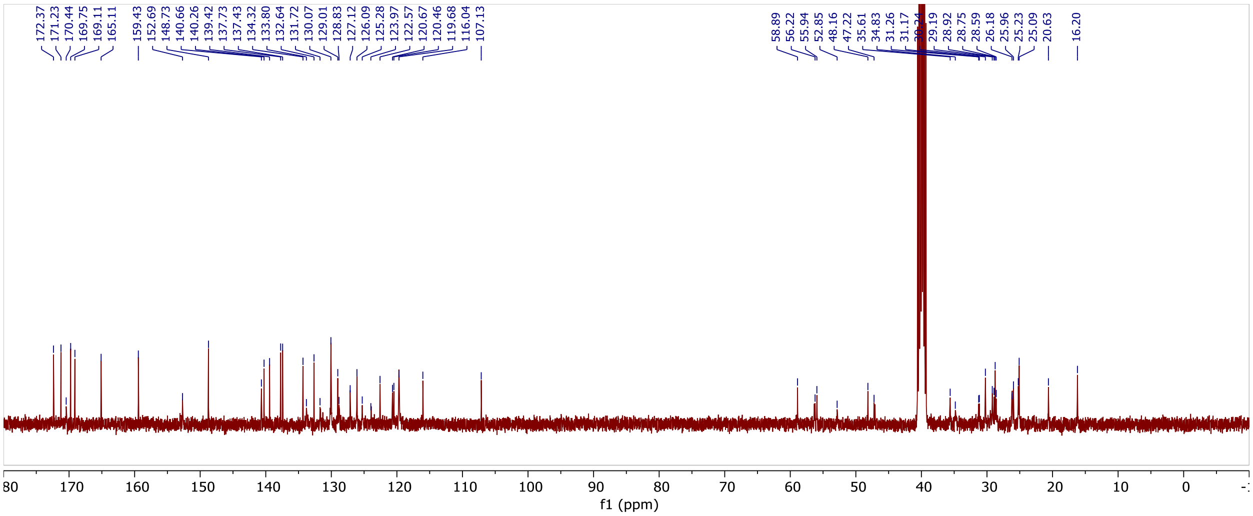

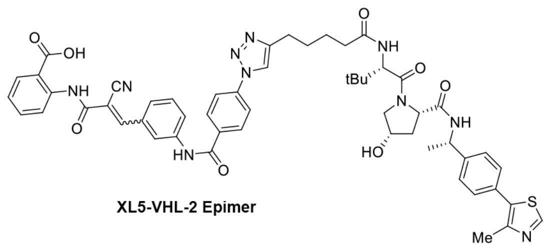


**XL5-VHL-2 Epimer**

## XL5-VHL-2 Epimer $^{1}$H NMR in DMSO-$d_6$

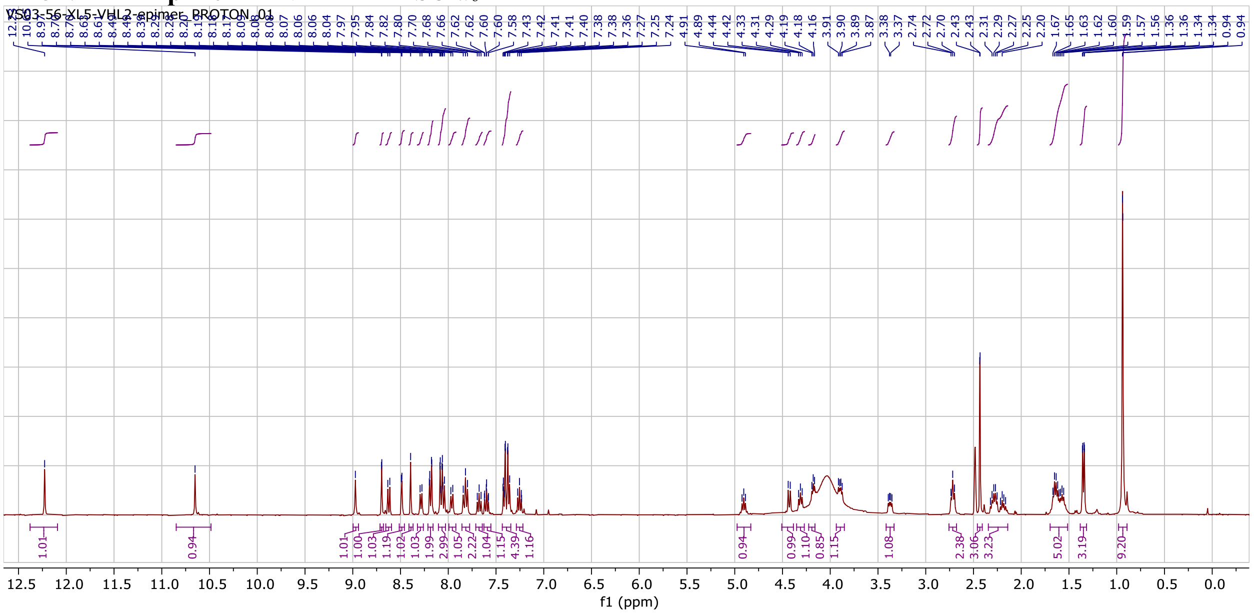


## XL5-VHL-2 Epimer $^{13}$C NMR in DMSO-$d_6$

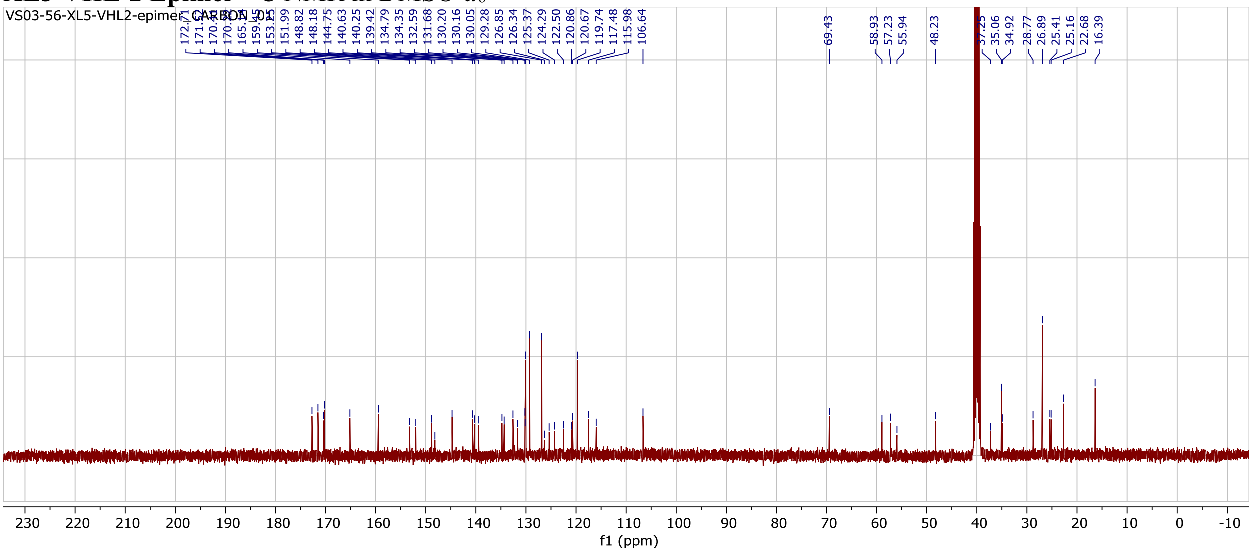

Supplement: Supplementary file 1 — Supplementary Information [file 41467_2021_27570_MOESM1_ESM.pdf]
