# Supplementary material for: Structure-guided bifunctional molecules hit a DEUBAD-lacking hRpn13 species upregulated in multiple myeloma
Source: Nat Commun. 2021 Dec 16;12:7318. doi: 10.1038/s41467-021-27570-4 (PMC8677766; doi:10.1038/s41467-021-27570-4)
Supplement: Supplementary file 2 — Description of Additional Supplementary Files [file 41467_2021_27570_MOESM2_ESM.pdf]

Description of Additional Supplementary Files

Supplementary Data 1: SQANTI3 analyses of RNA PacBio and Illumina sequencing results for the *ADRM1* gene of the RPMI 8226 WT, trRpn13-MM1 and trRpn13-MM2 cell lines.
