# Supplementary material for: Structure-guided bifunctional molecules hit a DEUBAD-lacking hRpn13 species upregulated in multiple myeloma
Source: Nat Commun. 2021 Dec 16;12:7318. doi: 10.1038/s41467-021-27570-4 (PMC8677766; doi:10.1038/s41467-021-27570-4)
Supplement: Supplementary file 4 — Reporting Summary [file 41467_2021_27570_MOESM4_ESM.pdf]

nature portfolio

Corresponding author(s): Kylie J. Walters

Last updated by author(s): 11.18.21

Reporting Summary

Nature Portfolio wishes to improve the reproducibility of the work that we publish. This form provides structure for consistency and transparency in reporting. For further information on Nature Portfolio policies, see our Editorial Policies and the Editorial Policy Checklist.

Statistics

For all statistical analyses, confirm that the following items are present in the figure legend, table legend, main text, or Methods section.

n/a	Confirmed	
☐	☒	The exact sample size (*n*) for each experimental group/condition, given as a discrete number and unit of measurement
☐	☒	A statement on whether measurements were taken from distinct samples or whether the same sample was measured repeatedly
☐	☒	The statistical test(s) used AND whether they are one- or two-sided<br>*Only common tests should be described solely by name; describe more complex techniques in the Methods section.*
☒	☐	A description of all covariates tested
☒	☐	A description of any assumptions or corrections, such as tests of normality and adjustment for multiple comparisons
☐	☒	A full description of the statistical parameters including central tendency (e.g. means) or other basic estimates (e.g. regression coefficient) AND variation (e.g. standard deviation) or associated estimates of uncertainty (e.g. confidence intervals)
☐	☒	For null hypothesis testing, the test statistic (e.g. *F*, *t*, *r*) with confidence intervals, effect sizes, degrees of freedom and *P* value noted<br>*Give P values as exact values whenever suitable.*
☒	☐	For Bayesian analysis, information on the choice of priors and Markov chain Monte Carlo settings
☒	☐	For hierarchical and complex designs, identification of the appropriate level for tests and full reporting of outcomes
☒	☐	Estimates of effect sizes (e.g. Cohen's *d*, Pearson's *r*), indicating how they were calculated

Our web collection on statistics for biologists contains articles on many of the points above.

Software and code

Policy information about availability of computer code

Data collection	ICM-Pro (Molsoft LCC) software, Bruker TopSpin, Prometheus NT.48 instrument, MicroCal iTC200 system, 6520 Accurate-Mass Q-TOF LC/MS system, TSQ Quantiva triple quadrupole mass spectrometer , Lonza 4D Nucleofector system, CLARIOstar (BMG LABTECH), PacBio Sequel II system, Illumina NextSeq instrument, Waters Acquity UPLC system coupled with a fluorescence detector, nanoflow liquid chromatography (Thermo UltimateTM 3000RSLC nano LC system) coupled to an Orbitrap Eclipse mass spectrometer
Data analysis	NMRpipe, XEASY6.4, XPLOR-NIH 2.50, PROCHECK-NMR, MOLMOL 2K.2, PyMOL 1.7.6.4, GraphPad Prism8, MicroCal Origin 7.0, Mass Hunter Workstation (version B.06.01), Mass Hunter Qualitative Analysis software (version B.07.00) , Image Studio (version 2.5.2, Licor), HiSeq Real Time Analysis software (RTA 2.11.3) , Illumina bcl2fastq2.17 , Cutadapt (version 1.18), STAR (version 2.7.0f), RSEM (version 1.3.1), isoform sequencing (IsoSeq v3), minimap2 software, squanti3 software, Proteome Discoverer 2.4 software, percolator software, Microsoft Excel or R

For manuscripts utilizing custom algorithms or software that are central to the research but not yet described in published literature, software must be made available to editors and reviewers. We strongly encourage code deposition in a community repository (e.g. GitHub). See the Nature Portfolio guidelines for submitting code & software for further information.

Data

Policy information about availability of data

All manuscripts must include a data availability statement. This statement should provide the following information, where applicable:

- Accession codes, unique identifiers, or web links for publicly available datasets
- A description of any restrictions on data availability
- For clinical datasets or third party data, please ensure that the statement adheres to our policy

The structural coordinates and chemical shift data for XL5-ligated hRpn13 Pru in this study has been deposited in the Protein Data Bank (PDB) and Biological

Magnetic Resonance Data Bank (BMRB) under accession codes 7KXI (https://www.rcsb.org/structure/unreleased/7KXI) and 30824 (https://bmrb.io/data_library/summary/index.php?bmrbId=30824). Source data are provided with this paper. PDB 6CO4 (https://www.rcsb.org/structure/6CO4), 5IRS (https://www.rcsb.org/structure/5IRS), 2KR0 (https://www.rcsb.org/structure/2KR0) are used in this study.

Field-specific reporting

Please select the one below that is the best fit for your research. If you are not sure, read the appropriate sections before making your selection.

- [x] Life sciences
- [ ] Behavioural & social sciences
- [ ] Ecological, evolutionary & environmental sciences

For a reference copy of the document with all sections, see nature.com/documents/nr-reporting-summary-flat.pdf

Life sciences study design

All studies must disclose on these points even when the disclosure is negative.

Sample size	No statistical methods were used to predetermine sample size. Sample size for each experiment was based on the literature or previous experiences to get statistical significance or reproductivity. For structure calculations, each stereoisomer was used as a starting structure for iterative simulated annealing to generate 200 initial structures, from which twenty were chosen based on criteria of no NOE, dihedral or torsion angle violation and lowest energy.
Data exclusions	No data were excluded from the analysis
Replication	Biophysical experiments including 2D NMR , DSF, ITC, LC-MS were repeated at least one time. Experiments using mammalian cells in Fig. 4c, 4f, 5a-d, 6a, 6f, 7a-d and Supplementary Fig. 9 were repeated at least one time. All replications were consistent. Experiment in Fig. 5e, 6d or Supplementary 10a-b was performed one time.
Randomization	This study did not allocate experimental groups thus randomization is not required in the reported experiments.
Blinding	Blinding was not required in this study due to no human, animal or behavioral experiments performed. All experiments were done with appropriate positive or negative controls as indicated.

Reporting for specific materials, systems and methods

We require information from authors about some types of materials, experimental systems and methods used in many studies. Here, indicate whether each material, system or method listed is relevant to your study. If you are not sure if a list item applies to your research, read the appropriate section before selecting a response.

Materials & experimental systems

n/a	Involved in the study	
☐	☒	Antibodies
☐	☒	Eukaryotic cell lines
☒	☐	Palaeontology and archaeology
☒	☐	Animals and other organisms
☒	☐	Human research participants
☒	☐	Clinical data
☒	☐	Dual use research of concern

Methods

n/a	Involved in the study	
☒	☐	ChIP-seq
☒	☐	Flow cytometry
☒	☐	MRI-based neuroimaging

Antibodies

Antibodies used	Antibodies (dilutions) used in this study include primary antibodies anti-hRpn13 (100-200) (Abcam ab157185, 1:5,000), anti-hRpn13 (350-407) (Abcam ab157218, 1:2,000, 1:5,000), anti-hRpn2 (Abcam ab2941, 1:1,000), anti-hRpt3 (Abcam ab140515, 1:1,000), anti-UCHL5 (Abcam ab133508, 1:2,000), anti-beta-actin (Cell Signaling Technology 4970s or 3700s, 1:3,000, 1:5000 or 1:10,000), anti-cleaved caspase-9 (Cell Signaling, 52873s, 1:500), anti-ubiquitin (P4D1) (Cell Signaling, 3936s, 1/1000) and anti-GST (Cell Signaling, 2625s, 1:10,000) and secondary antibodies anti-mouse (Sigma-Aldrich, A9917, 1:3,000 or 1:4,000), anti-rabbit (Life Technologies, A16110, 1:4,000, 1:5,000, 1:10,000 or 1:20,000) and anti-native rabbit (Sigma-Aldrich, R3155, 1:1000) antibodies.
Validation	All antibodies used in this study were described and validated in the reported papers.<br>Lu, X. et al. Structure of the Rpn13-Rpn2 complex provides insights for Rpn13 and Uch37 as anticancer targets. Nat Commun 8:15540 (2017).<br>Osei-Amponsa, V. et al. Impact of Losing hRpn13 Pru or UCHL5 on Proteasome Clearance of Ubiquitinated Proteins and RA190 Cytotoxicity. Mol Cell Biol 40:N/A (2020).<br>Buel, G.R. et al. Structure of E3 ligase E6AP with a proteasome-binding site provided by substrate receptor hRpn10. Nat Commun 11, 1291 (2020).

Huang ZN et al. Adhesion Regulating Molecule 1 Mediates HAP40 Overexpression-Induced Mitochondrial Defects. Int J Biol Sci 13:1420-1437 (2017).
Guo, S. et al. TRIB2 modulates proteasome function to reduce ubiquitin stability and protect liver cancer cells against oxidative stress. Cell Death Dis 12:42 (2021).
Kontou, G. et al. KCC2 is required for the survival of mature neurons but not for their development. J Biol Chem 296, 100364. 10.1016/j.jbc.2021.100364. (2021)
Yin, S. et al. PRMT5-mediated arginine methylation activates AKT kinase to govern tumorigenesis. Nat Commun 12, 3444. 10.1038/s41467-021-23833-2. (2021)

Eukaryotic cell lines

Policy information about cell lines

Cell line source(s)	The HCT116 WT (ATCC®CCL-247™), RPMI 8226 (ATCC® CCL-155™), Hs27 (ATCC® CRL-1634™), SK-OV-3 (ATCC®HTB-77), MM.1S (ATCC® CRL-2974™) and NCI-H929 (ATCC® CRL-9608™) cell lines were purchased from the American Tissue Culture Collection; HCT116 trRpn13 cells were generated and described as part of a previous study. RPMI 8226 trRpn13-MM1 and trRpn13 MM2 were generated by using the CRISPR/Cas9 system.
Authentication	None of the commercial cell lines used in this study were further authenticated. RPMI 8226 trRpn13-MM1 and trRpn13 MM2 cell lines were validated by sequencing PCR-amplified genomic DNA and RNA PacBio sequencing in addition to Western blotting. HCT116 trRpn13 cell line was validated by sequencing PCR-amplified genomic DNA and RNA PacBio sequencing in addition to Western blotting, which was described in our previous work (Osei-Amponsa V et al. Impact of Losing hRpn13 Pru or UCHL5 on Proteasome Clearance of Ubiquitinated Proteins and RA190 Cytotoxicity. Mol Cell Biol 40:N/A (2020).).
Mycoplasma contamination	The cell lines were not tested for mycoplasma contamination.
Commonly misidentified lines (See ICLAC register)	No cell lines in this study were commonly misidentified lines.
